# Supplementary material for: The flashfm approach for fine-mapping multiple quantitative traits
Source: Nat Commun. 2021 Oct 22;12:6147. doi: 10.1038/s41467-021-26364-y (PMC8536717; doi:10.1038/s41467-021-26364-y)

The Flashfm Approach for Fine-mapping Multiple Quantitative Traits – Supplementary Information

N Hernandez, J Soenksen, P Newcombe, M Sandhu, I Barroso, C Wallace, JL Asimit

Supplementary Section 1: FLexible And Shared information Fine-Mapping (flashfm) model description

1.1 The multiple traits joint ABF is a function of marginal ABFs

We first suppose that we observe N individuals, each with measurements for M quantitative traits that are transformed to meet conditional normality and homogeneity assumptions, conditional on covariates. Later, we relax this so that a subset of individuals may have missing measurements for some of the traits. Here, we find expressions for the ABF of causal SNP models for joint and marginal models and show that the information from single trait analyses could be used to evaluate the joint ABF.

To find expressions of the log(ABF) for each of the joint and marginal models we use the approximation based on the Bayesian information criterion (BIC) from the null and causal models (BIC_0 and BIC_1, respectively)[6]. The log(ABF) approximation $(\mathrm{BIC}_0 - \mathrm{BIC}_1)/2$, is expressed in terms of log likelihoods as

$$\log(\text{ABF}) \doteq l_1 - l_0 - k\log(N)/2, \tag{1}$$

where k is the number of causal SNPs in the model and l_1 and l_0 are the log likelihoods of the causal and null models, evaluated at the maximum likelihood estimates.

An expression for the log(ABF) of a causal SNP model for a single trait is found after finding the log likelihoods for the relevant models in a Gaussian framework. Let $\mathbf{y}_j, j = 1,\ldots,M$ denote the vector of N measurements for trait j, γ_j represent a particular model with k_j SNPs for trait j and $\mathbf{X}_{\gamma_j}$ be a $N \times k_j$

matrix of genotypes scores for k_j SNPs that are present in the model γ_j for trait j. Under model γ_j with causal SNPs $\mathbf{X}_{\gamma_j}$, the log-likelihood of a single trait y_j is given by

$$\begin{aligned} l_1 &= -\frac{1}{2\hat{V}}(\mathbf{y}_j - \mathbf{X}_{\gamma_j}\hat{\boldsymbol{\beta}}_j)^T(\mathbf{y}_j - \mathbf{X}_{\gamma_j}\hat{\boldsymbol{\beta}}_j) - \frac{N}{2}\log(2\pi\hat{V}) \\ &= -\frac{N}{2}\left(1 + \log(2\pi) + \log\left(\frac{(\mathbf{y}_j - \mathbf{X}_{\gamma_j}\hat{\boldsymbol{\beta}}_j)^T(\mathbf{y}_j - \mathbf{X}_{\gamma_j}\hat{\boldsymbol{\beta}}_j)}{N}\right)\right), \end{aligned} \tag{2}$$

where $\hat{V} = \frac{1}{N}(\mathbf{y}_i - \mathbf{X}_{\gamma_j}\hat{\boldsymbol{\beta}})_j^T(\mathbf{y}_i - \mathbf{X}_{\gamma_j}\hat{\boldsymbol{\beta}}_j)$ is the MLE variance of the residuals from the fitted model.

Likewise, under the null model of no SNP associations and, without loss of generality, assuming mean 0 for the trait, the log likelihood is

$$l_0 = -\frac{N}{2}\left(1 + \log(2\pi) + \log\left(\frac{\mathbf{y}_j^T\mathbf{y}_j)}{N}\right)\right). \tag{3}$$

Then, using (2) and (3) in (1), the log(ABF) for a single trait j is

$$\begin{aligned} \log(\text{ABF}_j) &= -\frac{N}{2}\log\left(\frac{(\mathbf{y}_j - \mathbf{X}_{\gamma_j}\hat{\boldsymbol{\beta}}_j)^T(\mathbf{y}_j - \mathbf{X}_{\gamma_j}\hat{\boldsymbol{\beta}}_j)}{\mathbf{y}_j^T\mathbf{y}_j}\right) - \frac{k_j}{2}\log(N) \\ &= -\frac{N}{2}\log\left(\frac{\hat{V}_{\gamma_j}}{\hat{V}_j}\right) - \frac{k_j}{2}\log(N), \end{aligned} \tag{4}$$

where V_j is the variance of trait j and $\hat{V}_{\gamma_j}$ is the residual variance from model γ_j.

Next, consider M traits that each have a possible model with possibly overlapping causal SNPs $\mathbf{X}_{\gamma_j}$ for trait j. Let $\mathbf{Y}$ be the $N \times M$ matrix of phenotypes and denote its rows by $\mathbf{y}_{i\cdot}$ (M-vector of trait values for individual i) and columns by $\mathbf{y}_{\cdot j}$ (N-vector of trait j values). Under the null model for all traits, the joint log likelihood for M traits is

$$\begin{aligned} l_0^M &= -\frac{1}{2}\sum_{i=1}^{N}\mathbf{y}_{i\cdot}^T\hat{\Sigma}_0^{-1}\mathbf{y}_{i\cdot} - \frac{N}{2}\log((2\pi)^M|\hat{\Sigma}_0|) \\ &= -\frac{N}{2}(M + M\log(2\pi) + \log(|\hat{\Sigma}_0|), \end{aligned} \tag{5}$$

where $\hat{\Sigma}_0 = \frac{1}{N}\sum_{i=1}^{N}\mathbf{y}_{i\cdot}\mathbf{y}_{i\cdot}^T$ is the MLE of the covariance matrix under the null model, having element (i,j) given by $\frac{1}{N}\mathbf{y}_i^T\mathbf{y}_j$ (assuming mean 0 for all traits), and $\frac{NM}{2}$ is obtained by using properties of a scalar and

the trace of a matrix.

Likewise, under model γ_j with k_j causal SNPs $\mathbf{X}_{\gamma_j}$ for trait j the joint log likelihood is

$$l_1^M = -\frac{N}{2}(M + M\log(2\pi) + \log(|\hat{\Sigma}_1|), \tag{6}$$

where $\hat{\Sigma}_1$ is the MLE of the covariance matrix under this model (covariance matrix of residuals) with element (i,j) given by $\frac{1}{N}(\mathbf{y}_i - \mathbf{X}_{\gamma_i}\hat{\boldsymbol{\beta}}_i)^T(\mathbf{y}_j - \mathbf{X}_{\gamma_j}\hat{\boldsymbol{\beta}}_j)$. Then it follows from (1) with (5) and (6) that the log(ABF) of the joint model containing models γ_j for trait j is

$$\log(\mathsf{ABF}^{\mathsf{M}}) = -\frac{N}{2}\log(|\hat{\Sigma}_1\hat{\Sigma}_0^{-1}|) - \frac{K}{2}\log(N), \tag{7}$$

where $K = \sum_{i=1}^{M} k_i$ is the total number of SNP effects in the joint model. When there are two traits

$$\begin{aligned}|\hat{\Sigma}_1\hat{\Sigma}_0^{-1}| &= \begin{vmatrix}(\mathbf{y}_1 - \mathbf{X}_{\gamma_1}\hat{\boldsymbol{\beta}}_1)^T(\mathbf{y}_1 - \mathbf{X}_{\gamma_1}\hat{\boldsymbol{\beta}}_1) & (\mathbf{y}_1 - \mathbf{X}_{\gamma_1}\hat{\boldsymbol{\beta}}_1)^T(\mathbf{y}_2 - \mathbf{X}_{\gamma_2}\hat{\boldsymbol{\beta}}_2) \\ (\mathbf{y}_1 - \mathbf{X}_{\gamma_1}\hat{\boldsymbol{\beta}}_1)^T(\mathbf{y}_2 - \mathbf{X}_{\gamma_2}\hat{\boldsymbol{\beta}}_2) & (\mathbf{y}_2 - \mathbf{X}_{\gamma_2}\hat{\boldsymbol{\beta}}_2)^T(\mathbf{y}_2 - \mathbf{X}_{\gamma_2}\hat{\boldsymbol{\beta}}_2)\end{vmatrix} \begin{vmatrix}\mathbf{y}_1^T\mathbf{y}_1 & \mathbf{y}_1^T\mathbf{y}_2 \\ \mathbf{y}_1^T\mathbf{y}_2 & \mathbf{y}_2^T\mathbf{y}_2\end{vmatrix}^{-1} \\ &= \begin{vmatrix} g_1 & h_{12} \\ h_{12} & g_2 \end{vmatrix} \begin{vmatrix} V_1 & C_{12} \\ C_{12} & V_2 \end{vmatrix}^{-1}\end{aligned}$$

where, $g_i = \frac{1}{N}(\mathbf{y}_i - \mathbf{X}_{\gamma_i}\hat{\boldsymbol{\beta}}_i)^T(\mathbf{y}_i - \mathbf{X}_{\gamma_i}\hat{\boldsymbol{\beta}}_i)$ is the residual variance of trait i, $h_{12} = \frac{1}{N}(\mathbf{y}_i - \mathbf{X}_{\gamma_i}\hat{\boldsymbol{\beta}}_i)^T(\mathbf{y}_j - \mathbf{X}_{\gamma_j}\hat{\boldsymbol{\beta}}_j)$ is the residual covariance for traits i and j, and C_{12} is the sample (unbiased) covariance between traits 1 and 2. Using (4), we obtain an expression for g_i that is a function of log(ABF), sample size N, and the number of SNPs in model γ_j, k_j

$$g_j = \exp(-\frac{2}{N}\left(\log(\mathsf{ABF}_j) + \frac{k_j}{2}\log(N)\right)V_j. \tag{8}$$

Then, using (4), the sum of the log(ABF) for M single traits simplifies to

$$\begin{aligned}
\sum_{j=1}^{M} \log(\mathsf{ABF}_j) &= -\frac{N}{2}\log\left(\frac{\prod_{j=1}^{M} g_j}{\prod_{j=1}^{M} V_j}\right) - \frac{K}{2}\log(N) \\
&= -\frac{N}{2}\log\left(\begin{vmatrix} g_1 & 0 & \cdots & 0 \\ 0 & g_2 & \cdots & 0 \\ \vdots & 0 & \ddots & \vdots \\ 0 & 0 & \cdots & g_M \end{vmatrix}\begin{vmatrix} V_1 & 0 & \cdots & 0 \\ 0 & V_2 & \cdots & 0 \\ \vdots & 0 & \ddots & \vdots \\ 0 & 0 & \cdots & V_M \end{vmatrix}^{-1}\right)
\end{aligned}$$

and it follows that the difference between the joint log(ABF) and the sum of the marginals is

$$\begin{aligned}
D_M &= \log(\mathsf{ABF}^{\mathsf{M}}) - \sum_{j=1}^{M} \log(\mathsf{ABF}_j) \\
&= -\frac{N}{2}\log\left(\begin{vmatrix} g_1 & h_{12} & \cdots & h_{1M} \\ h_{12} & g_2 & \cdots & h_{2M} \\ \vdots & h_{i2} & \ddots & \vdots \\ h_{1M} & h_{2M} & \cdots & g_M \end{vmatrix}\begin{vmatrix} g_1 & 0 & \cdots & 0 \\ 0 & g_2 & \cdots & 0 \\ \vdots & 0 & \ddots & \vdots \\ 0 & 0 & \cdots & g_M \end{vmatrix}^{-1}\right) \\
&\quad +\frac{N}{2}\log\left(\begin{vmatrix} V_1 & C_{12} & \cdots & C_{1M} \\ C_{12} & V_2 & \cdots & C_{2M} \\ \vdots & C_{i2} & \ddots & \vdots \\ C_{1M} & C_{2M} & \cdots & V_M \end{vmatrix}\begin{vmatrix} V_1 & 0 & \cdots & 0 \\ 0 & V_2 & \cdots & 0 \\ \vdots & 0 & \ddots & \vdots \\ 0 & 0 & \cdots & V_M \end{vmatrix}^{-1}\right) \\
&= -\frac{N}{2}\left(\log\begin{vmatrix} 1 & \frac{h_{12}}{g_2} & \cdots & \frac{h_{1M}}{g_M} \\ \frac{h_{12}}{g_1} & 1 & \cdots & \frac{h_{2M}}{g_M} \\ \vdots & \frac{h_{i2}}{g_2} & \ddots & \vdots \\ \frac{h_{1M}}{g_1} & \frac{h_{2M}}{g_2} & \cdots & 1 \end{vmatrix} - \log\begin{vmatrix} 1 & \frac{C_{12}}{V_2} & \cdots & \frac{C_{1M}}{V_M} \\ \frac{C_{12}}{V_1} & 1 & \cdots & \frac{C_{2M}}{V_M} \\ \vdots & \frac{C_{i2}}{V_2} & \ddots & \vdots \\ \frac{C_{1M}}{V_1} & \frac{C_{2M}}{V_2} & \cdots & 1 \end{vmatrix}\right). \qquad (9)
\end{aligned}$$

where the second determinant is a constant C with respect to the samples. Thus, the joint ABF is proportional to the product of the the marginal BFs and a function of the sample sizes and residual variances and covariances. Residual variances are approximated from the log(BF) for the coinciding model and trait, and residual covariances h_{ij} are approximated as described below. If traits are standardised to

have mean 0 and variance 1 and trait summary statistics are unavailable, we may use an estimate of the correlation matrix based on the GWAS summary statistics from the LD score regression approach[3].

We have $h_{ij} = \mathbf{y}_i^T \mathbf{y}_j - \mathbf{y}_i^T \mathbf{X}_{\gamma_j} \hat{\boldsymbol{\beta}}_j - \mathbf{y}_j^T \mathbf{X}_{\gamma_i} \hat{\boldsymbol{\beta}}_i + \hat{\boldsymbol{\beta}}^{*}{}_i^T \mathbf{X}_{\gamma_i \cup \gamma_j}^T \mathbf{X}_{\gamma_i \cup \gamma_j} \hat{\boldsymbol{\beta}}_j^*$, where $\mathbf{X}_{\gamma_i \cup \gamma_j}$ is the genotype matrix with the SNPs contained in models γ_i and/or γ_j (i.e. union of model SNPs) and $\hat{\boldsymbol{\beta}}_i^*$ has the same effect estimates as $\hat{\boldsymbol{\beta}}_i$ at the SNPs in γ_i and has 0 at SNPs that are only in in γ_j. The term $\mathbf{y}_i^T \mathbf{y}_j$ may be estimated from trait summary statistics. Terms of the form $\mathbf{y}_i^T \mathbf{X}_{\gamma_j} \hat{\boldsymbol{\beta}}_j = \sum_{k \in \gamma_j} S_{x_j y_i} \hat{\boldsymbol{\beta}}_{jk}$, where $S_{x_j y_i} = \mathsf{x}_j^T \mathbf{y}_i$ and $\hat{\beta}_{jk}$ is the trait j effect estimate for the kth SNP in γ_j, and $S_{x_j y_i} = \sum_{k=1}^N x_{jk} y_{ik}$ is calculated from the the single-SNP effect estimates of the kth SNP from the trait j model for trait i (i.e. $S_{x_j y_i} = \hat{\beta}_{x_j}(N-1)V_{x_j}V_{y_i} + 2 * RAF_{x_j} * \hat{\mu}_{Y_i} N$, where RAF_{x_j} is the reference allele frequency of x_j). The last term $\hat{\boldsymbol{\beta}}_i^T \mathbf{X}^T \mathbf{X} \hat{\boldsymbol{\beta}}_j$ relies on the effect estimates from the two trait models and $\mathbf{X}^T \mathbf{X}$ may be approximated from either the genotype or a suitable reference panel, as element (i, j) of the matrix is

$$(\mathbf{X}^T \mathbf{X})_{ij} \doteq N(Cov(X_i, X_j) - E(X_i)E(X_j))$$

Thus, all quantities in the final expression for D_M could be obtained from marginal analyses of the traits and summary information of the traits.

1.2 Traits not measured for all samples

It is common for only a subset of individuals to have measurements for all traits. For simplicity, consider two traits that are both measured in N individuals and let N_{i-j} be the number of individuals with trait i measured, but not trait j; the number of individuals with trait i measured is $N_i = N + N_{i-j}$. Using all N_i

samples for trait i we obtain the marginal $\log(\mathsf{ABF}_i)$ as in (4), we then have

$$\begin{aligned}
\log(\mathsf{ABF}_1)+\log(\mathsf{ABF}_2) &= -\frac{(N+N_{1-2})}{2}\log\left(\frac{\hat{V}_{\gamma_1}}{V_1}\right)-\frac{(N+N_{2-1})}{2}\log\left(\frac{\hat{V}_{\gamma_2}}{V_2}\right)\\
&\quad -\frac{k_1}{2}\log(N_1)-\frac{k_2}{2}\log(N_2)\\
&= -\frac{N}{2}\log\left(\frac{\hat{V}_{\gamma_1}\hat{V}_{\gamma_2}}{V_1V_2}\right)\\
&\quad +\frac{N_{1-2}}{N_1}\left(\log(\mathsf{ABF}_1)+\frac{k_1}{2}\log(N_1)\right)\\
&\quad +\frac{N_{2-1}}{N_2}\left(\log(\mathsf{ABF}_2)+\frac{k_2}{2}\log(N_2)\right)\\
&\quad -\frac{k_1}{2}\log(N_1)-\frac{k_2}{2}\log(N_2) \qquad (10)
\end{aligned}$$

The joint ABF is obtained in a similar manner, where extra terms are needed to account for the individuals with measurements for only one of the two traits. Let $\mathbf{y}_{i-j}$ denote the trait i measurements for individuals that do not have trait j measured. It follows that for the likelihood under the null, we have

$$\begin{aligned}
l_0 &= -N-\frac{N}{2}\log(V_1V_2-cov^2(y_1,y_2))-N\log(2\pi)\\
&\quad -\frac{N_{1-2}}{2}(1+\log(2\pi)+\log\left(\frac{\mathbf{y}_{1-2}^T\mathbf{y}_{1-2}}{N_{1-2}}\right))\\
&\quad -\frac{N_{2-1}}{2}(1+\log(2\pi)+\log\left(\frac{\mathbf{y}_{2-1}^T\mathbf{y}_{2-1}}{N_{2-1}}\right)) \qquad (11)
\end{aligned}$$

and under the models γ_1,γ_2, for traits 1 and 2, where $\hat{\boldsymbol{\beta}}_i$ is based on all N_i samples with measurements and $\hat{\boldsymbol{\beta}}_{i-j}$ is based on the N_{i-j} samples with trait i measured and not trait j , we have

$$\begin{aligned}
l_1 &= -N-N\log(2\pi)-\frac{N}{2}\log(\hat{V}_{\gamma_1}\hat{V}_{\gamma_2}-\hat{C}^2_{\gamma_1,\gamma_2})\\
&\quad -\frac{N_{1-2}}{2}(1+\log(2\pi)+\log\left(\frac{\mathbf{y}_{1-2}^T\mathbf{y}_{1-2}-\mathbf{y}_{1-2}^T\mathbf{X}_{\gamma_1}\hat{\boldsymbol{\beta}}_{1-2}}{N_{1-2}}\right))\\
&\quad -\frac{N_{2-1}}{2}(1+\log(2\pi)+\log\left(\frac{\mathbf{y}_{2-1}^T\mathbf{y}_{2-1}-\mathbf{y}_{2-1}^T\mathbf{X}_{\gamma_2}\hat{\boldsymbol{\beta}}_{2-1}}{N_{2-1}}\right)), \qquad (12)
\end{aligned}$$

From (11) and (12) we get

$$\begin{aligned} l_1 - l_0 &= -\frac{N}{2}\log\left(\frac{\hat{V}_{\gamma_1}\hat{V}_{\gamma_2} - \hat{C}^2_{\gamma_1,\gamma_2}}{V_1 V_2 - C^2_{1,2}}\right) \\ &\quad -\frac{N_{1-2}}{2}\log\left(\frac{\mathbf{y}^T_{1-2}\mathbf{y}_{1-2} - \mathbf{y}^T_{1-2}\mathbf{X}_{\gamma_1}\hat{\boldsymbol{\beta}}_{1-2}}{\mathbf{y}^T_{1-2}\mathbf{y}_{1-2}}\right) \\ &\quad -\frac{N_{2-1}}{2}\log\left(\frac{\mathbf{y}^T_{2-1}\mathbf{y}_{2-1} - \mathbf{y}^T_{2-1}\mathbf{X}_{\gamma_2}\hat{\boldsymbol{\beta}}_{2-1}}{\mathbf{y}^T_{2-1}\mathbf{y}_{2-1}}\right) \end{aligned} \tag{13}$$

Notice that $\log\left(\frac{\mathbf{y}^T_{1-2}\mathbf{y}_{1-2} - \mathbf{y}^T_{1-2}\mathbf{X}_{\gamma_1}\hat{\boldsymbol{\beta}}_{1-2}}{\mathbf{y}^T_{1-2}\mathbf{y}_{1-2}}\right)$ is the same form as the log ratio in the marginal $\log(\mathsf{ABF}_1)$ expression in (4) and is based on a subset of size N_{1-2} from the N_1 samples. Treating this log ratio based on the N_{1-2} samples as an approximation to that based on all N_1 samples, we have

$$\log\left(\frac{\mathbf{y}^T_{1-2}\mathbf{y}_{1-2} - \mathbf{y}^T_{1-2}\mathbf{X}_{\gamma_1}\hat{\boldsymbol{\beta}}_{1-2}}{\mathbf{y}^T_{1-2}\mathbf{y}_{1-2}}\right) \doteq -\frac{2}{N_1}\left(\log(\mathsf{ABF}_1) + \frac{k_1}{2}\log(N_1)\right)$$

Likewise for trait 2.

Now, using $l_1 - l_0$ in (13), $\log(\mathsf{ABF}^{\mathsf{M}}) = l_1 - l_0 - \frac{k_1}{2}\log(N_1) - \frac{k_2}{2}\log(N_2)$, where $N_j, j = 1,2$ are used, being the number of samples in the estimates for $\hat{\boldsymbol{\beta}}_j$. So,

$$\begin{aligned} \log(\mathsf{ABF}^{\mathsf{M}}) &= -\frac{N}{2}\log\left(\frac{\hat{V}_{\gamma_1}\hat{V}_{\gamma_2} - \hat{C}^2_{\gamma_1,\gamma_2}}{V_1 V_2 - C^2_{1,2}}\right) \\ &\quad +\frac{N_{1-2}}{N_1}\left(\log(\mathsf{ABF}_1) + \frac{k_1}{2}\log(N_1)\right) \\ &\quad +\frac{N_{2-1}}{N_2}\left(\log(\mathsf{ABF}_2) + \frac{k_2}{2}\log(N_2)\right) \\ &\quad -\frac{k_1}{2}\log(N_1) - \frac{k_2}{2}\log(N_2) \\ &= -\frac{N}{2}\log\left(\begin{vmatrix} g_1 & h_{12} \\ h_{12} & g_2 \end{vmatrix}\begin{vmatrix} V_1 & C_{12} \\ C_{12} & V_2 \end{vmatrix}^{-1}\right) \\ &\quad +\frac{N_{1-2}}{N_1}\log(\mathsf{ABF}_1) + \frac{N_{2-1}}{N_2}\log(\mathsf{ABF}_2) \\ &\quad -\frac{N_1 - N_{1-2}}{N_1}\frac{k_1}{2}\log(N_1) - \frac{N_2 - N_{2-1}}{N_2}\frac{k_2}{2}\log(N_2) \end{aligned} \tag{14}$$

Then, when finding the difference between the joint ABF and sum of marginal ABFs in this setting,

the additional terms accounting for missing measurements for one of the traits cancel out between the joint and sum of marginal ABFs, giving a similar form to when all traits are measured.

$$\begin{aligned} D_M &= \log(\mathsf{ABF}^{\mathsf{M}}) - \sum_{j=1}^{2} \log(\mathsf{ABF}_j) \\ &= -\frac{N}{2}\left(\log\begin{vmatrix} 1 & \dfrac{h_{12}}{g_2} \\ \dfrac{h_{12}}{g_1} & 1 \end{vmatrix} - \log\begin{vmatrix} 1 & \dfrac{C_{12}}{V_2} \\ \dfrac{C_{12}}{V_1} & 1 \end{vmatrix}\right) \end{aligned} \tag{15}$$

.

In general, for M traits, the joint $\log(\mathsf{ABF})$ is expressed as

$$\log(\mathsf{ABF}^{\mathsf{M}}) = \sum_{j=1}^{M} \log(\mathsf{ABF}_j) + D_M,$$

where D_M for two traits is as in (16) and for $M = 3,4,5$, expressions follow.

When there are more than two traits and some have missing data, additional terms to account for missing measurements are present in the expression for the $\log(\mathsf{ABF}^{\mathsf{M}})$. The derivations for 3-6 traits generalise from the two trait scenario and we use the notation δ_{ijk} to represent the term given in (9) for traits i, j, k and analogously for a larger number of traits. In addition, N_{ijk} is the number of individuals with traits i, j and k all measured, N_{ij-k} denotes the number of individuals with both traits i, j measured and not trait k, N_{i-jk} denotes the number of individuals with trait i measured, but neither of traits j and k, and analogous notation is used for more/different combinations of traits.

3 traits:

$$D_M = \delta_{123} + \frac{N_{12-3}}{N_{12}}\delta_{12} + \frac{N_{13-2}}{N_{13}}\delta_{13} + \frac{N_{23-1}}{N_{23}}\delta_{23},$$

where

$$N_{jk-l} = N_{jk} - N_{jkl};$$

$$N_{j-kl} = N_j - N_{jk} - N_{jl} + N_{123};$$

4 traits, denote $\mathscr{M} = \{1,2,3,4\}$:

$$D_M = \delta_{1234} + \sum_{j=1;k,l,m \in \mathscr{M}\setminus\{j\}}^{4} \frac{N_{klm-j}}{N_{klm}} \delta_{klm} + \sum_{j=1;k \neq j;l,m \in \mathscr{M}\setminus\{j,k\}}^{4} \frac{N_{jk-lm}}{N_{jk}} \delta_{jk},$$

where

$$N_{jkl-m} = N_{jkl} - N_{jklm};$$

$$N_{jk-lm} = N_{jk} - N_{jkl} - N_{jkm} + N_{1234};$$

5 traits, denote $\mathscr{M} = \{1,2,3,4,5\}$:

$$D_M = \delta_{12345} + \sum_{i=1;j,k,l,m \in \mathscr{M}\setminus\{j\}}^{5} \frac{N_{jklm-i}}{N_{jklm}} \delta_{jklm-i} + \sum_{i=1;j \neq i;k,l,m \in \mathscr{M}\setminus\{i,j\}}^{5} \frac{N_{ij-klm}}{N_{ij}} \delta_{ij} + \sum_{i=1;j,k \neq i;l,m \in \mathscr{M}\setminus\{i,j,k\}}^{5} \frac{N_{ijk-lm}}{N_{ijk}} \delta_{ijk}$$

where

$$N_{ijkl-m} = N_{ijkl} - N_{ijklm};$$

$$N_{ijk-lm} = N_{ijk} - N_{ijkl} - N_{ijkm} + N_{12345};$$

$$N_{ij-klm} = N_{ij} - N_{ijk} - N_{ijl} - N_{ijm} + N_{ijkl} + N_{ijkm} + N_{ijlm} - N_{12345};$$

In our flashfm software we include a “fastapprox" option that gives a quicker calculation by ignoring the extra adjustment terms. This is recommended when there are not many missing trait measurements and when a quicker answer is required; by default `fastapprox=FALSE`, but for 6 traits, only `fastapprox=TRUE` is available.

The prior probability for the joint models includes a term that gives more weight to joint models that have a shared causal variant between the traits; this term κ is derived in a combinatorial manner and is identical to that used in MFM[1]. As in MFM, a correction term τ is also included to ensure that the prior probability of a certain number of SNPs in a model is the same for any value of κ. When $\kappa = 1$,

there is no weight for joint models with shared causal variants and the flashfm PP for each model for a given trait is the same to what one would obtain from single-trait fine-mapping, which we also refer to as independent fine-mapping, as it does not make use of data from other traits.

1.3 Implementation

There are two options for implementing flashfm. If single-trait fine-mapping results have not already been obtained, they may be generated within flashfm using an extended version of JAM (Joint Analysis of Marginal summary statistics[4] - this requires GWAS summary statistics and either a genotype matrix or both a genotype covariance matrix and MAF vector from a reference panel or in-sample study. Alternatively, any single-trait fine-mapping approach that output model PPs, such as FINEMAP[2] could be used as input - this still requires GWAS summary statistics and either a genotype matrix or both a genotype covariance matrix and MAF vector from a reference panel or in-sample study.

JAM assesses the joint effect of multiple SNPs on a trait in an integrated Bayesian penalized regression framework, outputting the posterior probabilities (PP) for the multi-SNP models. This allows us to identify the models with non-negligible evidence that should be the focus when assessing joint models between multiple traits. As JAM operates on a set of tag SNPs due to colinearity issues, we have extended it such that all models are expanded by their tag SNPs in the same manner as GUESSFM[5] (https://github.com/chr1swallace/GUESSFM). This is done by substituting each tag SNP in a model by each of the SNPs that it tags so that if SNPs 1 and 2 are in a model and they each tag t_1 and t_2 SNPS, respectively, the model expands into $(t_1+1)(t_2+1)$ models, for which ABFs are found using (4; $\hat{\beta}$ for multi-SNP models are obtained from the single-SNP $\hat{\beta}$ and the genotype matrix (or reference panel) of the SNPs in the model.

Using a binomial prior distribution we may then find PPs for all of the expanded (and original) models. For ease of interpretation, we also construct SNP groups (using the snp.picker function of GUESSFM) such that SNPs in the same group could be substituted for one another; SNPs in the same group are in high LD and are rarely selected together in models. The results are then summarised in terms of SNP group PPs by summing over SNP models that fall into each SNP group model; the PP for the SNP group model $A+B$ is the sum over PPs from all models with one SNP from A and one SNP from B.

The posterior probability of model γ_1 for trait 1 is proportional to a sum of the posterior probabilities of all configurations $C_{1,j},\ j=1,\ldots,n$. Let $I_{i,j}$ be an indicator function, taking the value 1 if $\gamma_i \cap \gamma_j \neq \varnothing$ and 0 otherwise, and let $\delta_{ij} = \exp D_{ij}$ Then

$$\begin{aligned}
Pr(\gamma_i \text{ for trait 1}|\text{Data}) &\propto \sum_j p_i p_j \text{ABF}_i \text{ABF}_j \times \delta_{ij} \kappa^{I_{i,j}} \tau_{ij} \\
&= PP_i \left(\sum_{j:I_{i,j}=0} \delta_{ij}\tau_{ij} PP_j + \kappa \sum_{j:I_{i,j}=i} \tau_{ij}\delta_{ij} PP_j \right) \\
&= PP_i \left(\sum_j \delta_{ij}\tau_{ij} PP_j + (\kappa-1) \sum_{j:I_{i,j}=i} \delta_{ij}\tau_{ij} PP_j \right) \\
&= PP_i \left(1 + (\kappa-1) \frac{\sum_{j:I_{i,j}=1} \delta_{ij}\tau_{ij} PP_j}{\sum_j \delta_{ij}\tau_{ij} PP_j} \right)
\end{aligned}$$

Rather than considering all model combinations, we reduce the model space by setting a cumulative posterior probability threshold (e.g. cpp=0.99). For each trait, we use the single-trait fine-mapping results to order the models by PP and retain those for which the sum of their PPs is below 0.99. As these δ_{ij} terms depend on the SNPs that are included in each model, a loop over the model combinations is required to make these small calculations.

1.4 Related Individuals Implementation

The above derivations are based on a sample of unrelated individuals. If the proportion of related individuals is relatively large such that their removal would be a noticeable loss in data, rather than excluding related samples, an alternative approach is considered. First, single-SNP mixed linear models that account for relatedness are fit for each trait using GEMMA[7] (Genome-wide Efficient Mixed Model Association). The output from GEMMA includes the relatedness-adjusted effect estimates $\hat{\beta}$ of each SNP for one trait, which may then be used as input to JAM[4] or FINEMAP[2], as above, to identify the models with non-negligible evidence. As the single-SNP effect estimates are adjusted for relatedness, they may be used together with the genotype matrix of unrelated samples (or reference panel) as above to obtain $\hat{\beta}$ for multi-SNP models, which are needed to get log(ABF) as in (4); as these effect estimates are adjusted for relatedness, they may be treated as if obtained from an unrelated sample. The effective sample size is used as N in the log(ABF) calculation.

Supplementary Section 2: Region construction for fine-mapping in the Ugandan cohort

In order to obtain more precision in the construction of the fine mapping regions we consider the centimorgan (cM) genetic distance between SNPs. Approximately 80% of the SNPs (hg19/build 37) in the Ugandan data set do not map to a cM (reference panel) position so missing values were imputed using linear interpolation.

We then considered the GWAS for each of the 33 traits and selected the SNPs using a p-value threshold of 1×10^{-6}.

Next, we sorted the p-values of the selected SNPs from all trait GWAS in descending order and removed any duplicated SNPs. Finally, regions were constructed using the following steps:

1. For the most significant SNP, construct a region $\pm 0.05cM$ around the SNP
2. Block correlation check: for a block of 200 SNPs on each side of the region we compute the r^2 (LD) between the 200 SNPs block and the lead SNP in the region. We extend the region until the SNP of the block with highest r^2 (considering a minimum of 0.4).
3. Repeat the procedure for subsequent SNPs, checking first if the SNP belongs to any previously constructed region.

Applying this procedure to the Ugandan data set we obtained 56 regions detailed in Supplementary Figure 5.

References

[1] Asimit JL et al. "Stochastic search and joint fine-mapping increases accuracy and identifies previously unreported associations in immune-mediated diseases". In: *Nature Communications* 10 (2019). 3216. DOI: 10.1038/s41467-019-11271-0.

[2] Benner C et al. "FINEMAP: efficient variable selection using summary data from genome-wide association studies". In: *Bioinformatics* 32.10 (2016), pp. 1493–501. DOI: 10.1093/bioinformatics/btw018.

[3] Bulik-Sullivan B et al. "LD Score regression distinguishes confounding from polygenicity in genome-wide association studies". In: *Nature Genetics* 47 (2015), pp. 291–295. DOI: 10.1038/ng.3211.

[4] Newcombe PJ et al. "JAM: A Scalable Bayesian Framework for Joint Analysis of Marginal SNP Effects". In: *Genetic Epidemiology* 40 (2016), pp. 188–201. DOI: 10.1002/gepi.21953.

[5] Wallace C et al. "Dissection of a Complex Disease Susceptibility Region Using a Bayesian Stochastic Search Approach to Fine Mapping". In: *PLOS Genetics* 11.6 (June 2015), pp. 1–22. DOI: 10.1371/journal.pgen.1005272.

[6] Wagenmakers E.-J. "A practical solution to the pervasive problems of p values". In: *Psychon. Bull. Rev.* 14 (2007), pp. 779–804. DOI: 10.1038/ng.3211.

[7] Zhou X. and Stephens M. "Genome-wide efficient mixed-model analysis for association studies". In: *Nature Genetics* 44 (2012), pp. 821–824. DOI: 10.1038/ng.2310.

Supplementary Section 3: Figures

Supplementary Figure 1: **LD (r^2) plot of *IL2RA* region, 10p-6030000-6220000.** This region was used in simulations comparing single-trait fine-mapping with flashfm and fastPAINTOR with flashfm. It is based on the CEU from 1000 Genomes Project phase 3, build GRCh37/hg19.

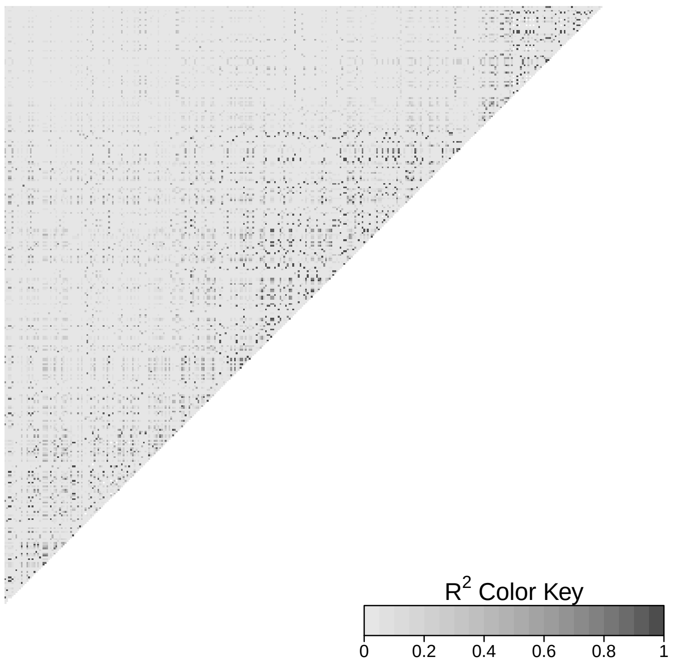

Supplementary Figure 2: **Comparison of flashfm and single trait fine-mapping when there is a shared causal variant.** Causal variants were simulated for two traits: trait 1 has causal variants A+C, while trait 2 has A+D causal variants, where $\beta_A = log(1.25)$ for both traits and $\beta_C = \beta_D = log(1.4)$. Both panels show the mean posterior probability throughout a variation of the sample size from 1000 to 5000, with no missing data. Source data are provided in Supplementary Data 1.4

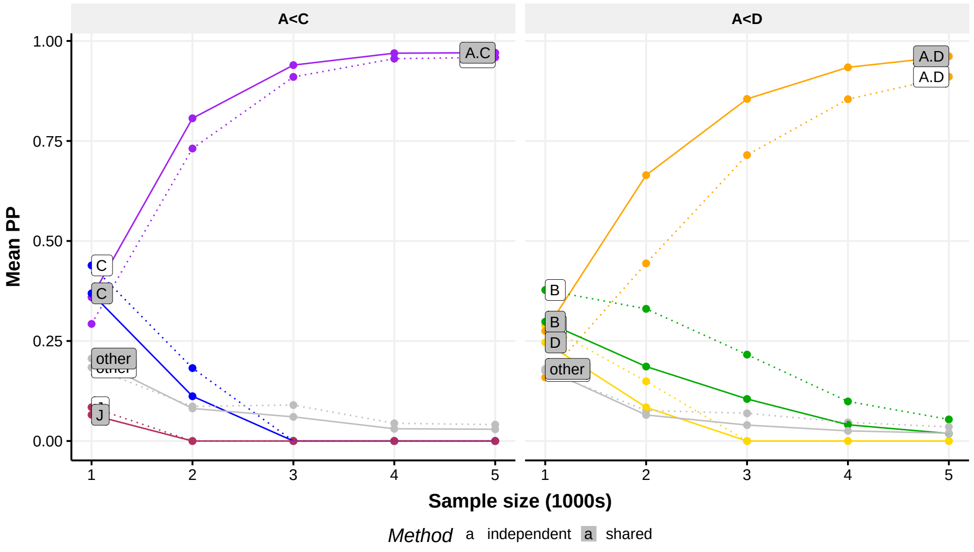

Supplementary Figure 3: **LD (r^2) plot of *CTLA4* region, 2q-204446258-204816382**. This region was used in simulations comparing fastPAINTOR with flashfm and for testing robustness of flashfm to mis-specification of trait correlation. Subsets of this region were used in assessing the running time of flashfm. It is based on the CEU from 1000 Genomes Project phase 3, build GRCh37/hg19.

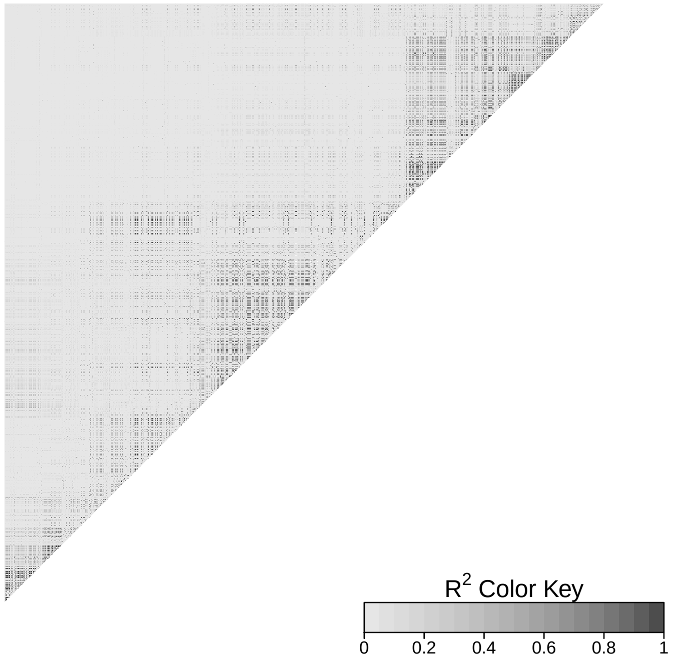

Supplementary Figure 4: **Correlation for the Ugandan cardiometabolic traits**. There are three distinct correlation blocks for anthropometric traits (height, weight, body mass index, etc.), mature red blood cell traits (mchc, mch, hct, mcv, hgb, rbc), and for lipid traits) lowdlipo, highdlipo, cholesterol and triglycerides).

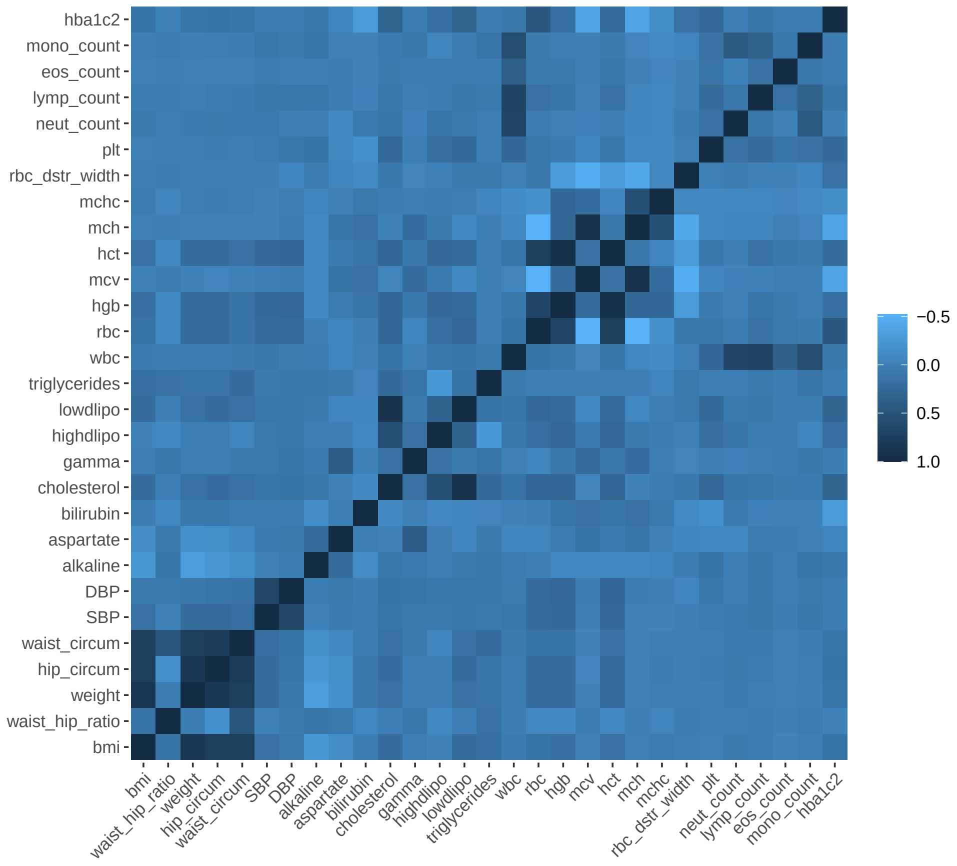

Supplementary Figure 5: **Distribution across chromosomes of number of signals per regions for cardiometabolic traits in the Ugandan data.** The legend shows the regions with 2-6 signals for the quantitative traits (QT).

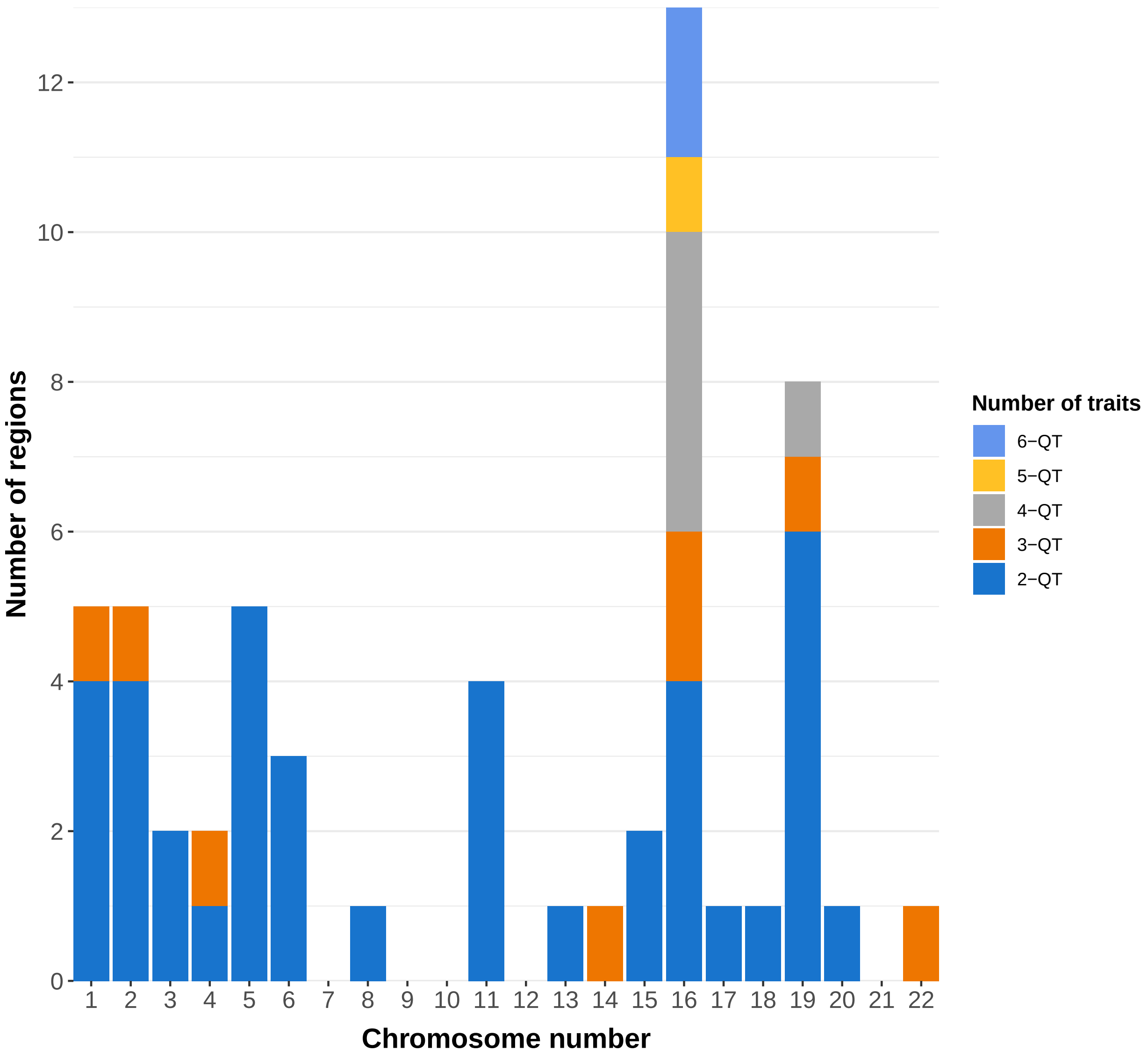

Supplement: Supplementary file 1 — Supplementary Information [file 41467_2021_26364_MOESM1_ESM.pdf]
