# Supplementary material for: The flashfm approach for fine-mapping multiple quantitative traits
Source: Nat Commun. 2021 Oct 22;12:6147. doi: 10.1038/s41467-021-26364-y (PMC8536717; doi:10.1038/s41467-021-26364-y)
Supplement: Supplementary file 3 — Description of Additional Supplementary Files [file 41467_2021_26364_MOESM3_ESM.pdf]

Description of Additional Supplementary Files

Supplementary Data 1: Details from simulations and summaries of the Ugandan cohort and its results.

Simulation details and results summaries; Ugandan cohort trait summaries, trait correlation matrix, fine mapped regions details, functional annotation of top SNPs in particular regions, top models for two single-trait approaches and coinciding flashfm results.

Supplementary Data 2: Summary of single and multi-trait finemapping results for 56 regions in the Ugandan cohort.

These regions have genetic associations for at least two of the 33 cardiometabolic traits. Both single trait fine-mapping and flashfm results are given for the top models (with model PP), as well as number of SNPs in each SNP group, and the LD between SNP groups (for both fine-mapping methods).

Supplementary Data 3: Flashfm results for all 56 regions that met criteria for fine-mapping.

For each region, SNPs from each flashfm SNP group are listed, together with base pairs position (GRCh37/hg19 assembly), alleles, and RAF in Ugandan samples.

Supplementary Data 4: FINEMAP results for all 56 regions that met criteria for fine-mapping.

For each region, SNPs from each FINEMAP SNP group are listed, together with base pairs position (GRCh37/hg19 assembly), alleles, and RAF in Ugandan samples.
