# Supplementary material for: The flashfm approach for fine-mapping multiple quantitative traits
Source: Nat Commun. 2021 Oct 22;12:6147. doi: 10.1038/s41467-021-26364-y (PMC8536717; doi:10.1038/s41467-021-26364-y)

Flashfm – Supplementary Data 2: Uganda fine-mapping results

Here we present the complete Uganda fine-mapping results from flashfm and independent fine-mapping. For all regions the independent fine-mapping method is FINEMAP, except for one region (16:302161-440165) where we used JAM; this region has 6 traits with multi-SNP models that was computationally quicker with JAM. For all the regions that involve 2 or 3 traits we consider a cumulative posterior probability (cpp) of 0.99. For regions with more than 3 traits we consider a cpp of 0.95 unless otherwise stated.

For each region we present two figures that show (i) the average LD between SNP groups constructed under single-trait fine-mapping and those constructed under flashfm; (ii) a pair of plots, each showing the average LD between SNP groups within each method. If independent fine-mapping and flashfm both favour the same single SNP model, a Venn diagram of the SNP group is presented. This illustrates how many SNPs from the flashfm group are a subset of the independent fine-mapping group.

Sometimes an independent fine-mapping group is split into two groups by flashfm. This is shown, for example by group A for independent fine-mapping and groups A.1 and A.2 from flashfm.

	Stepwise		Independent		Flashfm	
Traits	SNP/Model	P-value	Model	PP	Model	PP
lowdlipo	1:109817590/A	2.63893e-19	A	0.722	A	0.797
cholesterol	1:109817590/A	4.98465e-14	A	0.692	A	0.774

Supplementary Table 1: Fine-mapping results for 1:109811755-109825591 by independent fine-mapping and Flashfm. Stepwise results are also given.

	SNP Group
	A
Independent	2
	1:109817590
Flashfm	2
	1:109817590

Supplementary Table 2: SNP group sizes and representative variant for groups constructed by independent fine-mapping and by Flashfm for region 1:109811755-109825591.

Supplementary Figure 1: Venn diagram of SNP group constructed under indepenedent fine-mapping compared to that under flashfm for region 1:109811755-109825591. This shows that the flashfm group is a subset of, or the same as, the group form independent fine-mapping.

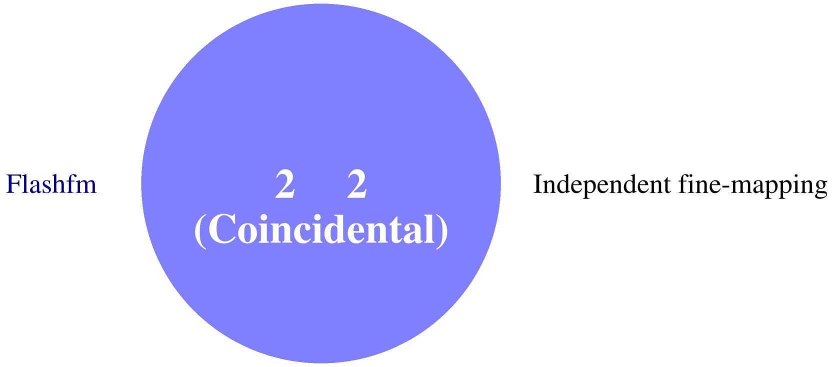

	Stepwise		Independent		Flashfm	
Traits	SNP/Model	P-value	Model	PP	Model	PP
lowdlipo	1:55520445/A	1.05319e-14	A	0.496	A	0.628
	–	–	A+C	0.092	A+C	0.061
cholesterol	1:55520445/A	2.37584e-09	A	0.639	A	0.758

Supplementary Table 3: Fine-mapping results for 1:55517883-55674945 by independent fine-mapping and Flashfm. Stepwise results are also given.

	SNP Group	
	A	C
Independent	4	1
	1:55518622	1:55522083
Flashfm	3	1
	1:55518622	1:55522083

Supplementary Table 4: SNP group sizes and representative variant for groups constructed by independent fine-mapping and by Flashfm for region 1:55517883-55674945.

Supplementary Figure 2: Average LD between SNP groups constructed under independent fine-mapping with those from flashfm for region 1:55517883-55674945.

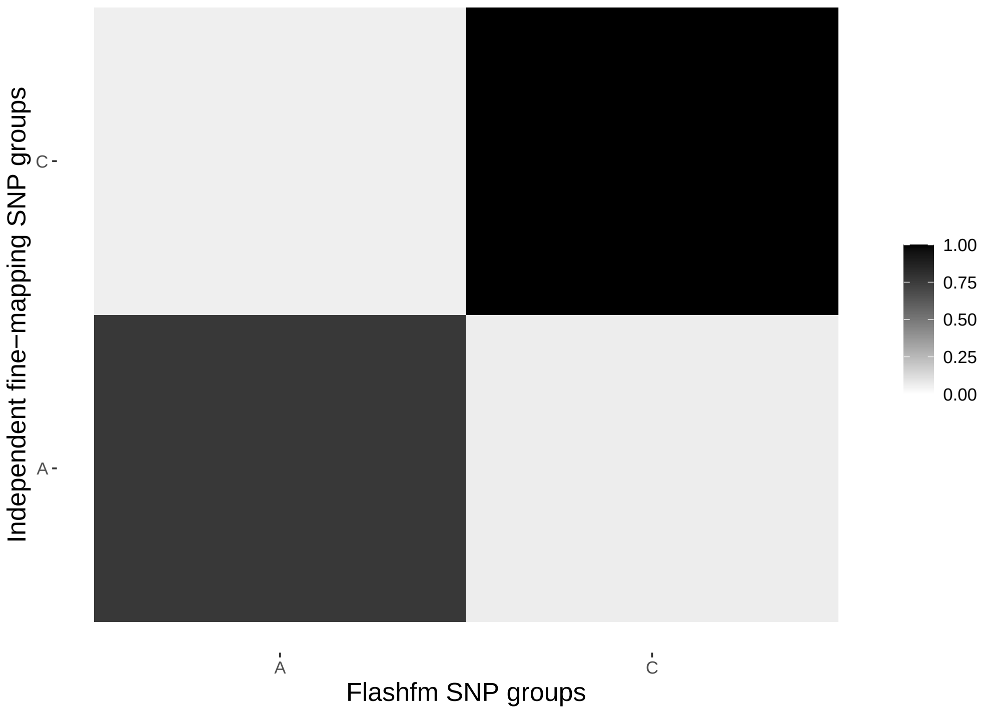

Supplementary Figure 3: Average LD between SNP groups constructed under independent fine-mapping (left) and Flashfm (right) for region 1:55517883-55674945.

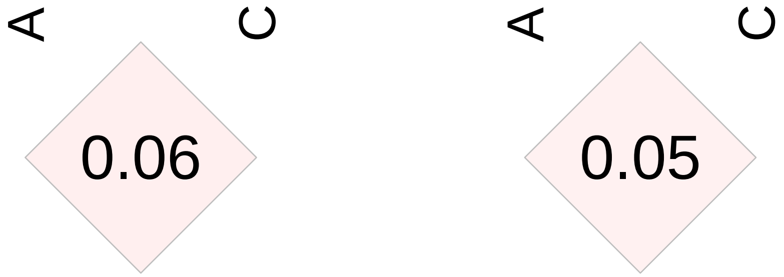

	Stepwise		Independent		Flashfm	
Traits	SNP/Model	P-value	Model	PP	Model	PP
cholesterol	1:17784317/D	4.24918e-08	D	0.279	D	0.496
	–	–	B+D	0.274	B+D	0.18
	–	–	A+D	0.085	A+D	0.069
	–	–	A+B+D	0.057	–	–
lowdlipo	1:17784317/D	5.54811e-07	B+D	0.252	B+D	0.277
	–	–	A+B+D	0.160	D	0.154
	–	–	A+D	0.068	A+B+D	0.089
	–	–	D	0.068	A+D	0.069
	–	–	B+C+D	0.051	–	–

Supplementary Table 5: Fine-mapping results for 1:17700017-17873294 by independent fine-mapping and Flashfm. Stepwise results are also given.

	SNP Group			
	A	B	C	D
Independent	29	1	14	1
	1:17730238	1:17765792	1:17843968	1:17784317
Flashfm	25	1	9	1
	1:17781513	1:17765792	1:17847630	1:17784317

Supplementary Table 6: SNP group sizes and representative variant for groups constructed by independent fine-mapping and by Flashfm for region 1:17700017-17873294.

Supplementary Figure 4: Average LD between SNP groups constructed under independent fine-mapping with those from flashfm for region 1:17700017-17873294.

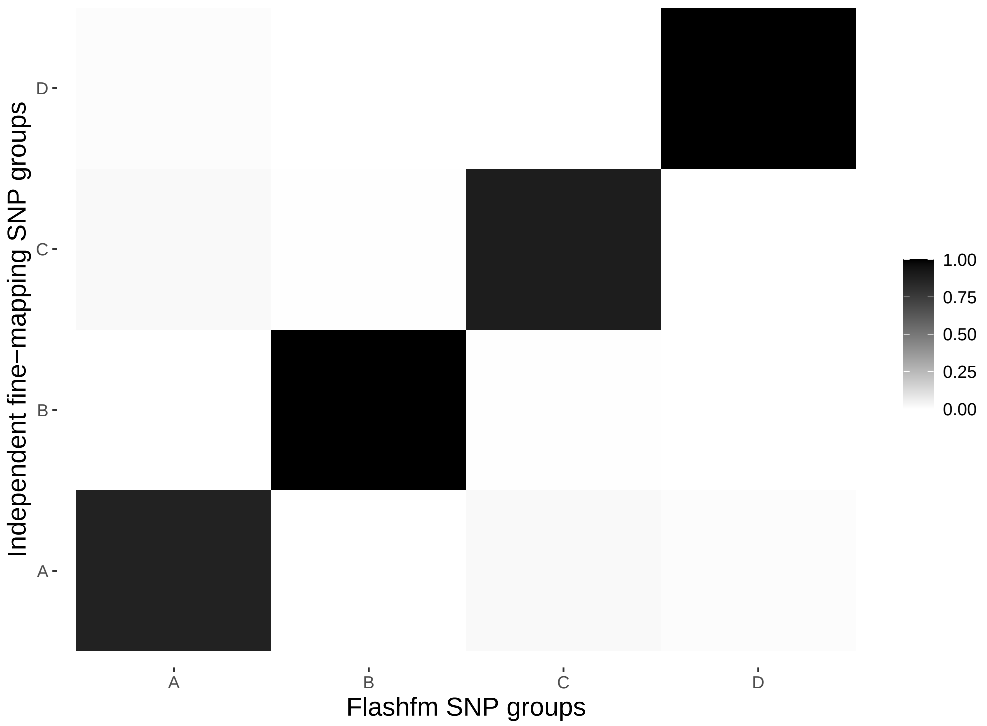

Supplementary Figure 5: Average LD between SNP groups constructed under independent fine-mapping (left) and Flashfm (right) for region 1:17700017-17873294.

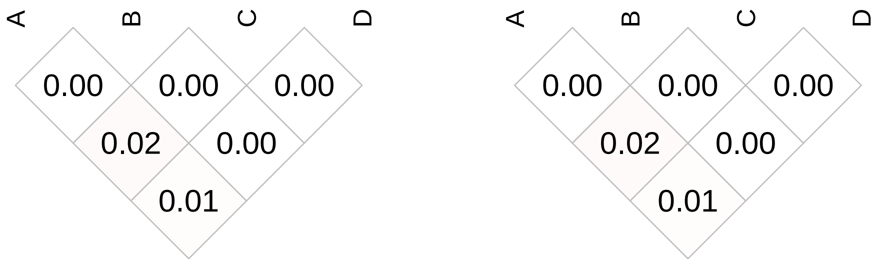

	Stepwise		Independent		Flashfm	
Traits	SNP/Model	P-value	Model	PP	Model	PP
hip_circum	1:180143575/C	8.6265e-08	C	0.463	C	0.495
	–	–	D	0.297	D	0.293
waist_circum	1:180171741/D	1.8039e-07	D	0.606	D	0.68
	–	–	C	0.086	C	0.055
weight	1:180143575/C	5.30306e-07	C	0.635	C	0.68
	–	–	A+C	0.053	–	–

Supplementary Table 7: Fine-mapping results for 1:180122985-180192543 by independent fine-mapping and Flashfm. Stepwise results are also given.

	SNP Group		
	A	C	D
Independent	9	15	2
	1:180123815	1:180187342	1:180171741
Flashfm	9	14	2
	1:180123815	1:180187342	1:180171741

Supplementary Table 8: SNP group sizes and representative variant for groups constructed by independent fine-mapping and by Flashfm for region 1:180122985-180192543.

Supplementary Figure 6: Average LD between SNP groups constructed under independent fine-mapping with those from flashfm for region 1:180122985-180192543.

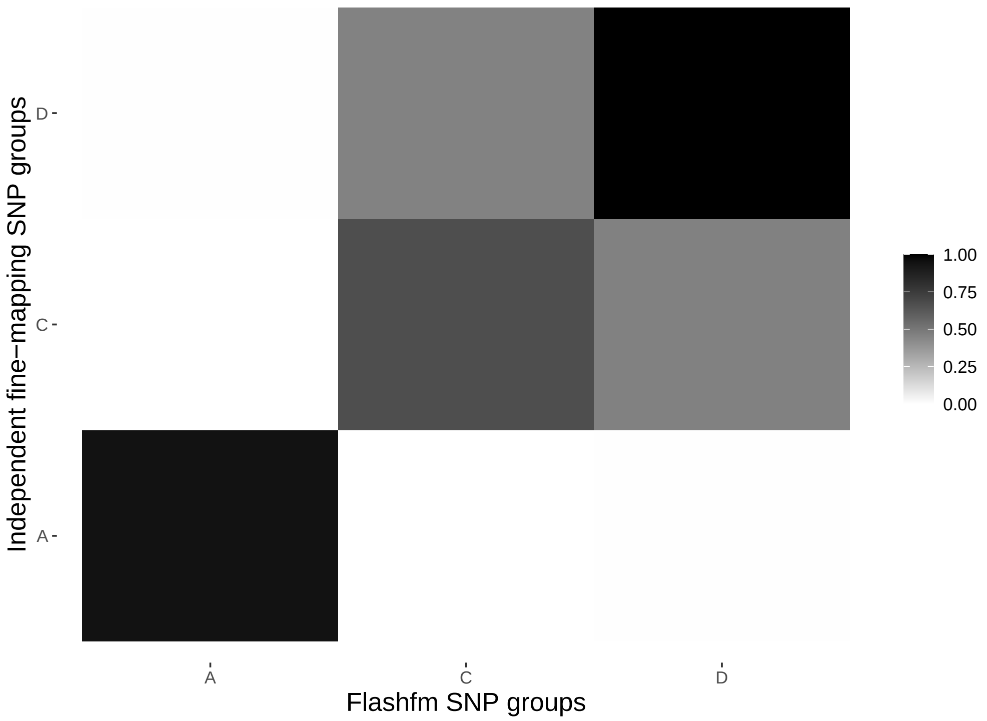

Supplementary Figure 7: Average LD between SNP groups constructed under independent fine-mapping (left) and Flashfm (right) for region 1:180122985-180192543.

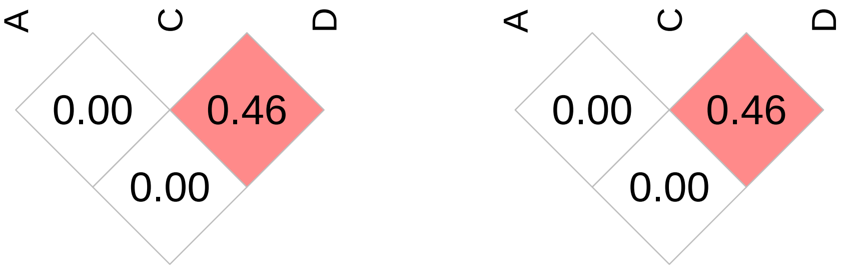

	Stepwise		Independent		Flashfm	
Traits	SNP/Model	P-value	Model	PP	Model	PP
wbc	1:220425014/B	3.13673e-07	B	0.579	B	0.505
	–	–	–	–	A	0.236
mono_count	1:220360197/A	5.88953e-07	A	0.575	A	0.655

Supplementary Table 9: Fine-mapping results for 1:220212231-220479374 by independent fine-mapping and Flashfm. Stepwise results are also given.

	SNP Group	
	A	B
Independent	1	1
	1:220360197	1:220425014
Flashfm	1	1
	1:220360197	1:220425014

Supplementary Table 10: SNP group sizes and representative variant for groups constructed by independent fine-mapping and by Flashfm for region 1:220212231-220479374.

Supplementary Figure 8: Average LD between SNP groups constructed under independent fine-mapping with those from flashfm for region 1:220212231-220479374.

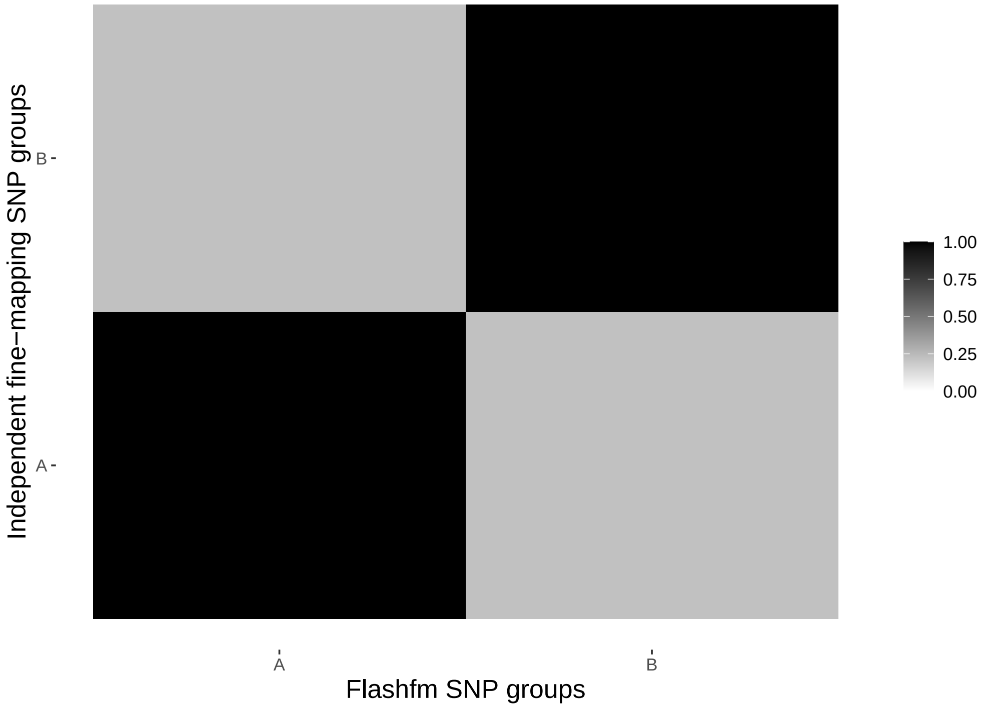

Supplementary Figure 9: Average LD between SNP groups constructed under independent fine-mapping (left) and Flashfm (right) for region 1:220212231-220479374.

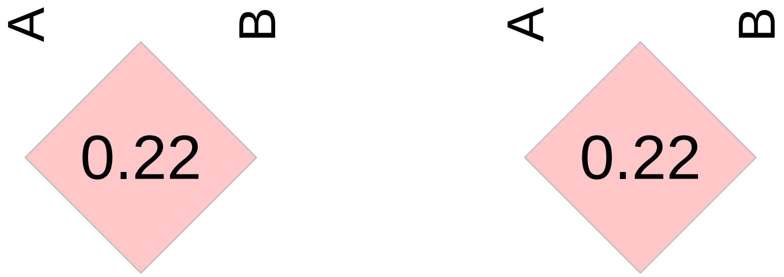

	Stepwise		Independent		Flashfm	
Traits	SNP/Model	P-value	Model	PP	Model	PP
neut_count	2:136485657/D	1.05224e-11	D	0.861	D	0.866
rbc	2:135784852/A	8.51555e-09	A+B	0.086	A+B	0.085

Supplementary Table 11: Fine-mapping results for 2:135758116-136787730 by independent fine-mapping and Flashfm. Stepwise results are also given.

	SNP Group		
	A	B	D
Independent	1	5	45
	2:135784852	2:136353460	2:136493550
Flashfm	1	5	45
	2:135784852	2:136353460	2:136493550

Supplementary Table 12: SNP group sizes and representative variant for groups constructed by independent fine-mapping and by Flashfm for region 2:135758116-136787730.

Supplementary Figure 10: Average LD between SNP groups constructed under independent fine-mapping with those from flashfm for region 2:135758116-136787730.

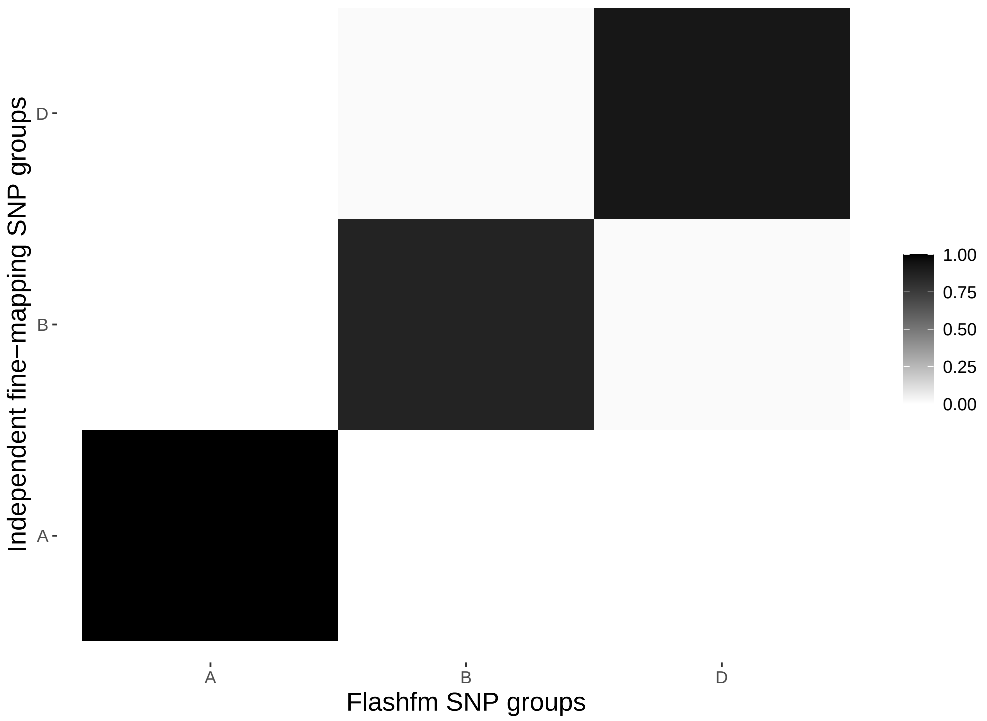

Supplementary Figure 11: Average LD between SNP groups constructed under independent fine-mapping (left) and Flashfm (right) for region 2:135758116-136787730.

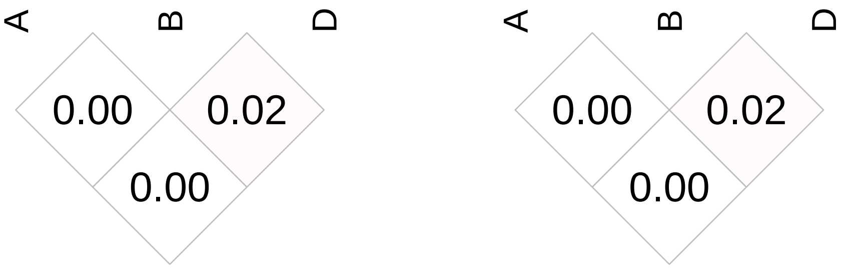

	Stepwise		Independent		Flashfm	
Traits	SNP/Model	P-value	Model	PP	Model	PP
hba1c2	2:189377509/I	6.51918e-10	A+B+G+H+I+K	0.622	A+B+G+H+I+K	0.595
	2:189593139/G	8.20728e-07	A+B+G+H+K	0.214	A+B+G+H+K	0.230
	–	–	–	–	A+B+G+H	0.050
weight	2:189382844/D	2.28506e-07	D	0.536	D	0.436
	–	–	–	–	J	0.131
	–	–	–	–	D+J	0.124
gamma	2:189185755/J	3.11663e-07	J	0.893	J	0.672
	–	–	–	–	B+J	0.187

Supplementary Table 13: Fine-mapping results for 2:189071956-189635041 by independent fine-mapping and Flashfm. Stepwise results are also given.

	SNP Group					
	A	B	D	G	H	I
Independent	2<br>2:189436024	1<br>2:189461890	5<br>2:189350336	1<br>2:189593139	17<br>2:189629716	5<br>2:189377509
Flashfm	2<br>2:189436024	1<br>2:189461890	5<br>2:189350336	1<br>2:189593139	17<br>2:189629716	4<br>2:189413894
	J	K				
Independent	20<br>2:189194029	15<br>2:189443782				
Flashfm	17<br>2:189106677	15<br>2:189443782				

Supplementary Table 14: SNP group sizes and representative variant for groups constructed by independent fine-mapping and by Flashfm for region 2:189071956-189635041.

Supplementary Figure 12: Average LD between SNP groups constructed under independent fine-mapping with those from flashfm for region 2:189071956-189635041.

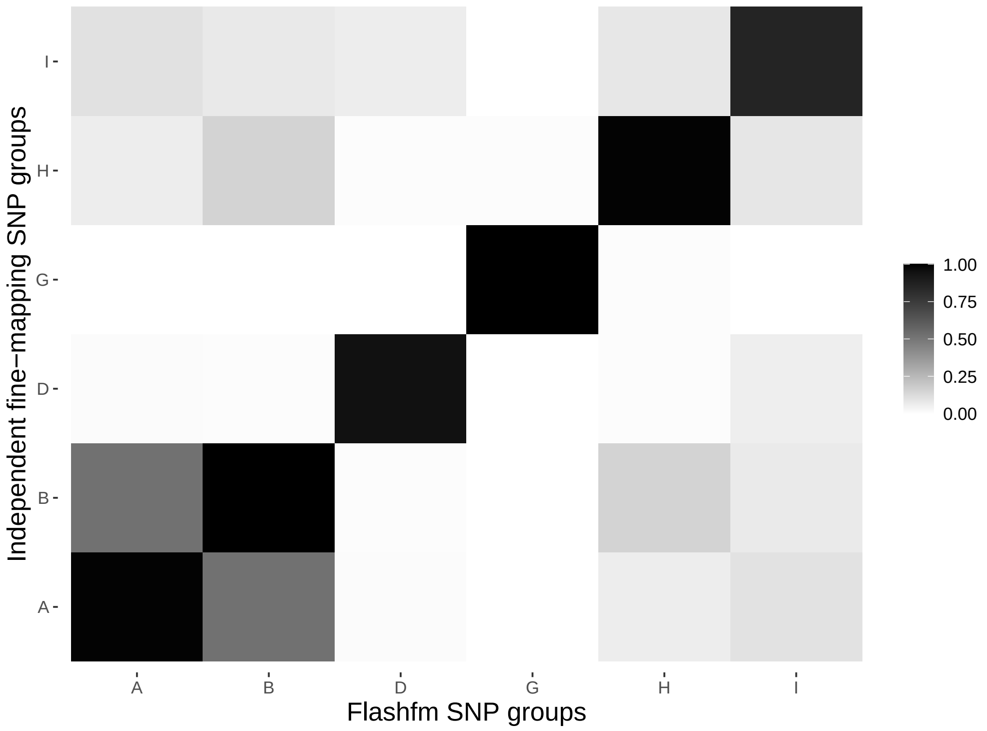

Supplementary Figure 13: Average LD between SNP groups constructed under independent fine-mapping (left) and Flashfm (right) for region 2:189071956-189635041.

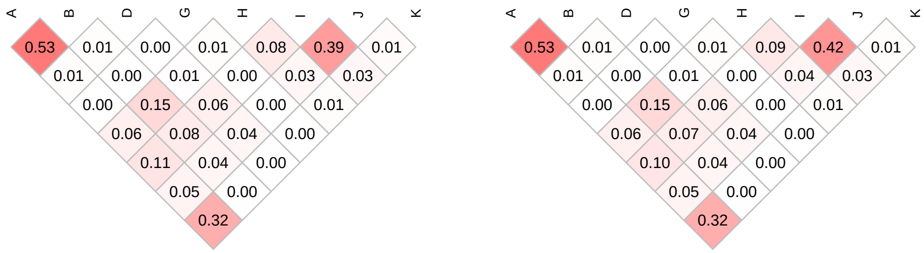

	Stepwise		Independent		Flashfm	
Traits	SNP/Model	P-value	Model	PP	Model	PP
alkaline	2:62760476/B	5.9724e-08	B	0.657	B	0.731
plt	2:62742534/J	4.9984e-07	J	0.462	B+J	0.623
	–	–	–	–	J	0.156
	–	–	–	–	B	0.078

Supplementary Table 15: Fine-mapping results for 2:62716187-62887884 by independent fine-mapping and Flashfm. Stepwise results are also given.

	SNP Group	
	B	J
Independent	8	1
	2:62781499	2:62742534
Flashfm	5	1
	2:62761855	2:62742534

Supplementary Table 16: SNP group sizes and representative variant for groups constructed by independent fine-mapping and by Flashfm for region 2:62716187-62887884.

Supplementary Figure 14: Average LD between SNP groups constructed under independent fine-mapping with those from flashfm for region 2:62716187-62887884.

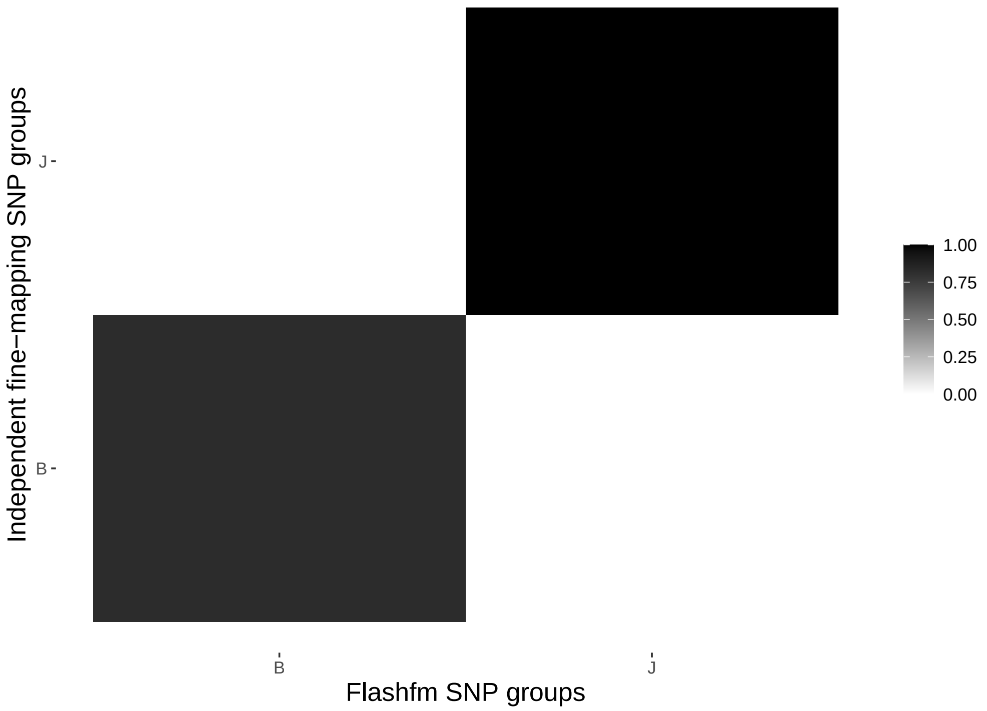

Supplementary Figure 15: Average LD between SNP groups constructed under independent fine-mapping (left) and Flashfm (right) for region 2:62716187-62887884.

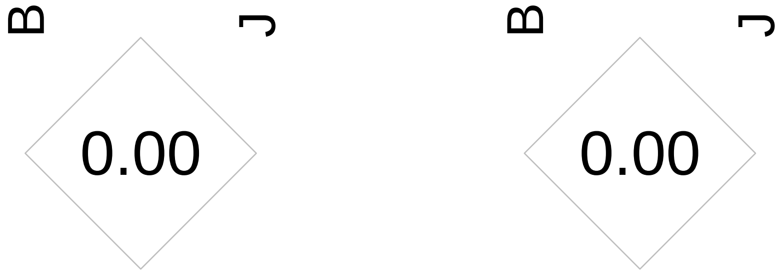

	Stepwise		Independent		Flashfm	
Traits	SNP/Model	P-value	Model	PP	Model	PP
aspartate	2:85387799/F	2.62681e-07	F	0.66	F	0.676
	–	–	–	–	E+F	0.058
rbc_dstr_width	2:85382730/G	7.46467e-07	G	0.318	F+G	0.325
	–	–	E	0.231	G	0.184
	–	–	A+G	0.119	E	0.142
	–	–	A+E	0.058	E+F	0.113
	–	–	–	–	A+G	0.054

Supplementary Table 17: Fine-mapping results for 2:85364419-85412152 by independent fine-mapping and Flashfm. Stepwise results are also given.

	SNP Group			
	A	E	F	G
Independent	13	21	24	2
	2:85400920	2:85379656	2:85410632	2:85382730
Flashfm	13	21	13	2
	2:85400920	2:85379656	2:85387799	2:85382730

Supplementary Table 18: SNP group sizes and representative variant for groups constructed by independent fine-mapping and by Flashfm for region 2:85364419-85412152.

Supplementary Figure 16: Average LD between SNP groups constructed under independent fine-mapping with those from flashfm for region 2:85364419-85412152.

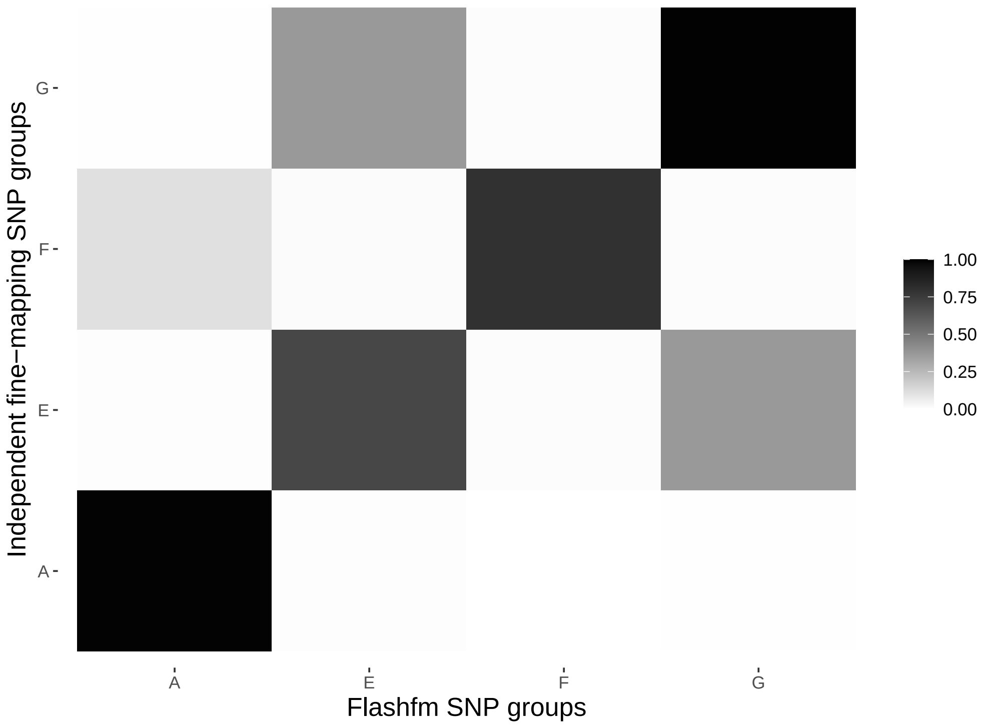

Supplementary Figure 17: Average LD between SNP groups constructed under independent fine-mapping (left) and Flashfm (right) for region 2:85364419-85412152.

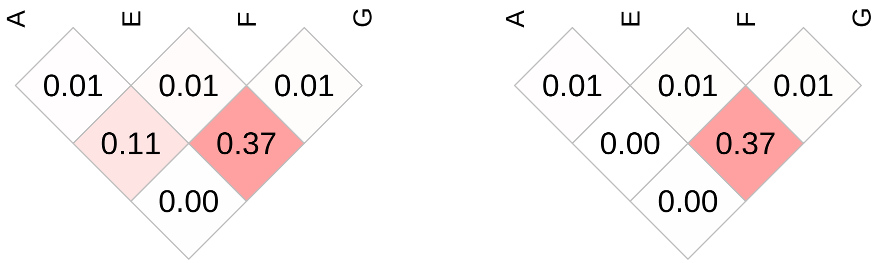

	Stepwise		Independent		Flashfm	
Traits	SNP/Model	P-value	Model	PP	Model	PP
hct	2:18682721/B	6.1164e-07	B	0.547	B	0.511
	–	–	B+C	0.085	A	0.133
	–	–	A	0.075	B+C	0.097
hgb	2:18682721/B	7.50734e-07	B	0.677	B	0.65
	–	–	A	0.068	A	0.127
	–	–	B+F	0.055	–	–

Supplementary Table 19: Fine-mapping results for 2:18651577-18703005 by independent fine-mapping and Flashfm. Stepwise results are also given.

	SNP Group			
	A	B	C	F
Independent	5	11	2	13
	2:18703005	2:18675213	2:18701221	2:18691613
Flashfm	5	11	2	13
	2:18703005	2:18675213	2:18701221	2:18691613

Supplementary Table 20: SNP group sizes and representative variant for groups constructed by independent fine-mapping and by Flashfm for region 2:18651577-18703005.

Supplementary Figure 18: Average LD between SNP groups constructed under independent fine-mapping with those from flashfm for region 2:18651577-18703005.

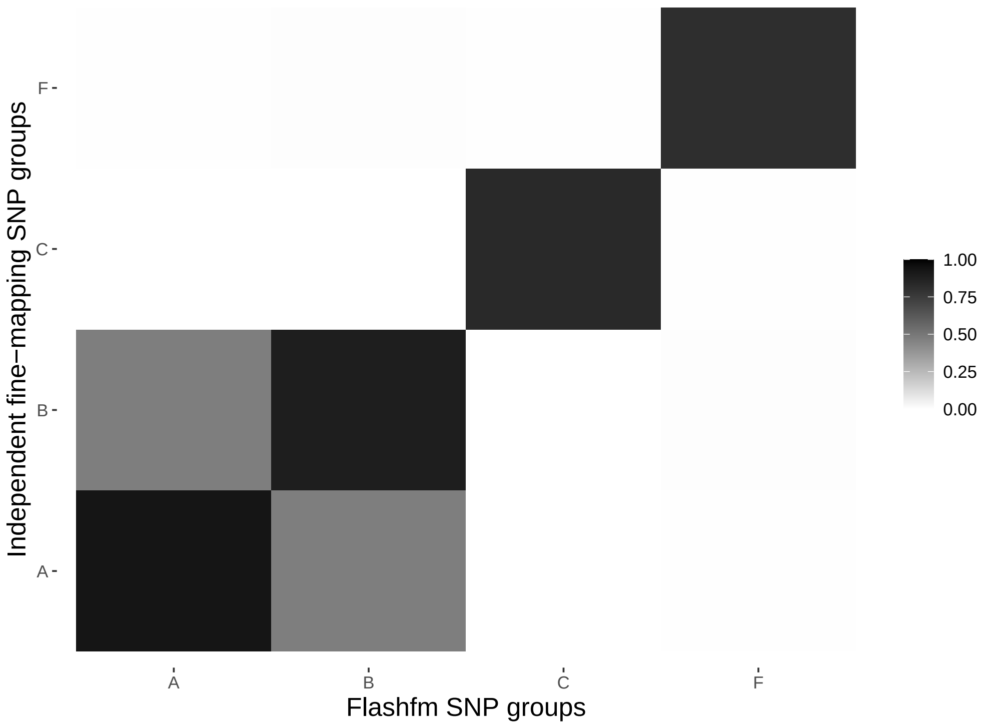

Supplementary Figure 19: Average LD between SNP groups constructed under independent fine-mapping (left) and Flashfm (right) for region 2:18651577-18703005.

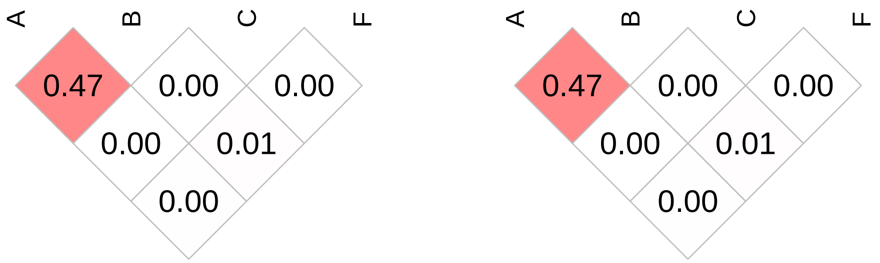

	Stepwise		Independent		Flashfm	
Traits	SNP/Model	P-value	Model	PP	Model	PP
hgb	3:48547711/A	1.02557e-07	B	0.613	B.1	0.623
	–	–	A	0.222	A	0.266
hct	3:48547711/A	1.81636e-07	A	0.468	A	0.550
	–	–	B	0.323	B.1	0.290

Supplementary Table 21: Fine-mapping results for 3:48378500-48938297 by independent fine-mapping and Flashfm. Stepwise results are also given.

	SNP Group		
	A	B	B.1
Independent	8	40	0
	3:48721571	3:48626557	–
Flashfm	8	0	29
	3:48721571	–	3:48626557

Supplementary Table 22: SNP group sizes and representative variant for groups constructed by independent fine-mapping and by Flashfm for region 3:48378500-48938297.

Supplementary Figure 20: Average LD between SNP groups constructed under independent fine-mapping with those from flashfm for region 3:48378500-48938297.

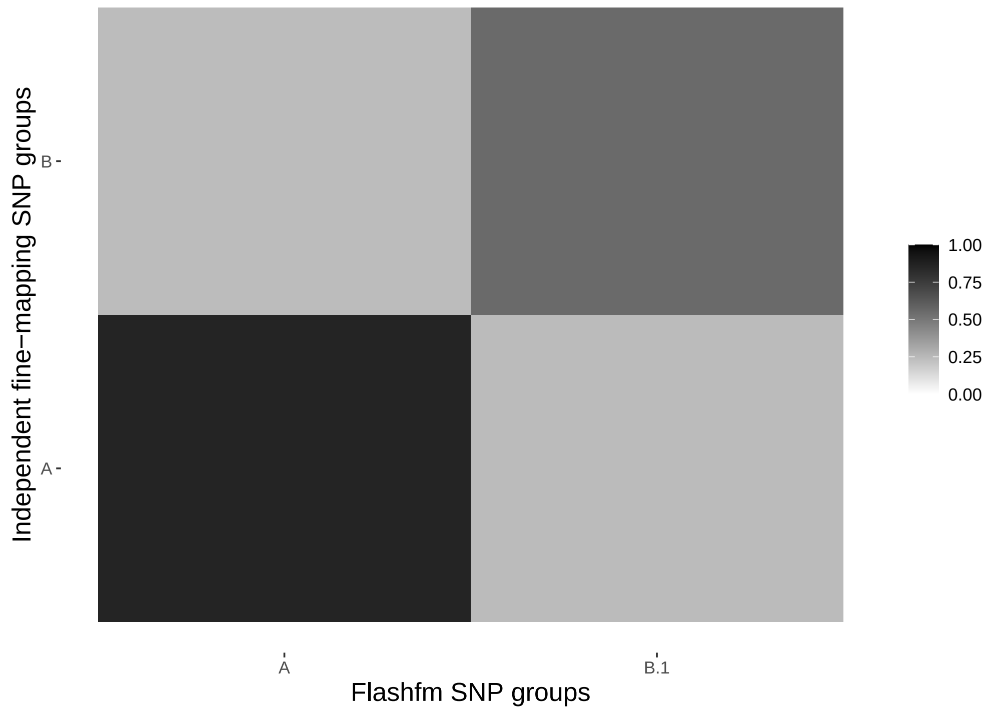

Supplementary Figure 21: Average LD between SNP groups constructed under independent fine-mapping (left) and Flashfm (right) for region 3:48378500-48938297.

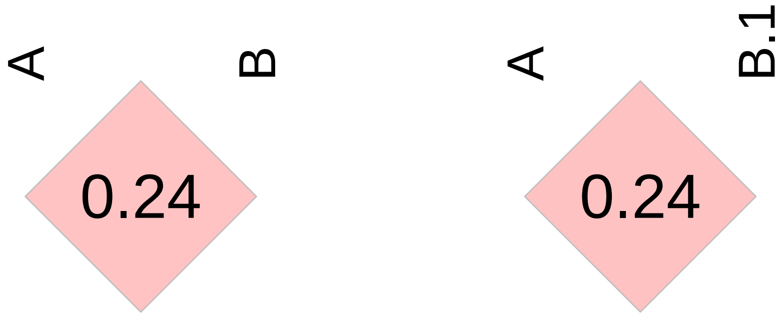

	Stepwise		Independent		Flashfm	
Traits	SNP/Model	P-value	Model	PP	Model	PP
SBP	3:135663939/A	4.25088e-07	A	0.861	A.2	0.762
	–	–	A+C	0.054	A.1	0.165
DBP	3:135673880/A	6.06157e-07	A+B	0.643	A.2+B	0.555
	–	–	A	0.335	A.2	0.433

Supplementary Table 23: Fine-mapping results for 3:135548414-135986942 by independent fine-mapping and Flashfm. Stepwise results are also given.

	SNP Group				
	A	B	C	A.1	A.2
Independent	98	54	21	0	0
	3:135650060	3:135589895	3:135692426	–	–
Flashfm	0	54	14	2	36
	–	3:135589895	3:135658784	3:135650060	3:135676132

Supplementary Table 24: SNP group sizes and representative variant for groups constructed by independent fine-mapping and by Flashfm for region 3:135548414-135986942.

Supplementary Figure 22: Average LD between SNP groups constructed under independent fine-mapping with those from flashfm for region 3:135548414-135986942.

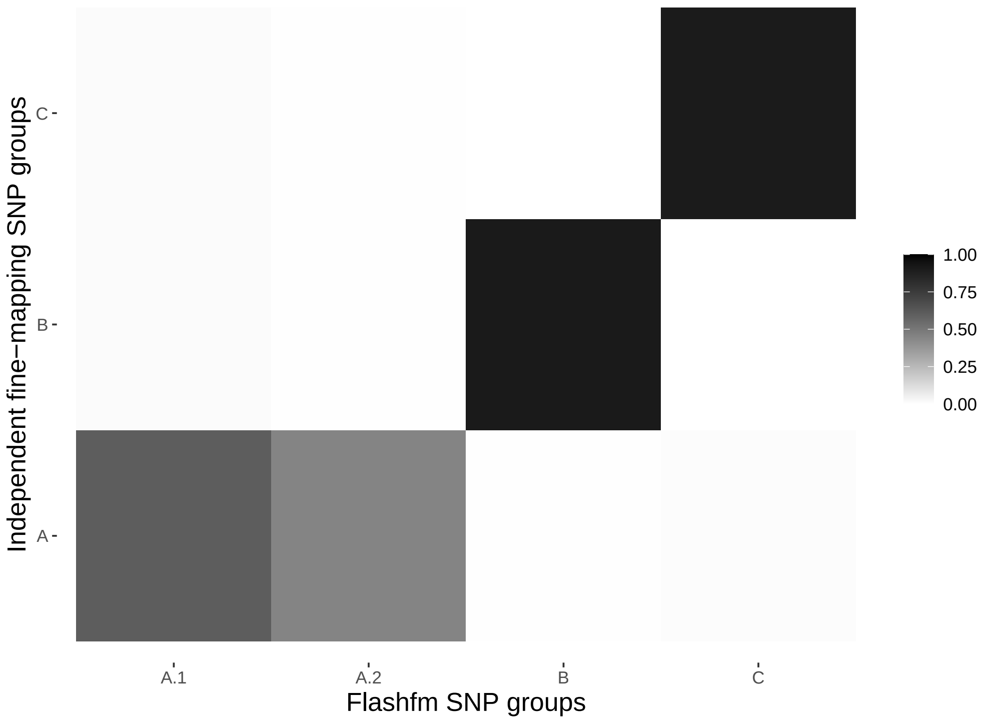

Supplementary Figure 23: Average LD between SNP groups constructed under independent fine-mapping (left) and Flashfm (right) for region 3:135548414-135986942.

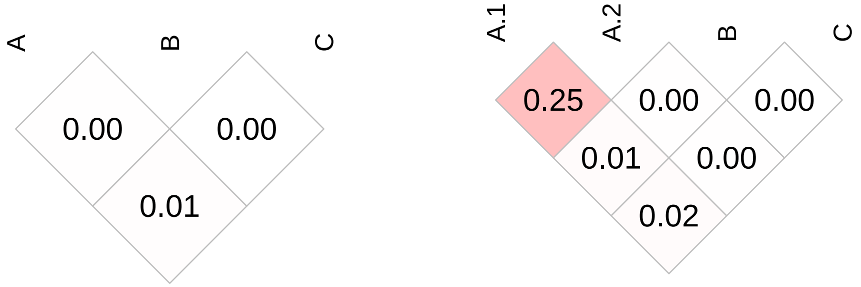

	Stepwise		Independent		Flashfm	
Traits	SNP/Model	P-value	Model	PP	Model	PP
lymp_count	4:53095312/B	5.56795e-08	B	0.789	B	0.832
weight	4:53083377/A	4.82996e-07	A	0.665	A	0.718

Supplementary Table 25: Fine-mapping results for 4:49648062-53304218 by independent fine-mapping and Flashfm. Stepwise results are also given.

	SNP Group	
	A	B
Independent	1	2
	4:53083377	4:53060664
Flashfm	1	2
	4:53083377	4:53060664

Supplementary Table 26: SNP group sizes and representative variant for groups constructed by independent fine-mapping and by Flashfm for region 4:49648062-53304218.

Supplementary Figure 24: Average LD between SNP groups constructed under independent fine-mapping with those from flashfm for region 4:49648062-53304218.

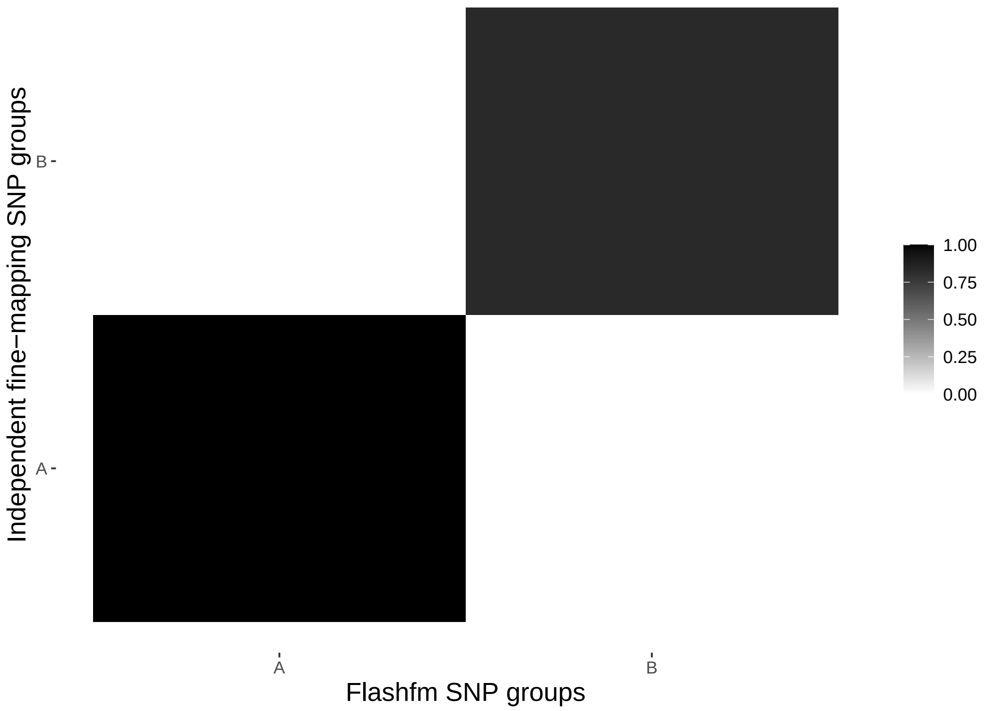

Supplementary Figure 25: Average LD between SNP groups constructed under independent fine-mapping (left) and Flashfm (right) for region 4:49648062-53304218.

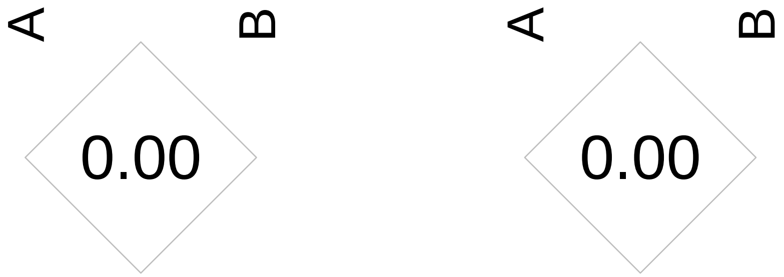

	Stepwise		Independent		Flashfm	
Traits	SNP/Model	P-value	Model	PP	Model	PP
lymp_count	4:19765235/G	7.2126e-08	G	0.403	G	0.49
	–	–	E+G	0.213	E+G	0.184
	–	–	D+G	0.063	D+G	0.056
wbc	4:19765235/G	1.62137e-07	G	0.714	G	0.794
mono_count	4:19765235/G	7.48651e-07	G	0.369	G	0.532
	–	–	C+G	0.088	C+G	0.092
	–	–	C	0.061	E+G	0.055
	–	–	E+G	0.052	–	–

Supplementary Table 27: Fine-mapping results for 4:19443144-19852689 by independent fine-mapping and Flashfm. Stepwise results are also given.

	SNP Group			
	C	D	E	G
Independent	23	4	59	1
	4:19598292	4:19532959	4:19638561	4:19765235
Flashfm	23	4	52	1
	4:19598292	4:19532959	4:19638561	4:19765235

Supplementary Table 28: SNP group sizes and representative variant for groups constructed by independent fine-mapping and by Flashfm for region 4:19443144-19852689.

Supplementary Figure 26: Average LD between SNP groups constructed under independent fine-mapping with those from flashfm for region 4:19443144-19852689.

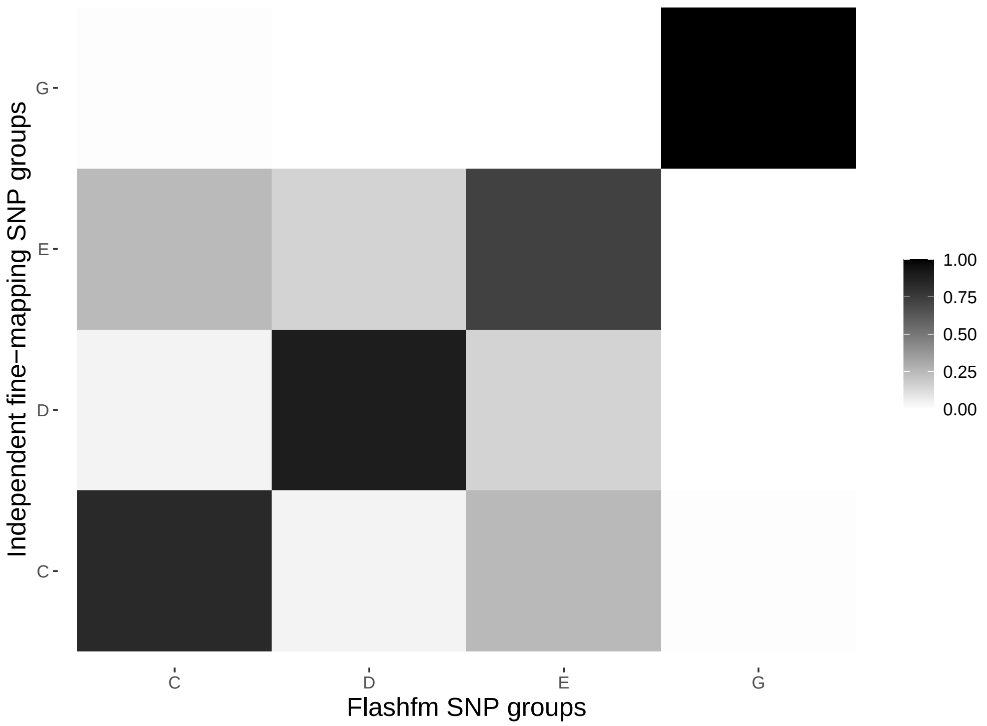

Supplementary Figure 27: Average LD between SNP groups constructed under independent fine-mapping (left) and Flashfm (right) for region 4:19443144-19852689.

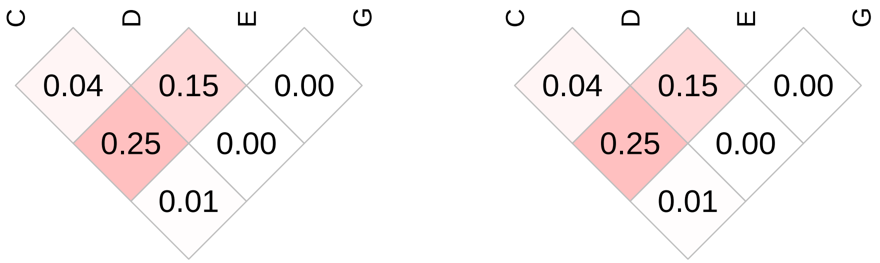

	Stepwise		Independent		Flashfm	
Traits	SNP/Model	P-value	Model	PP	Model	PP
cholesterol	5:156378584/A	4.0245e-10	A	0.328	A	0.494
	–	–	A+G	0.288	A+G	0.211
	–	–	A+E	0.177	A+E	0.129
lowdlipo	5:156309938/A	2.93049e-09	A+G	0.491	A+G	0.445
	–	–	A	0.136	A	0.253
	–	–	A+E	0.094	A+E	0.085

Supplementary Table 29: Fine-mapping results for 5:156281094-156517240 by independent fine-mapping and Flashfm. Stepwise results are also given.

	SNP Group		
	A	E	G
Independent	5	37	47
	5:156309938	5:156482884	5:156443079
Flashfm	5	37	47
	5:156309938	5:156482884	5:156443079

Supplementary Table 30: SNP group sizes and representative variant for groups constructed by independent fine-mapping and by Flashfm for region 5:156281094-156517240.

Supplementary Figure 28: Average LD between SNP groups constructed under independent fine-mapping with those from flashfm for region 5:156281094-156517240.

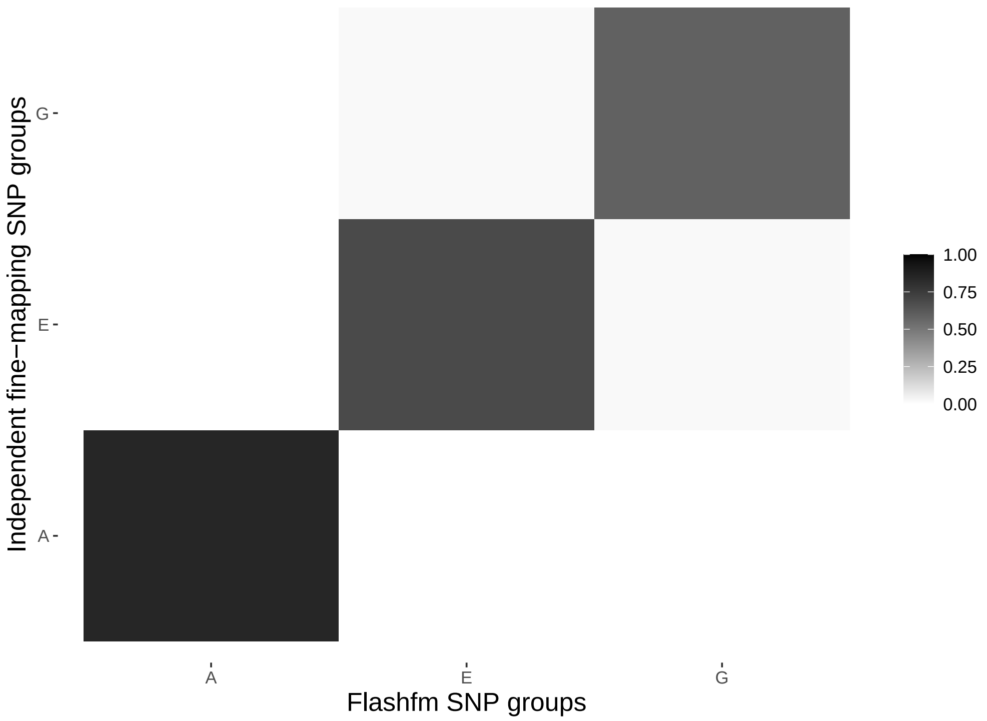

Supplementary Figure 29: Average LD between SNP groups constructed under independent fine-mapping (left) and Flashfm (right) for region 5:156281094-156517240.

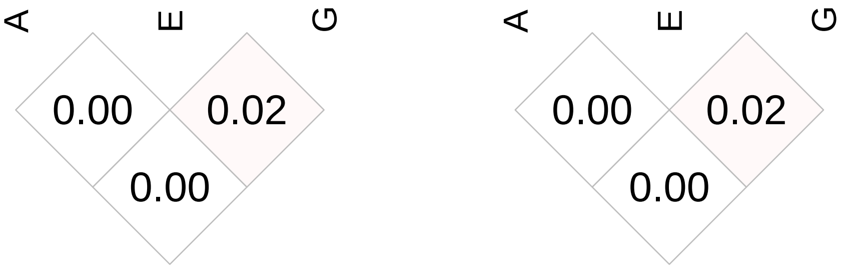

	Stepwise		Independent		Flashfm	
Traits	SNP/Model	P-value	Model	PP	Model	PP
cholesterol	5:156309938/B	1.46167e-09	B	0.684	B	0.795
	–	–	B+E	0.051	–	–
lowdlipo	5:156309938/B	2.93049e-09	B	0.436	B	0.601
	–	–	B+E	0.157	B+E	0.106
	–	–	B+F	0.062	–	–

Supplementary Table 31: Fine-mapping results for 5:156067961-156345403 by independent fine-mapping and Flashfm. Stepwise results are also given.

	SNP Group		
	B	E	F
Independent	4	27	20
	5:156142225	5:156294885	5:156226108
Flashfm	4	27	17
	5:156142225	5:156294885	5:156257190

Supplementary Table 32: SNP group sizes and representative variant for groups constructed by independent fine-mapping and by Flashfm for region 5:156067961-156345403.

Supplementary Figure 30: Average LD between SNP groups constructed under independent fine-mapping with those from flashfm for region 5:156067961-156345403.

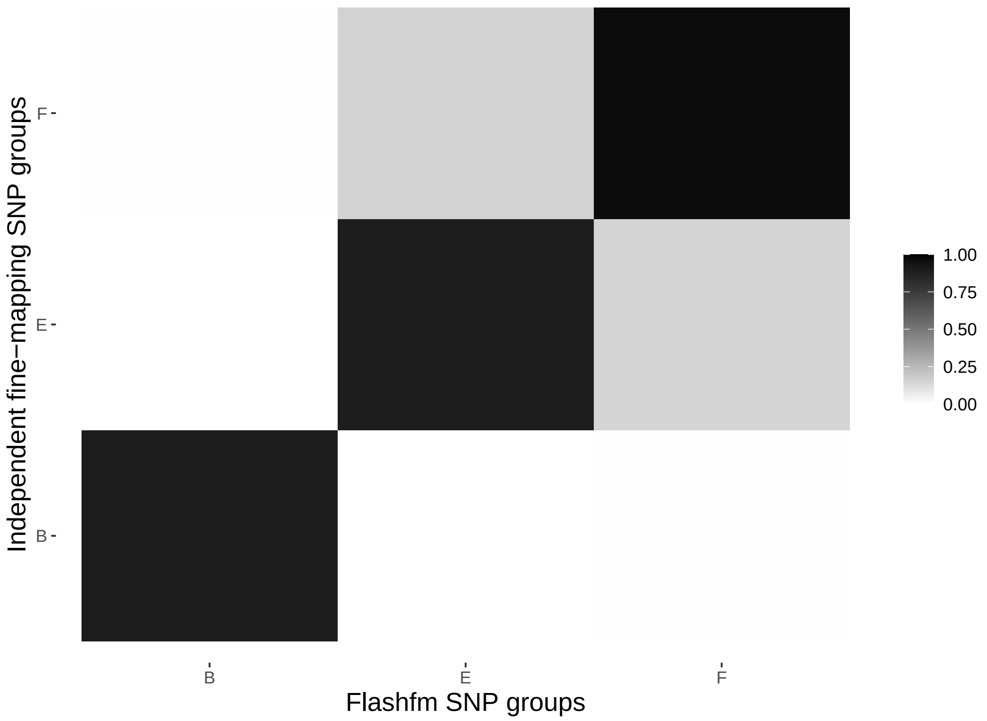

Supplementary Figure 31: Average LD between SNP groups constructed under independent fine-mapping (left) and Flashfm (right) for region 5:156067961-156345403.

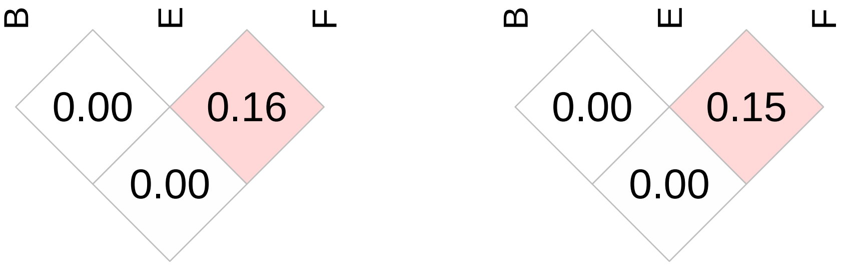

	Stepwise		Independent		Flashfm	
Traits	SNP/Model	P-value	Model	PP	Model	PP
cholesterol	5:155883245/C	1.08914e-08	C	0.677	C	0.521
	–	–	A	0.111	A	0.307
lowdlipo	5:155883245/C	4.42672e-08	C	0.732	C	0.677
	–	–	–	–	B	0.081

Supplementary Table 33: Fine-mapping results for 5:155765598-155972507 by independent fine-mapping and Flashfm. Stepwise results are also given.

	SNP Group		
	A	B	C
Independent	1	1	5
	5:155960873	5:155972507	5:155916168
Flashfm	1	1	5
	5:155960873	5:155972507	5:155916168

Supplementary Table 34: SNP group sizes and representative variant for groups constructed by independent fine-mapping and by Flashfm for region 5:155765598-155972507.

Supplementary Figure 32: Average LD between SNP groups constructed under independent fine-mapping with those from flashfm for region 5:155765598-155972507.

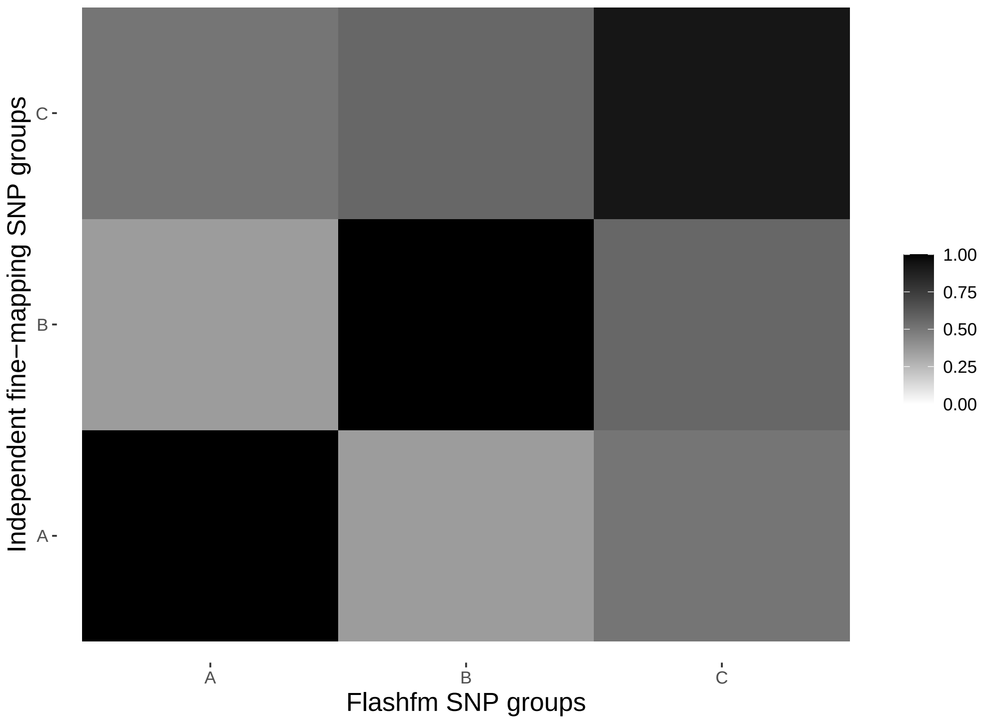

Supplementary Figure 33: Average LD between SNP groups constructed under independent fine-mapping (left) and Flashfm (right) for region 5:155765598-155972507.

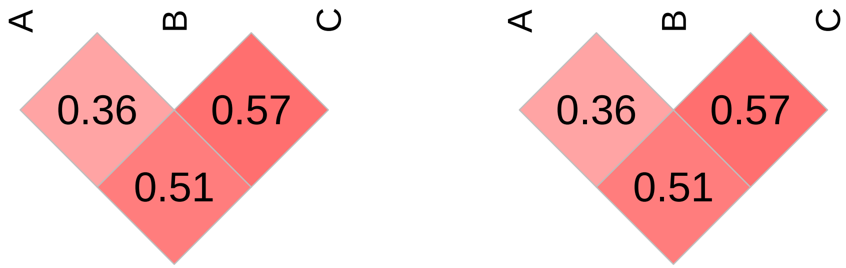

	Stepwise		Independent		Flashfm	
Traits	SNP/Model	P-value	Model	PP	Model	PP
hct	5:133872879/A	2.41027e-08	A	0.551	A	0.657
	–	–	A+C	0.078	A+C	0.051
hgb	5:133881621/A	1.31749e-07	A	0.574	A	0.676

Supplementary Table 35: Fine-mapping results for 5:133815324-133907415 by independent fine-mapping and Flashfm. Stepwise results are also given.

	SNP Group	
	A	C
Independent	3	8
	5:133860151	5:133836607
Flashfm	3	8
	5:133860151	5:133836607

Supplementary Table 36: SNP group sizes and representative variant for groups constructed by independent fine-mapping and by Flashfm for region 5:133815324-133907415.

Supplementary Figure 34: Average LD between SNP groups constructed under independent fine-mapping with those from flashfm for region 5:133815324-133907415.

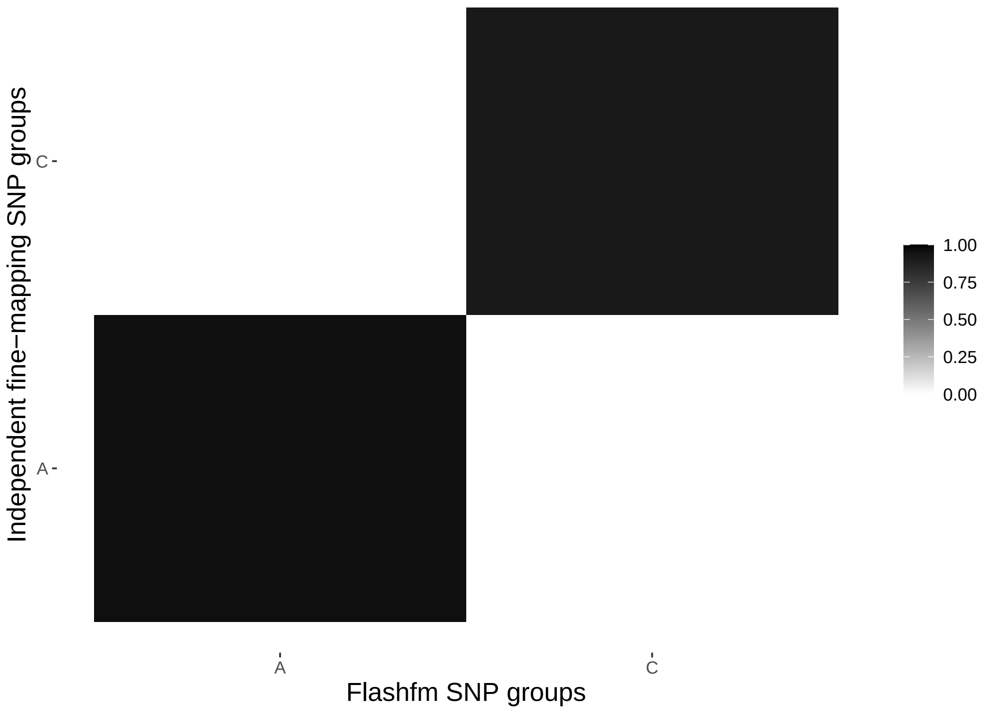

Supplementary Figure 35: Average LD between SNP groups constructed under independent fine-mapping (left) and Flashfm (right) for region 5:133815324-133907415.

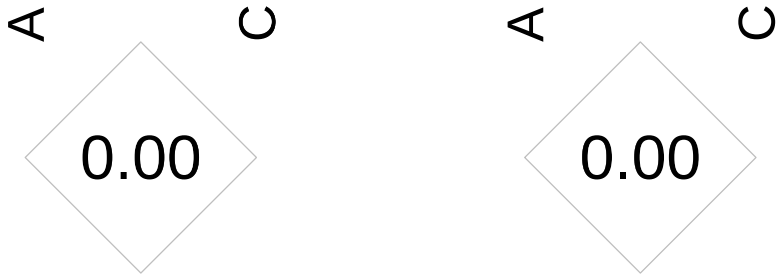

	Stepwise		Independent		Flashfm	
Traits	SNP/Model	P-value	Model	PP	Model	PP
cholesterol	5:155781587/B	5.89497e-08	B	0.851	B	0.837
lowdlipo	5:155781587/B	5.3179e-08	B	0.816	B	0.801

Supplementary Table 37: Fine-mapping results for 5:155573180-155781587 by independent fine-mapping and Flashfm. Stepwise results are also given.

	SNP Group
	B
Independent	11
	5:155781587
Flashfm	11
	5:155781587

Supplementary Table 38: SNP group sizes and representative variant for groups constructed by independent fine-mapping and by Flashfm for region 5:155573180-155781587.

Supplementary Figure 36: Venn diagram of SNP group constructed under indepenedent fine-mapping compared to that under flashfm for region 5:155573180-155781587. This shows that the flashfm group is a subset of, or the same as, the group form independent fine-mapping.

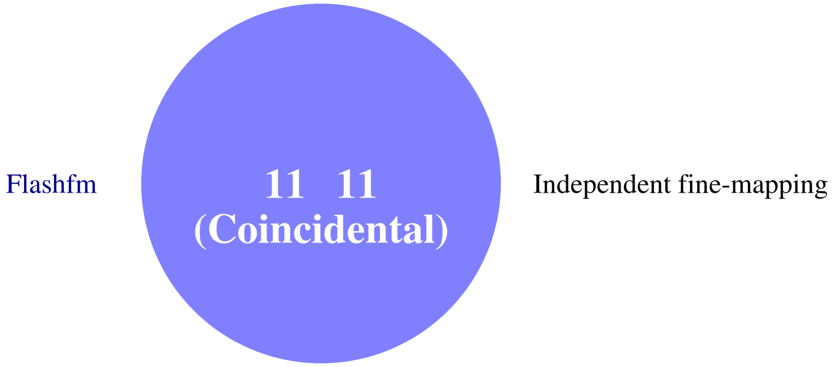

	Stepwise		Independent		Flashfm	
Traits	SNP/Model	P-value	Model	PP	Model	PP
hba1c2	6:32609126/B	4.1838e-08	B+C+I	0.34	B+C+I+K	0.648
	6:32601902/C	7.53393e-07	B+C	0.182	B+C+K	0.147
	6:32665913/I	1.38104e-07	B	0.113	B+C+I	0.059
	–	–	–	–	B+C	0.055
aspartate	6:32583830/chr6_32583830	1.46603e-07	A+D+E+F+K+L	0.516	A+C+D+E+G+K	0.447
	–	–	A+D+E+F+K	0.128	A+D+E+F+K+L	0.215
	–	–	–	–	A+D+E+F+K	0.082

Supplementary Table 39: Fine-mapping results for 6:32559710-32671044 by independent fine-mapping and Flashfm. Stepwise results are also given.

	SNP Group						
	A	B	C	D	E	F	G
Independent	21	17	1	1	1	18	1
	6:32570281	6:32650409	6:32601902	6:32561422	6:32573910	6:32667458	6:32597435
Flashfm	18	12	1	1	1	11	1
	6:32568522	6:32628306	6:32601902	6:32561422	6:32573910	6:32670145	6:32597435
	I	K	L				
Independent	1	1	1				
	6:32665913	6:32579904	6:32568521				
Flashfm	1	1	1				
	6:32665913	6:32579904	6:32568521				

Supplementary Table 40: SNP group sizes and representative variant for groups constructed by independent fine-mapping and by Flashfm for region 6:32559710-32671044.

Supplementary Figure 37: Average LD between SNP groups constructed under independent fine-mapping with those from flashfm for region 6:32559710-32671044.

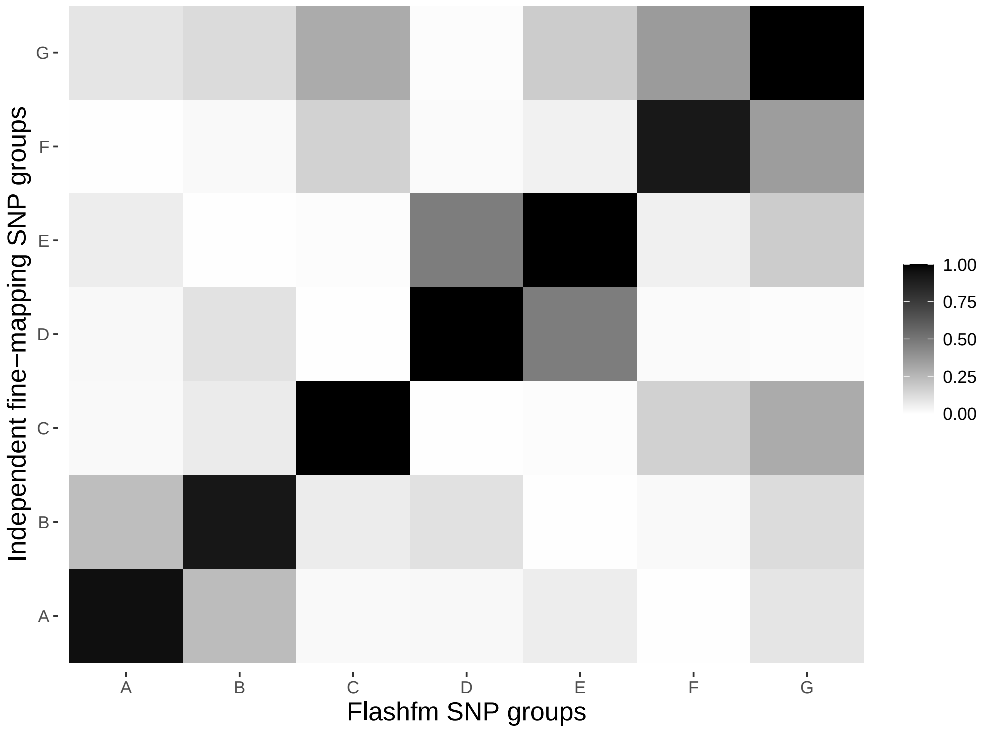

Supplementary Figure 38: Average LD between SNP groups constructed under independent fine-mapping (left) and Flashfm (right) for region 6:32559710-32671044.

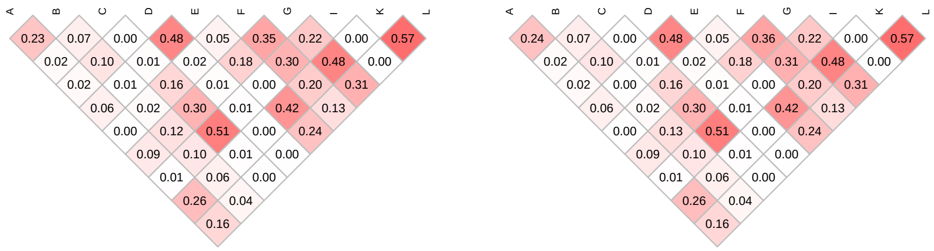

	Stepwise		Independent		Flashfm	
Traits	SNP/Model	P-value	Model	PP	Model	PP
mch	6:12369067/B	6.21894e-08	B	0.352	B	0.513
	–	–	B+I	0.148	B+I	0.110
mcv	6:12369067/B	6.72028e-07	B	0.326	B	0.451
	–	–	B+I	0.122	L	0.091
	–	–	L	0.063	B+I	0.086

Supplementary Table 41: Fine-mapping results for 6:12367517-12394380 by independent fine-mapping and Flashfm. Stepwise results are also given.

	SNP Group		
	B	I	L
Independent	1	10	1
	6:12369067	6:12390604	6:12368852
Flashfm	1	10	1
	6:12369067	6:12390604	6:12368852

Supplementary Table 42: SNP group sizes and representative variant for groups constructed by independent fine-mapping and by Flashfm for region 6:12367517-12394380.

Supplementary Figure 39: Average LD between SNP groups constructed under independent fine-mapping with those from flashfm for region 6:12367517-12394380.

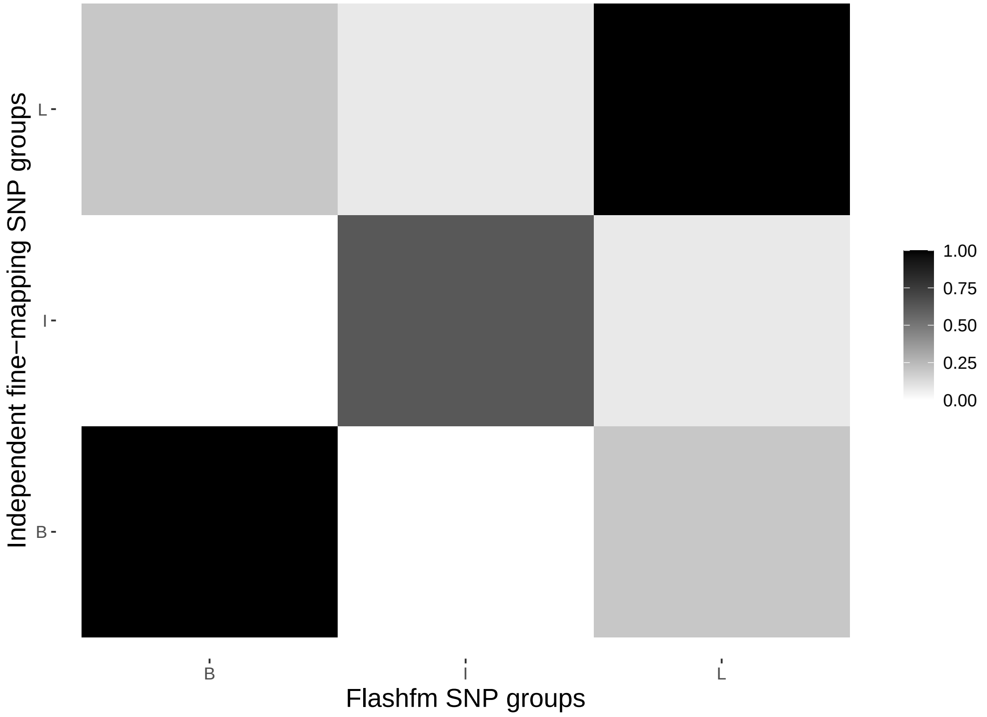

Supplementary Figure 40: Average LD between SNP groups constructed under independent fine-mapping (left) and Flashfm (right) for region 6:12367517-12394380.

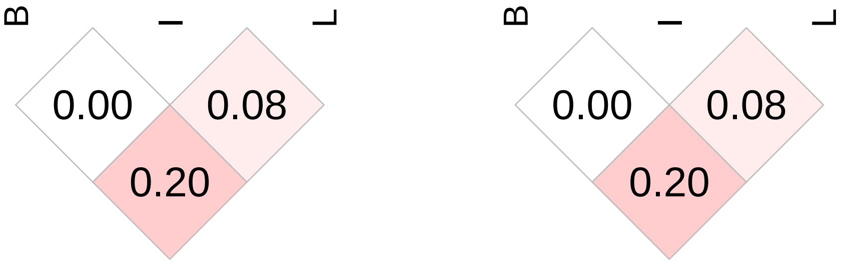

	Stepwise		Independent		Flashfm	
Traits	SNP/Model	P-value	Model	PP	Model	PP
mcv	6:167913072/A	2.0766e-07	A	0.618	A	0.708
mch	6:167913072/A	2.30335e-07	A	0.651	A	0.738

Supplementary Table 43: Fine-mapping results for 6:167874267-168050957 by independent fine-mapping and Flashfm. Stepwise results are also given.

	SNP Group
	A
Independent	3
	6:167919770
Flashfm	3
	6:167919770

Supplementary Table 44: SNP group sizes and representative variant for groups constructed by independent fine-mapping and by Flashfm for region 6:167874267-168050957.

Supplementary Figure 41: Venn diagram of SNP group constructed under indepenedent fine-mapping compared to that under flashfm for region 6:167874267-168050957. This shows that the flashfm group is a subset of, or the same as, the group form independent fine-mapping.

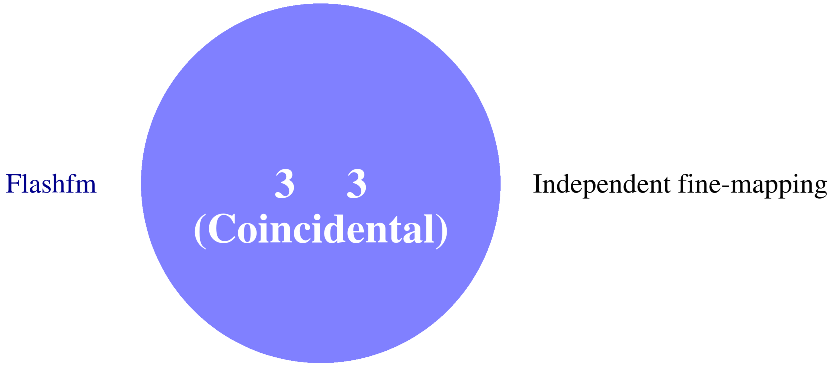

	Stepwise		Independent		Flashfm	
Traits	SNP/Model	P-value	Model	PP	Model	PP
hip_circum	8:6357093/A	5.68796e-08	A	0.792	A	0.773
weight	8:6357093/A	3.30175e-07	A	0.729	A	0.753

Supplementary Table 45: Fine-mapping results for 8:6353108-6394179 by independent fine-mapping and Flashfm. Stepwise results are also given.

	SNP Group
	A
Independent	18
	8:6357093
Flashfm	15
	8:6357093

Supplementary Table 46: SNP group sizes and representative variant for groups constructed by independent fine-mapping and by Flashfm for region 8:6353108-6394179.

Supplementary Figure 42: Venn diagram of SNP group constructed under indepenedent fine-mapping compared to that under flashfm for region 8:6353108-6394179. This shows that the flashfm group is a subset of, or the same as, the group form independent fine-mapping.

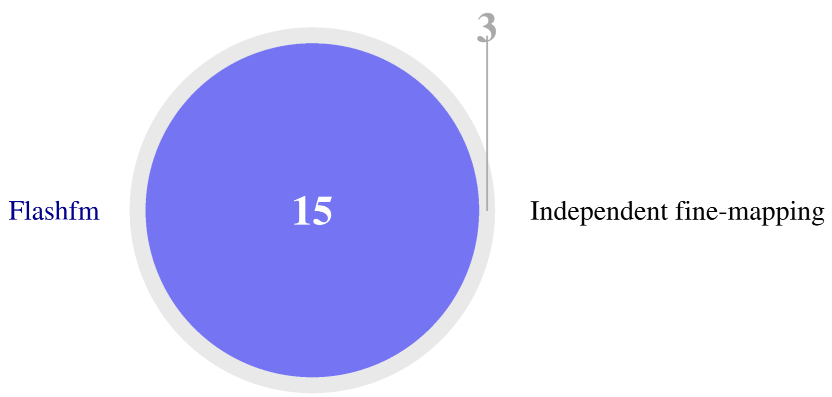

	Stepwise		Independent		Flashfm	
Traits	SNP/Model	P-value	Model	PP	Model	PP
rbc_dstr_width	11:5248232/A	1.26439e-16	A	0.667	A	0.752
	–	–	A+C	0.087	A+C	0.07
bilirubin	11:5243936/A	3.95972e-07	A	0.517	A	0.742
	–	–	B	0.192	–	–
	–	–	A+B	0.055	–	–

Supplementary Table 47: Fine-mapping results for 11:5211496-5252794 by independent fine-mapping and Flashfm. Stepwise results are also given.

	SNP Group		
	A	B	C
Independent	4	12	1
	11:5252794	11:5236954	11:5230135
Flashfm	4	5	1
	11:5252794	11:5236954	11:5230135

Supplementary Table 48: SNP group sizes and representative variant for groups constructed by independent fine-mapping and by Flashfm for region 11:5211496-5252794.

Supplementary Figure 43: Average LD between SNP groups constructed under independent fine-mapping with those from flashfm for region 11:5211496-5252794.

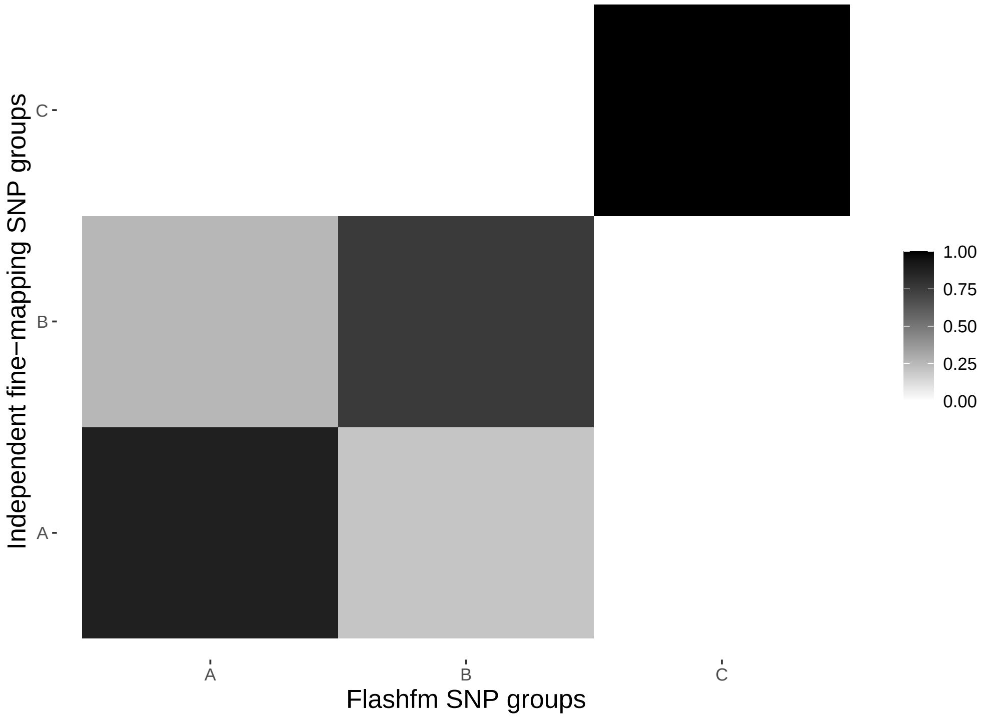

Supplementary Figure 44: Average LD between SNP groups constructed under independent fine-mapping (left) and Flashfm (right) for region 11:5211496-5252794.

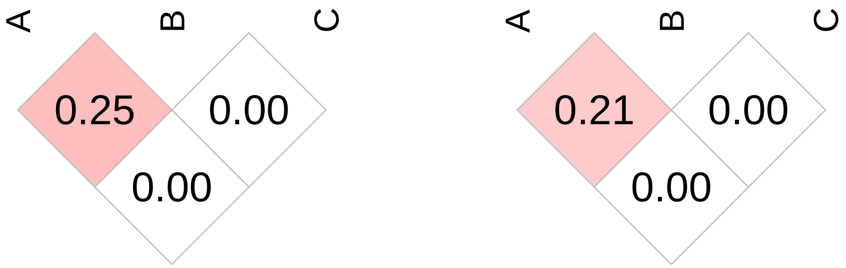

	Stepwise		Independent		Flashfm	
Traits	SNP/Model	P-value	Model	PP	Model	PP
rbc_dstr_width	11:5336134/A	1.02917e-14	A	0.619	A	0.684
bilirubin	11:5334665/A	3.0441e-07	A	0.713	A	0.733

Supplementary Table 49: Fine-mapping results for 11:5304648-5363496 by independent fine-mapping and Flashfm. Stepwise results are also given.

	SNP Group
	A
Independent	8
	11:5363496
Flashfm	3
	11:5340595

Supplementary Table 50: SNP group sizes and representative variant for groups constructed by independent fine-mapping and by Flashfm for region 11:5304648-5363496.

Supplementary Figure 45: Venn diagram of SNP group constructed under indepenedent fine-mapping compared to that under flashfm for region 11:5304648-5363496. This shows that the flashfm group is a subset of, or the same as, the group form independent fine-mapping.

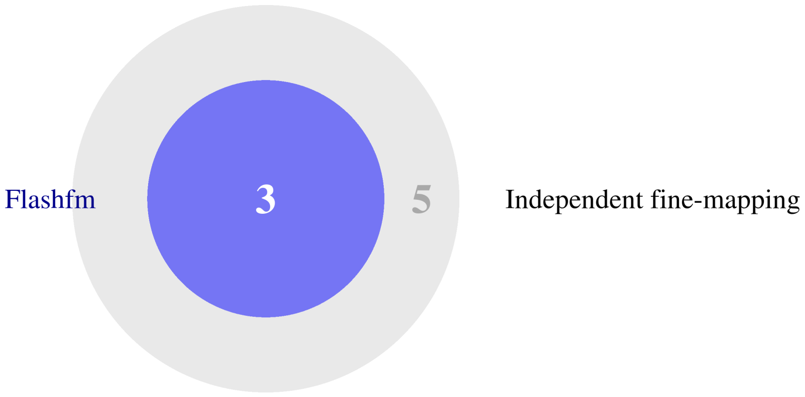

	Stepwise		Independent		Flashfm	
Traits	SNP/Model	P-value	Model	PP	Model	PP
rbc_dstr_width	11:5118578/A	2.08951e-13	A	0.701	A	0.885
mchc	11:5190723/A	2.34699e-07	A	0.593	A	0.724

Supplementary Table 51: Fine-mapping results for 11:5053165-5190723 by independent fine-mapping and Flashfm. Stepwise results are also given.

	SNP Group
	A
Independent	2
	11:5118578
Flashfm	2
	11:5190723

Supplementary Table 52: SNP group sizes and representative variant for groups constructed by independent fine-mapping and by Flashfm for region 11:5053165-5190723.

Supplementary Figure 46: Venn diagram of SNP group constructed under indepenedent fine-mapping compared to that under flashfm for region 11:5053165-5190723. This shows that the flashfm group is a subset of, or the same as, the group form independent fine-mapping.

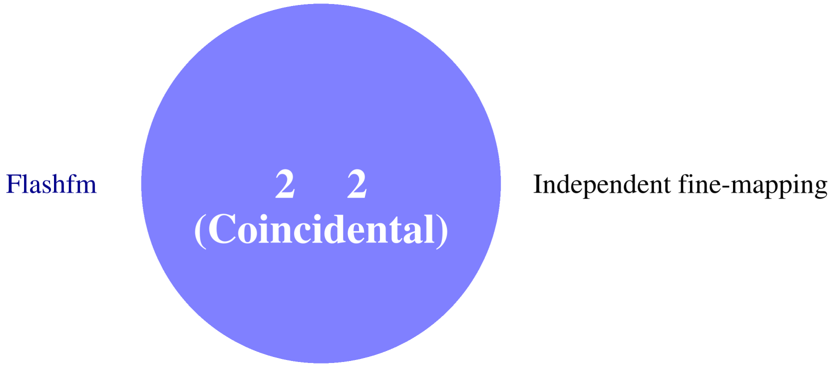

	Stepwise		Independent		Flashfm	
Traits	SNP/Model	P-value	Model	PP	Model	PP
cholesterol	11:84360985/B	2.20999e-07	B	0.823	B	0.818
lowdlipo	11:84360985/B	2.52085e-07	B	0.76	B	0.594
	–	–	–	–	B+D	0.078
	–	–	–	–	B+C	0.061
	–	–	–	–	A	0.059

Supplementary Table 53: Fine-mapping results for 11:84190922-84413365 by independent fine-mapping and Flashfm. Stepwise results are also given.

	SNP Group			
	A	B	C	D
Independent	1	12	4	3
	11:84356305	11:84342305	11:84262771	11:84349327
Flashfm	1	12	4	3
	11:84356305	11:84366910	11:84262771	11:84349327

Supplementary Table 54: SNP group sizes and representative variant for groups constructed by independent fine-mapping and by Flashfm for region 11:84190922-84413365.

Supplementary Figure 47: Average LD between SNP groups constructed under independent fine-mapping with those from flashfm for region 11:84190922-84413365.

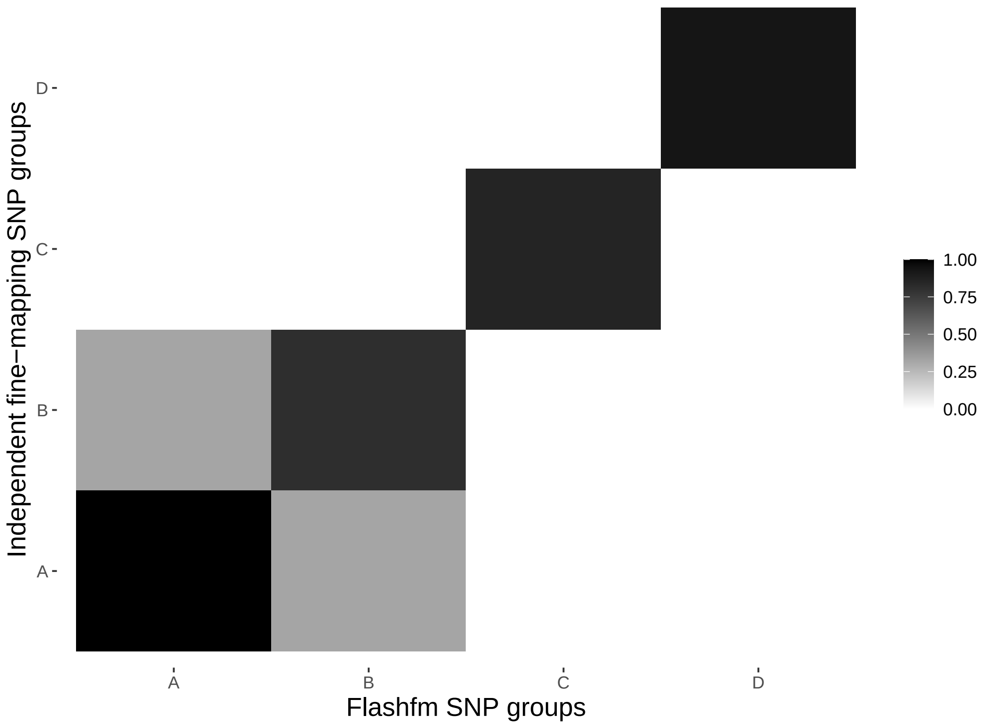

Supplementary Figure 48: Average LD between SNP groups constructed under independent fine-mapping (left) and Flashfm (right) for region 11:84190922-84413365.

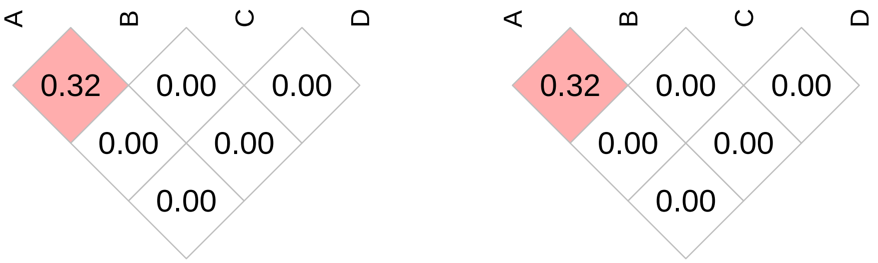

	Stepwise		Independent		Flashfm	
Traits	SNP/Model	P-value	Model	PP	Model	PP
hgb	13:23057646/A	4.47604e-07	A	0.662	A	0.744
hct	13:23057646/A	6.80465e-07	A	0.679	A	0.760

Supplementary Table 55: Fine-mapping results for 13:23033928-23144347 by independent fine-mapping and Flashfm. Stepwise results are also given.

	SNP Group
	A
Independent	4<br>13:23065854
Flashfm	4<br>13:23065854

Supplementary Table 56: SNP group sizes and representative variant for groups constructed by independent fine-mapping and by Flashfm for region 13:23033928-23144347.

Supplementary Figure 49: Venn diagram of SNP group constructed under indepenedent fine-mapping compared to that under flashfm for region 13:23033928-23144347. This shows that the flashfm group is a subset of, or the same as, the group form independent fine-mapping.

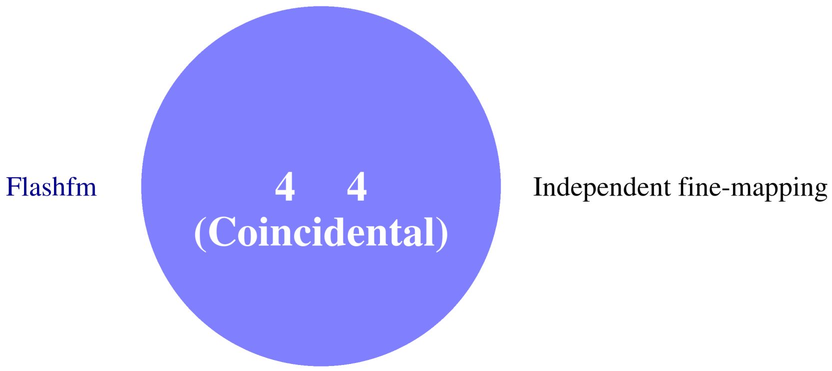

	Stepwise		Independent		Flashfm	
Traits	SNP/Model	P-value	Model	PP	Model	PP
weight	14:21748646/A	7.00214e-08	A	0.722	A	0.763
	–	–	A+F	0.080	A+F	0.057
waist_circum	14:21748646/A	1.49727e-07	A	0.756	A	0.786
bmi	14:21748646/A	2.63504e-07	A	0.764	A	0.803
	–	–	A+D	0.068	–	–

Supplementary Table 57: Fine-mapping results for 14:21673702-21797029 by independent fine-mapping and Flashfm. Stepwise results are also given.

	SNP Group		
	A	D	F
Independent	32	12	4
	14:21686690	14:21743242	14:21692999
Flashfm	8	12	4
	14:21744483	14:21743242	14:21692999

Supplementary Table 58: SNP group sizes and representative variant for groups constructed by independent fine-mapping and by Flashfm for region 14:21673702-21797029.

Supplementary Figure 50: Average LD between SNP groups constructed under independent fine-mapping with those from flashfm for region 14:21673702-21797029.

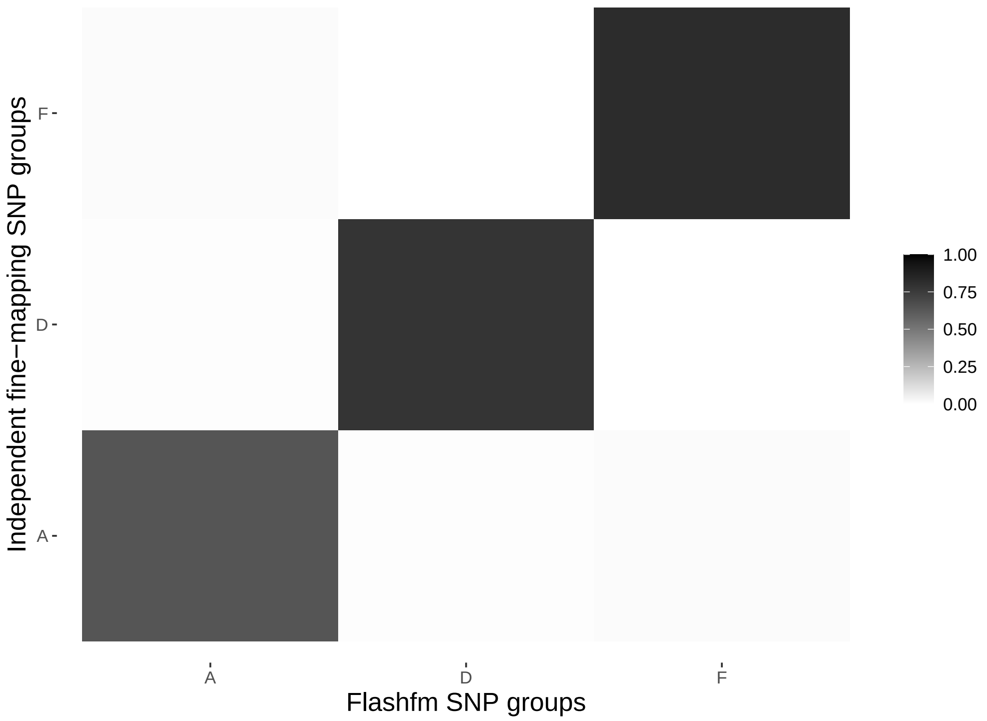

Supplementary Figure 51: Average LD between SNP groups constructed under independent fine-mapping (left) and Flashfm (right) for region 14:21673702-21797029.

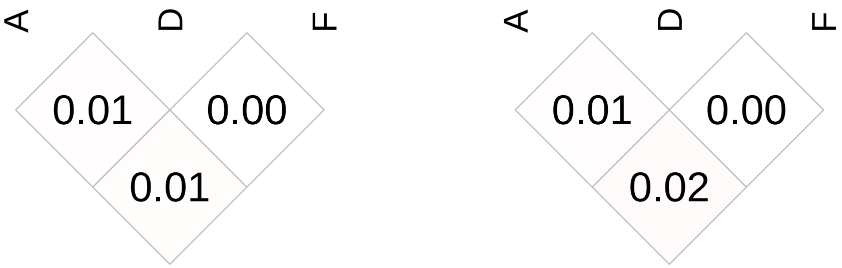

	Stepwise		Independent		Flashfm	
Traits	SNP/Model	P-value	Model	PP	Model	PP
highdlipo	15:58723675/G	5.53742e-14	G	0.427	G	0.523
	–	–	G+J	0.091	G+J	0.09
	–	–	C+G	0.076	C+G	0.064
	–	–	G+K	0.051	–	–
triglycerides	15:58723426/G	9.90278e-08	G	0.560	G	0.658

Supplementary Table 59: Fine-mapping results for 15:58718136-58742605 by independent fine-mapping and Flashfm. Stepwise results are also given.

	SNP Group			
	C	G	J	K
Independent	6	5	2	9
	15:58731665	15:58724842	15:58736623	15:58737382
Flashfm	6	4	2	7
	15:58731665	15:58723675	15:58736623	15:58735027

Supplementary Table 60: SNP group sizes and representative variant for groups constructed by independent fine-mapping and by Flashfm for region 15:58718136-58742605.

Supplementary Figure 52: Average LD between SNP groups constructed under independent fine-mapping with those from flashfm for region 15:58718136-58742605.

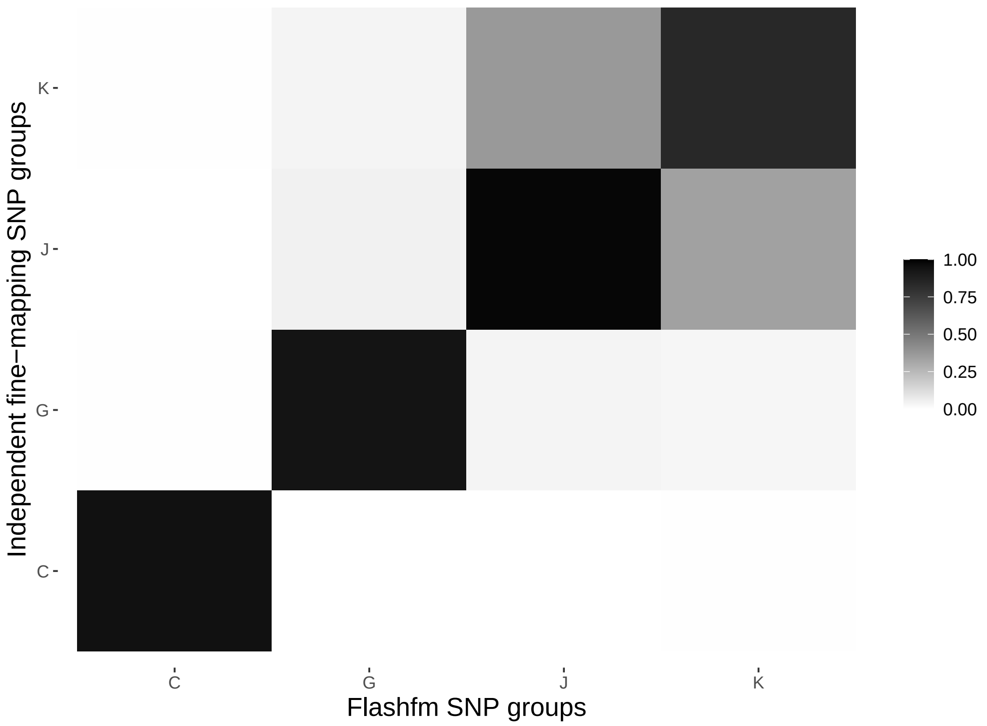

Supplementary Figure 53: Average LD between SNP groups constructed under independent fine-mapping (left) and Flashfm (right) for region 15:58718136-58742605.

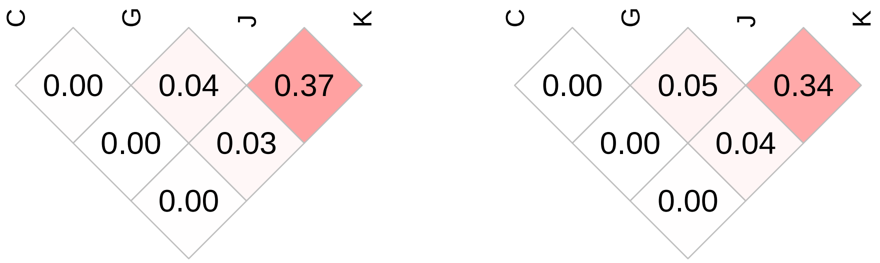

	Stepwise		Independent		Flashfm	
Traits	SNP/Model	P-value	Model	PP	Model	PP
cholesterol	15:55705675/A	1.71224e-07	A	0.365	A	0.545
	–	–	A+G	0.364	A+G	0.266
lowdlipo	15:55705675/A	4.20627e-07	A	0.581	A	0.738
	–	–	A+G	0.227	A+G	0.140

Supplementary Table 61: Fine-mapping results for 15:55547292-55822875 by independent fine-mapping and Flashfm. Stepwise results are also given.

	SNP Group	
	A	G
Independent	8	36
	15:55745354	15:55734203
Flashfm	8	36
	15:55745354	15:55734203

Supplementary Table 62: SNP group sizes and representative variant for groups constructed by independent fine-mapping and by Flashfm for region 15:55547292-55822875.

Supplementary Figure 54: Average LD between SNP groups constructed under independent fine-mapping with those from flashfm for region 15:55547292-55822875.

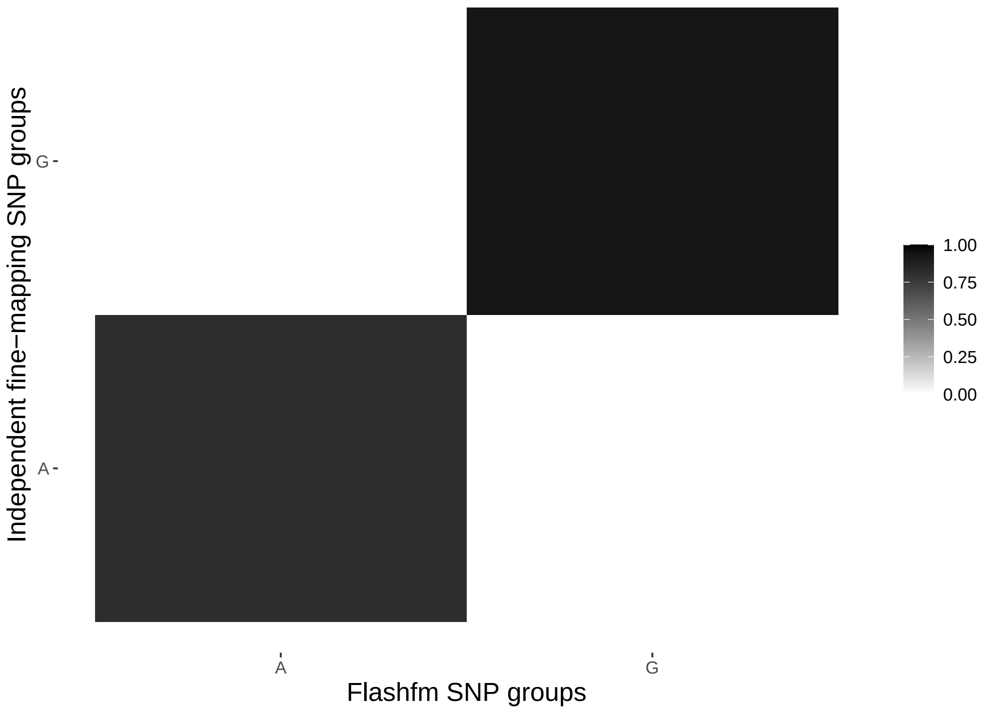

Supplementary Figure 55: Average LD between SNP groups constructed under independent fine-mapping (left) and Flashfm (right) for region 15:55547292-55822875.

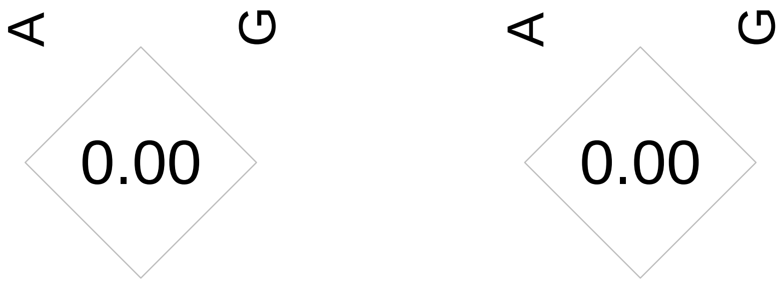

	Stepwise		Independent		Flashfm	
Traits	SNP/Model	P-value	Model	PP	Model	PP
rbc	16:90070/A	1.10783e-10	A	0.215	A	0.331
	–	–	A+T	0.170	A+T	0.148
	–	–	A+B	0.111	A+B	0.096
	–	–	A+B+T	0.108	A+B+T	0.058
	–	–	A+M	0.059	A+M	0.055
mchc	16:88553/A	1.38189e-10	A+C	0.413	A+C	0.494
	–	–	A	0.291	A	0.302
hba1c2	16:90599/A	1.72663e-08	A+H+N	0.427	A+H+N	0.393
	16:88607/H	5.72399e-09	A+H	0.181	A+H	0.254
	–	–	A+H+K	0.074	A+H+J	0.067
	–	–	A+H+M	0.070	A+H+K	0.066
	–	–	A+H+K+N	0.064	A+H+M	0.066
mch	16:90070/A	2.74381e-24	A+C+D+G+J+U	0.414	A+C+D+G+J+U	0.327
	16:102581/J	2.01668e-09	A+C+G+J+M	0.159	A+C+G+J	0.187
	16:130429/G	3.15454e-07	A+C+G+J	0.110	A+C+G+J+M	0.173
	16:117331/C	1.01688e-07	A+C+D+G+J	0.089	A+C+D+G+J	0.098
	–	–	A+C+G+J+U	0.064	A+C+G+J+U	0.078

Supplementary Table 63: Fine-mapping results for 16:60647-137716 by independent fine-mapping and Flashfm. Stepwise results are also given.

	SNP Group						
	A	B	C	D	G	H	J
Independent	4	1	2	3	1	5	8
	16:90599	16:104329	16:117050	16:134745	16:130429	16:88607	16:94853
Flashfm	4	1	2	3	1	5	8
	16:90599	16:104329	16:117331	16:134745	16:130429	16:88607	16:94853
	K	M	N	T	U		
Independent	1	7	6	9	1		
	16:130406	16:137634	16:128054	16:88245	16:103592		
Flashfm	1	7	6	9	1		
	16:130406	16:137634	16:128054	16:88245	16:103592		

Supplementary Table 64: SNP group sizes and representative variant for groups constructed by independent fine-mapping and by Flashfm for region 16:60647-137716.

Supplementary Figure 56: Average LD between SNP groups constructed under independent fine-mapping with those from flashfm for region 16:60647-137716.

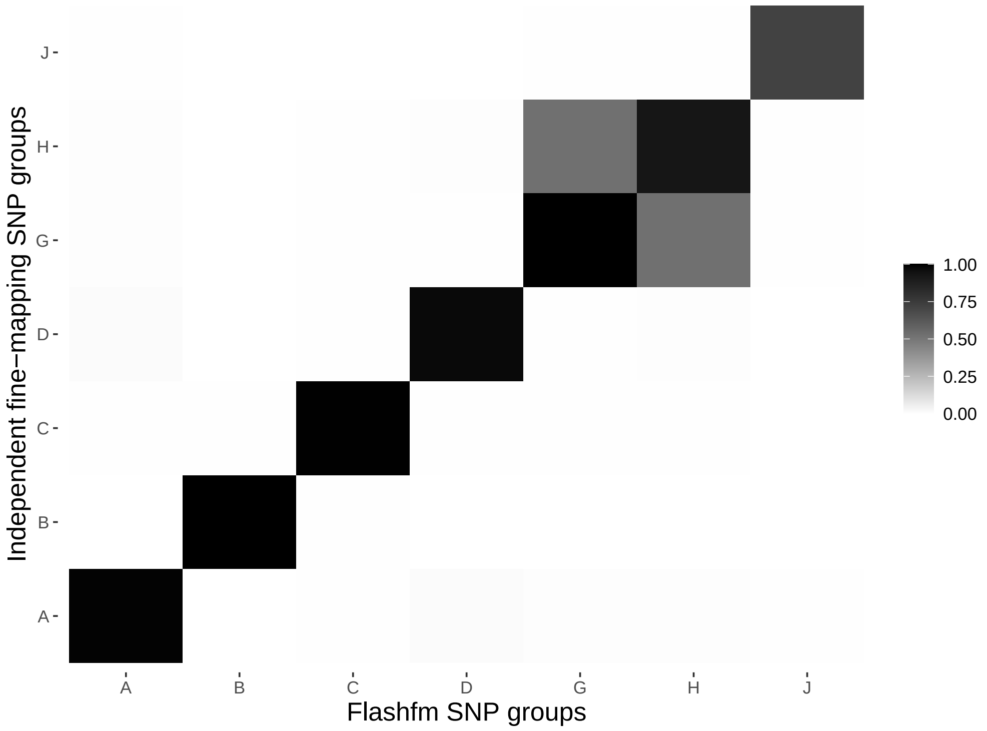

Supplementary Figure 57: Average LD between SNP groups constructed under independent fine-mapping (left) and Flashfm (right) for region 16:60647-137716.

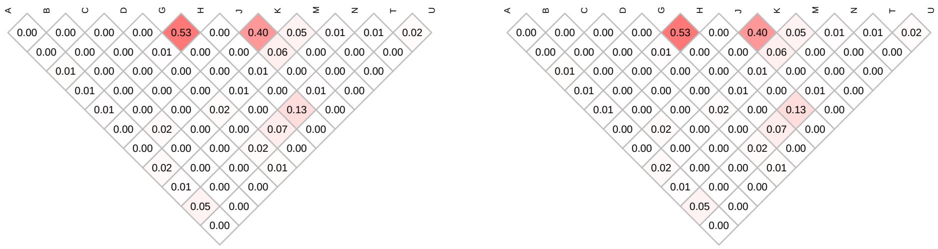

	Stepwise		Independent		Flashfm	
Traits	SNP/Model	P-value	Model	PP	Model	PP
bilirubin	16:603562/B	1.14991e-07	B	0.295	B	0.531
	–	–	B+L	0.254	B+L	0.271
	–	–	C	0.142	–	–
	–	–	C+L	0.072	–	–
rbc	16:603562/B	1.54871e-07	B+D	0.511	B+D	0.591
	–	–	B+D+F	0.086	B	0.094
	–	–	A+D	0.056	B+D+F	0.075
	–	–	B	0.053	–	–
mcv	16:603562/B	7.55658e-14	B+D+F	0.519	B+D+F	0.599
	16:542642/F	1.70737e-12	B+D+F+M	0.092	B+D+F+M	0.077
	16:527280/D	7.88461e-12	–	–	–	–

Supplementary Table 65: Fine-mapping results for 16:498355-605776 by independent fine-mapping and Flashfm. Stepwise results are also given.

	SNP Group						
	A	B	C	D	F	L	M
Independent	1	11	1	4	3	30	2
	16:594790	16:600756	16:594329	16:527280	16:526278	16:553945	16:553917
Flashfm	0	1	1	4	3	30	2
	–	16:603562	16:594329	16:527280	16:526278	16:553945	16:553917

Supplementary Table 66: SNP group sizes and representative variant for groups constructed by independent fine-mapping and by Flashfm for region 16:498355-605776.

Supplementary Figure 58: Average LD between SNP groups constructed under independent fine-mapping with those from flashfm for region 16:498355-605776.

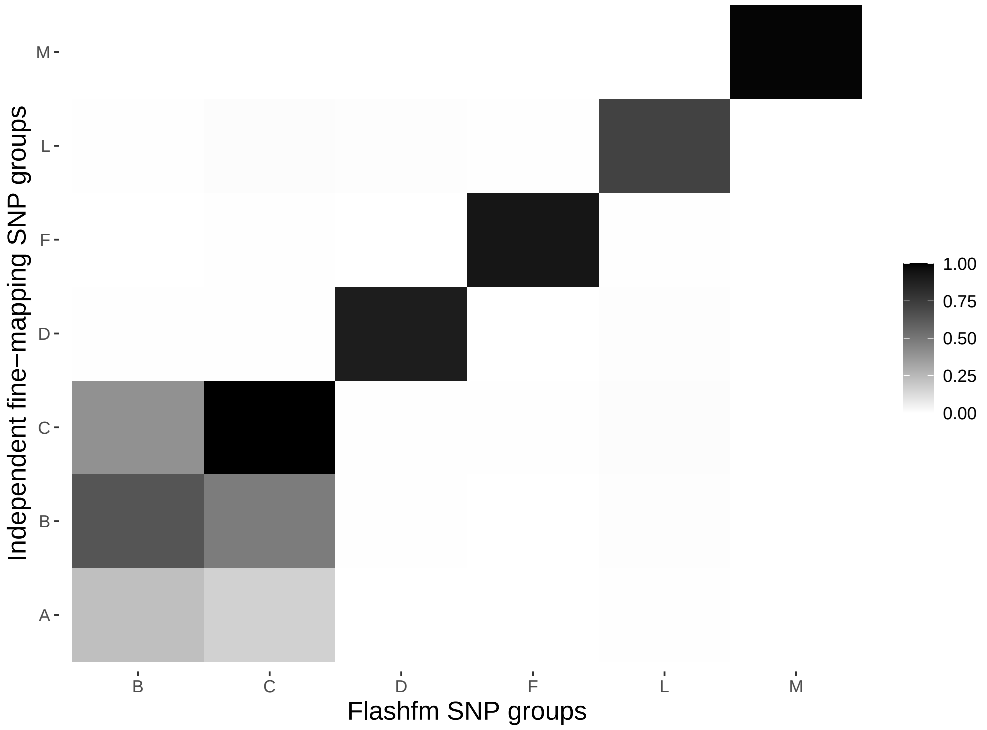

Supplementary Figure 59: Average LD between SNP groups constructed under independent fine-mapping (left) and Flashfm (right) for region 16:498355-605776.

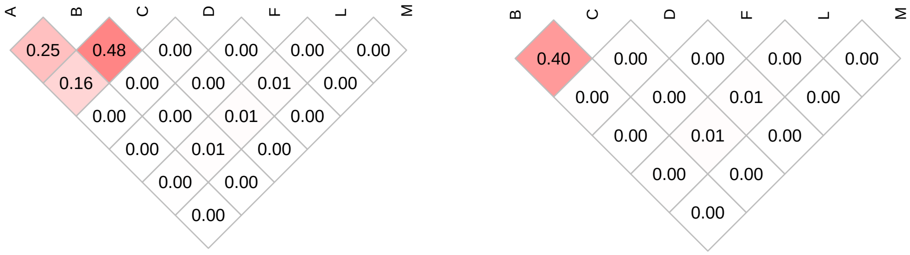

	Stepwise		Independent		Flashfm	
Traits	SNP/Model	P-value	Model	PP	Model	PP
mch	16:1027250/C	7.09192e-10	C	0.584	C	0.722
	–	–	A+C	0.161	A+C	0.098
mcv	16:1027250/C	2.93089e-09	C	0.414	C	0.583
	–	–	A+C	0.295	A+C	0.205

Supplementary Table 67: Fine-mapping results for 16:939366-1043324 by independent fine-mapping and Flashfm. Stepwise results are also given.

	SNP Group	
	A	C
Independent	4	5
	16:949809	16:1027250
Flashfm	4	5
	16:949809	16:1027250

Supplementary Table 68: SNP group sizes and representative variant for groups constructed by independent fine-mapping and by Flashfm for region 16:939366-1043324.

Supplementary Figure 60: Average LD between SNP groups constructed under independent fine-mapping with those from flashfm for region 16:939366-1043324.

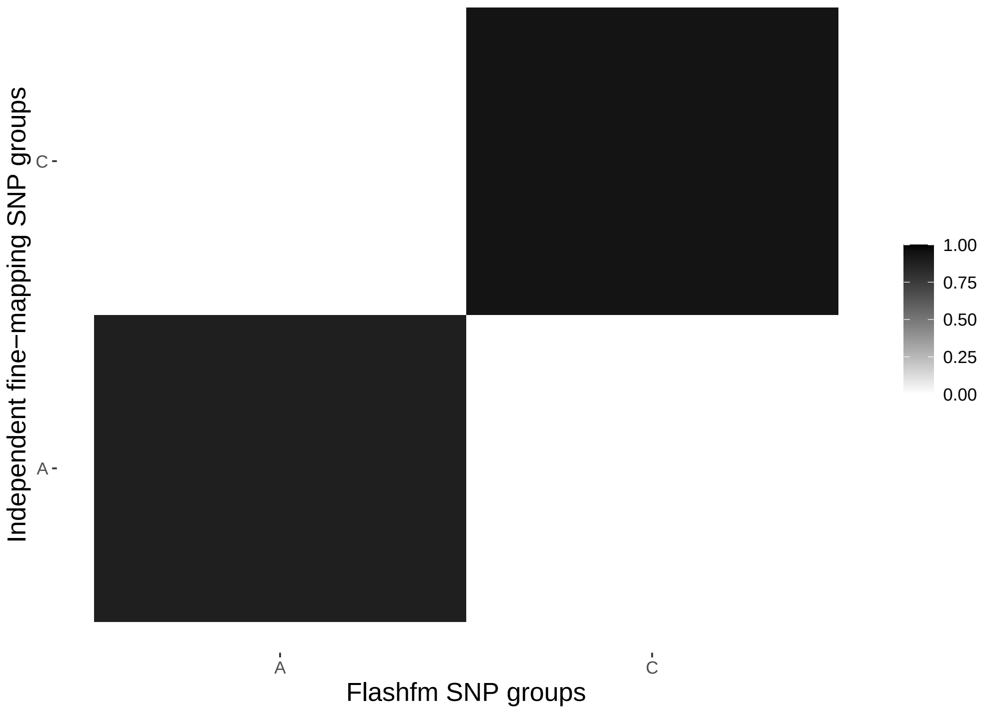

Supplementary Figure 61: Average LD between SNP groups constructed under independent fine-mapping (left) and Flashfm (right) for region 16:939366-1043324.

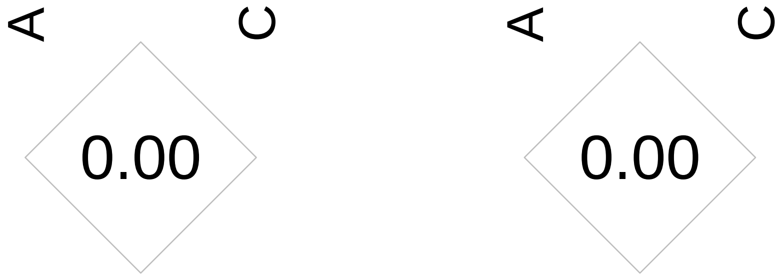

	Stepwise		Independent		Flashfm	
Traits	SNP/Model	P-value	Model	PP	Model	PP
highdlipo	16:56995935/B	1.1908e-49	B+D+H+M+T	0.269	B+E+J+S+T	0.148
	16:56987369/M	4.38062e-17	B+E+J+S+T	0.061	B+D+H+M+T	0.116
	16:57002092/T	2.07704e-12	B+D+M+T	0.055	B+C+J+S+T	0.092
	–	–	B+E+J+M+T	0.053	B+F+J+S+T	0.087
	–	–	B+E+M+T	0.052	B+E+S+T	0.080
cholesterol	16:56991363/S	6.2228e-08	S	0.739	S	0.730
	–	–	–	–	S+T	0.050
triglycerides	16:56995827/B	1.21097e-07	B+K	0.55	B+K	0.604
	–	–	B	0.063	B	0.106
	–	–	K	0.056	K+S	0.059
neut_count	16:56987960/A	7.75024e-07	A	0.562	A	0.322
	–	–	–	–	A+S	0.231
	–	–	–	–	A+B	0.153

Supplementary Table 69: Fine-mapping results for 16:56971385-57018102 by independent fine-mapping and Flashfm. Stepwise results are also given.

	SNP Group						
	A	B	C	D	E	F	H
Independent	4	3	9	1	2	1	10
	16:56987960	16:56990827	16:57017002	16:57007610	16:57007734	16:57007451	16:56999960
Flashfm	2	2	4	1	2	1	3
	16:56987960	16:56995827	16:57002152	16:57007610	16:57007734	16:57007451	16:56998761
	J	K	M	S	T		
Independent	1	11	3	6	2		
	16:56996060	16:57009985	16:56987015	16:56993324	16:57002092		
Flashfm	1	11	3	5	2		
	16:56996060	16:57009985	16:56987015	16:56996211	16:57002092		

Supplementary Table 70: SNP group sizes and representative variant for groups constructed by independent fine-mapping and by Flashfm for region 16:56971385-57018102.

Supplementary Figure 62: Average LD between SNP groups constructed under independent fine-mapping with those from flashfm for region 16:56971385-57018102.

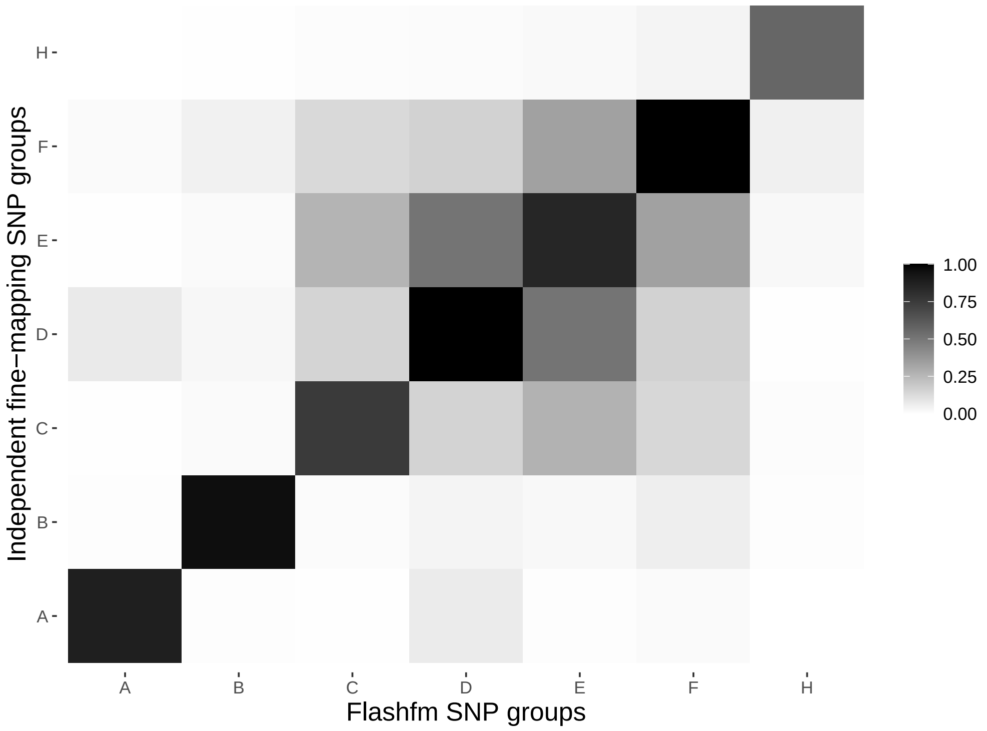

Supplementary Figure 63: Average LD between SNP groups constructed under independent fine-mapping (left) and Flashfm (right) for region 16:56971385-57018102.

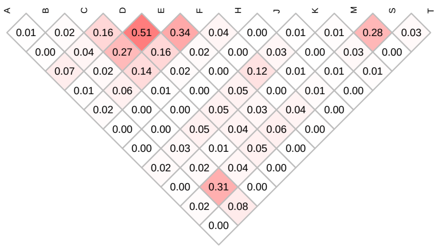

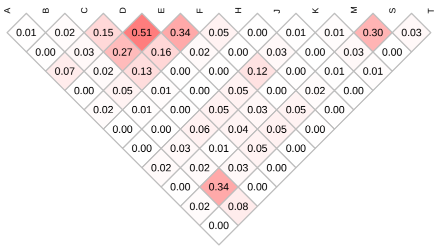

	Stepwise		Independent		Flashfm	
Traits	SNP/Model	P-value	Model	PP	Model	PP
mcv	16:167165/D	6.25413e-19	A2+C2+D+E+I+X	0.202	A2+C2+D+E+I+X	0.429
	16:190254/E	1.5333e-13	A+A2+C2+D+E+I+R+X	0.127	A2+C2+D+E+I+R+X	0.136
	16:172646/C2	5.61875e-08	A+A2+C2+D+E+I	0.123	A2+C2+D+E+I+P+X	0.09
	–	–	A+A2+C2+D+E+I+X	0.112	A2+C2+D+E+I+Y	0.055
	–	–	A+A2+C2+D+E+I+R	0.099	–	–
	–	–	A2+C2+D+E+I+R+X	0.076	–	–
	–	–	A+A2+C2+D+E+I+Y	0.050	–	–
mchc	16:167165/D	5.79002e-11	D+I	0.362	D+I	0.362
	–	–	D+H	0.346	D+H	0.349
	–	–	D	0.087	D	0.148
hba1c2	16:150764/U	5.87248e-08	D+F+K	0.375	D+F+K	0.416
	–	–	D+F+K+Q	0.094	D+F+K+Q	0.057
	–	–	D+F+K+W	0.066	D+F+K+W	0.053
rbc	16:163626/D	4.1364e-08	D+E	0.257	D	0.426
	–	–	D	0.192	D+E	0.236
	–	–	D+E+Y	0.108	D+F	0.09
	–	–	D+F	0.080	D+Y	0.054
	–	–	D+Y	0.063	–	–

Supplementary Table 71: Fine-mapping results for 16:132064-198333 by independent fine-mapping and Flashfm. Stepwise results are also given.

	SNP Group						
	A	C	D	E	F	H	I
Independent	2	1	10	7	2	13	12
	16:140490	16:137591	16:167165	16:193088	16:167408	16:195176	16:149393
Flashfm	2	0	3	5	2	13	12
	16:140490	–	16:167165	16:193088	16:167408	16:195176	16:149393
	K	P	Q	R	W	X	Y
Independent	1	2	3	1	2	1	1
	16:196263	16:141060	16:153657	16:192741	16:153048	16:187520	16:173899
Flashfm	1	2	3	1	2	1	1
	16:196263	16:141060	16:153657	16:192741	16:153048	16:187520	16:173899
	A2	C2					
Independent	1	1					
	16:194759	16:172646					
Flashfm	1	1					
	16:194759	16:172646					

Supplementary Table 72: SNP group sizes and representative variant for groups constructed by independent fine-mapping and by Flashfm for region 16:132064-198333.

Supplementary Figure 64: Average LD between SNP groups constructed under independent fine-mapping with those from flashfm for region 16:132064-198333.

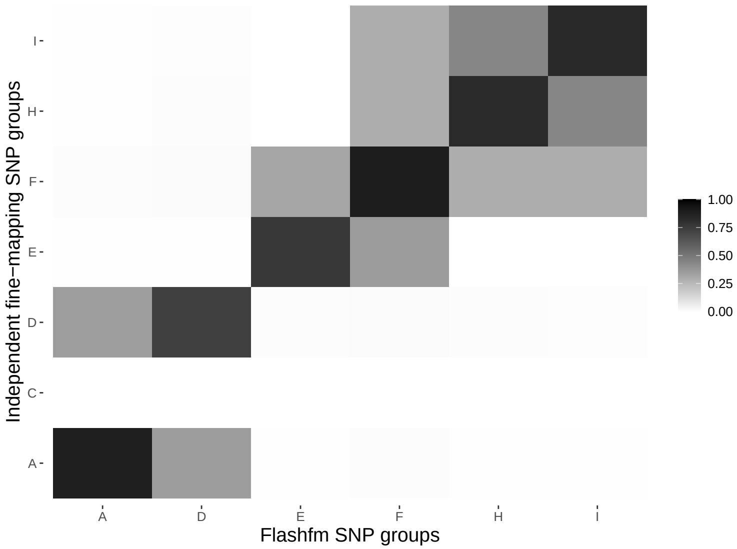

Supplementary Figure 65: Average LD between SNP groups constructed under independent fine-mapping (left) and Flashfm (right) for region 16:132064-198333.

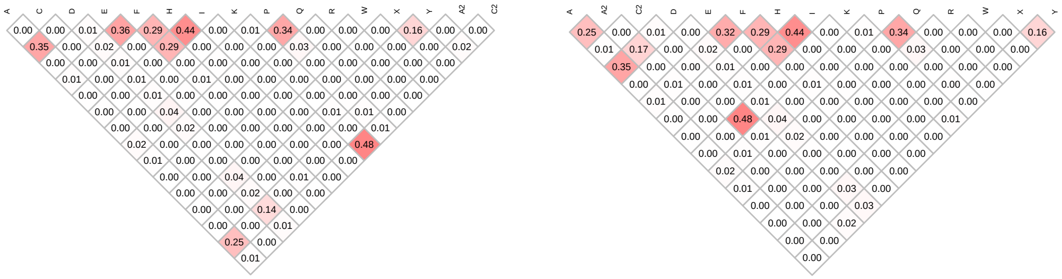

	Stepwise		Independent		Flashfm	
Traits	SNP/Model	P-value	Model	PP	Model	PP
highdlipo	16:57068393/D	5.21357e-21	A+D+E+P	0.383	A+D+E+P	0.565
	16:57077683/P	4.50615e-10	D+E+I+P	0.153	A+D+G+P	0.092
	16:57047299/A	1.41616e-07	D+E+P	0.098	A+E+J+P	0.064
	–	–	A+D+G+P	0.063	A+E+P	0.060
triglycerides	16:57078233/M	4.05655e-07	A+M	0.260	A+M	0.433
	–	–	M	0.142	A+M+N	0.137
	–	–	A+M+N	0.115	A+M+P	0.130
	–	–	A+M+P	0.089	A+M+O	0.054

Supplementary Table 73: Fine-mapping results for 16:57042326-57084037 by independent fine-mapping and Flashfm. Stepwise results are also given.

	SNP Group						
	A	D	E	G	I	J	M
Independent	2	6	3	8	1	3	3
	16:57047299	16:57042326	16:57050200	16:57058057	16:57070005	16:57046931	16:57078228
Flashfm	2	8	3	8	1	3	3
	16:57047299	16:57042326	16:57050200	16:57058057	16:57070005	16:57046931	16:57078228
	N	O	P				
Independent	2	1	2				
	16:57052730	16:57051833	16:57077683				
Flashfm	2	1	2				
	16:57052730	16:57051833	16:57077683				

Supplementary Table 74: SNP group sizes and representative variant for groups constructed by independent fine-mapping and by Flashfm for region 16:57042326-57084037.

Supplementary Figure 66: Average LD between SNP groups constructed under independent fine-mapping with those from flashfm for region 16:57042326-57084037.

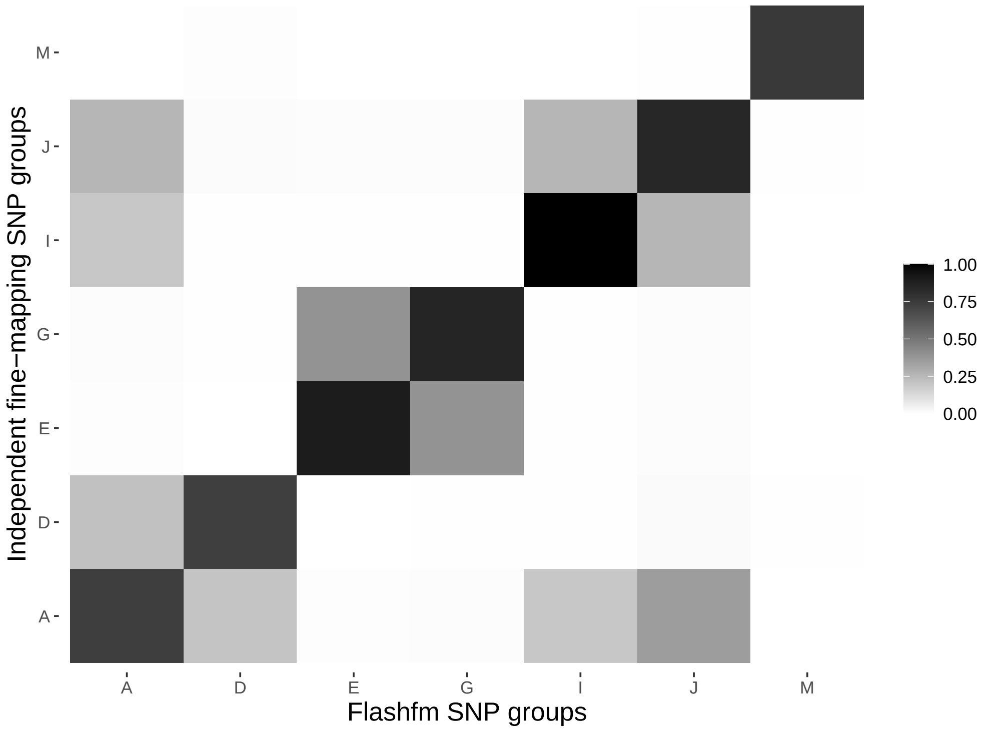

Supplementary Figure 67: Average LD between SNP groups constructed under independent fine-mapping (left) and Flashfm (right) for region 16:57042326-57084037.

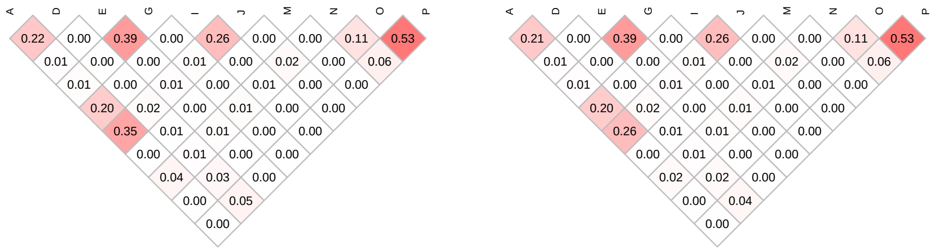

	Stepwise		Independent		Flashfm	
Traits	SNP/Model	P-value	Model	PP	Model	PP
bilirubin	16:321605/G	9.08431e-12	G	0.972	G	0.972
rbc	16:311107/G	4.2898e-10	G+J	0.988	G+J	0.988
	16:366048/J	2.87208e-09	G+J	0.988	G+J	0.988
mchc	16:302161/G	8.37996e-10	G+J	0.605	G+J	0.605
	16:366048/J	1.85719e-07	D+G+J	0.27	D+G+J	0.27
	–	–	G+H	0.054	G+H	0.054
hba1c2	16:321605/G	1.16483e-11	G	0.745	G	0.745
	–	–	C+G	0.089	C.1+G	0.089
mcv	16:311107/G	3.75442e-25	C+G+J	0.43	C.2+G+J	0.407
	16:366048/J	2.57128e-21	C+D+G+J	0.33	C.2+D+G+J	0.314
	16:368259/C	1.28459e-10	C+G+J+K	0.066	C.2+G+J+K	0.063
mch	16:311107/G	1.12816e-27	C+D+G+J+K	0.486	C.2+D+G+J+K	0.482
	16:366048/J	4.36966e-23	A+D+G+J+K	0.367	A+D+G+J+K	0.367
	16:368259/C	2.4743e-10	B+D+G+J+K	0.088	B+D+G+J+K	0.088
	16:308732/D	7.46373e-08	–	–	–	–

Supplementary Table 75: Fine-mapping results for 16:302161-440165 by independent fine-mapping and Flashfm. Stepwise results are also given.

	SNP Group						
	A	B	C	D	G	H	J
Independent	1	1	5	3	5	1	6
	16:320750	16:324837	16:347850	16:314962	16:321605	16:320865	16:366048
Flashfm	1	1	0	3	5	1	6
	16:320750	16:324837	–	16:314962	16:321605	16:320865	16:366048
	K	C.1	C.2				
Independent	2	0	0				
	16:349810	–	–				
Flashfm	2	3	2				
	16:349810	16:347850	16:368259				

Supplementary Table 76: SNP group sizes and representative variant for groups constructed by independent fine-mapping and by Flashfm for region 16:302161-440165.

Supplementary Figure 68: Average LD between SNP groups constructed under independent fine-mapping with those from flashfm for region 16:302161-440165.

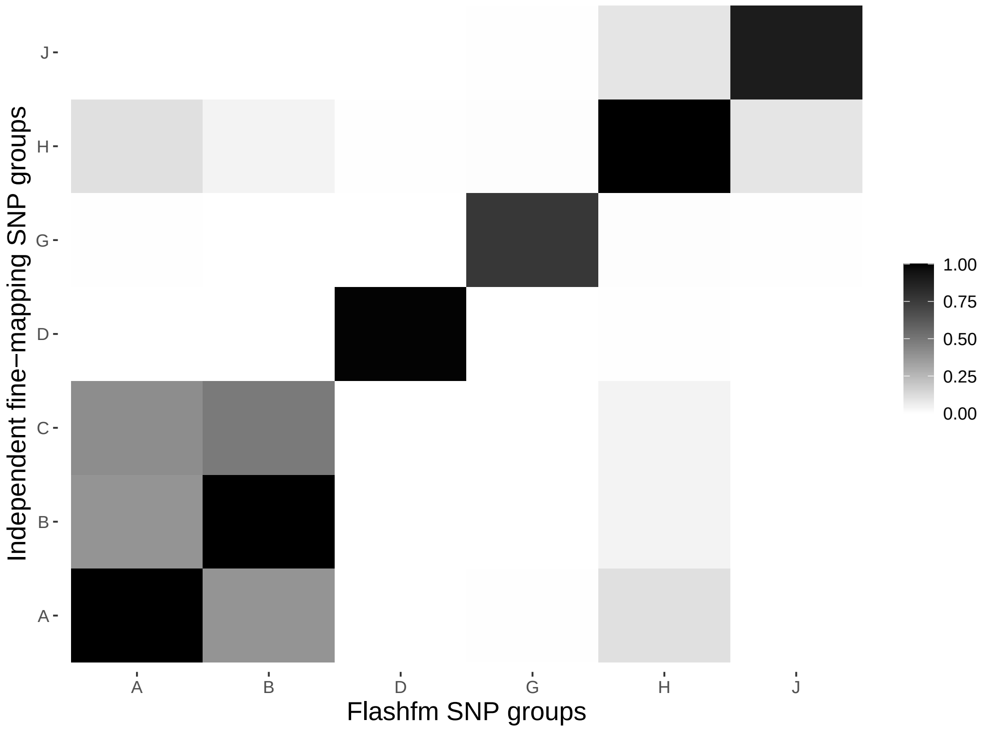

Supplementary Figure 69: Average LD between SNP groups constructed under independent fine-mapping (left) and Flashfm (right) for region 16:302161-440165.

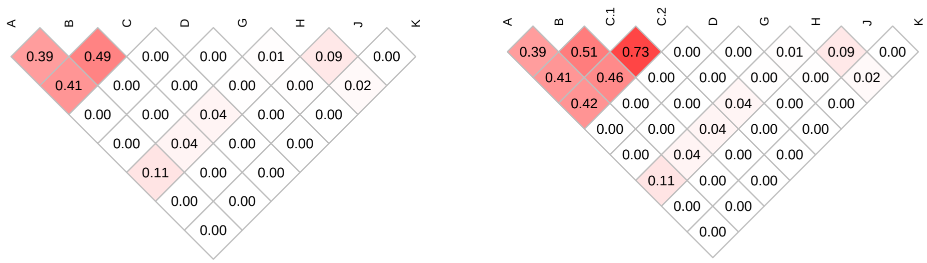

	Stepwise		Independent		Flashfm	
Traits	SNP/Model	P-value	Model	PP	Model	PP
mch	16:603562/V	4.70522e-15	K+M+V+W	0.435	K+M+V+W	0.425
	16:600319/M	7.56208e-10	K+M+V	0.108	K+M+V	0.2
	16:603871/K	1.67585e-07	L+M+V+W	0.105	L+M+V+W	0.104
mcv	16:603562/V	7.64402e-14	K+M+V	0.452	K+M+V	0.583
	16:600319/M	7.442e-10	K+M+V+W	0.102	K+M+V+W	0.069
	16:603871/K	5.48307e-07	K+M+N+V	0.050	–	–
rbc	16:603562/V	1.55295e-07	V	0.259	V	0.517
	–	–	C	0.097	E+V	0.07
	–	–	T	0.072	M+V	0.056
	–	–	E+V	0.052	–	–
bilirubin	16:603562/V	1.15022e-07	V	0.252	V	0.536
	–	–	U	0.166	I+V	0.071
	–	–	C	0.052	L+V	0.06
	–	–	I+V	0.051	–	–

Supplementary Table 77: Fine-mapping results for 16:589792-618334 by independent fine-mapping and Flashfm. Stepwise results are also given.

	SNP Group						
	C	E	I	K	L	M	N
Independent	15	1	2	3	3	3	1
	16:618334	16:600974	16:604953	16:605794	16:601614	16:594263	16:615720
Flashfm	0	1	2	3	3	3	1
	–	16:600974	16:604953	16:605794	16:601614	16:594263	16:615720
	T	U	V	W			
Independent	1	1	1	1			
	16:594790	16:594329	16:603562	16:608254			
Flashfm	1	0	2	1			
	16:594790	–	16:603562	16:608254			

Supplementary Table 78: SNP group sizes and representative variant for groups constructed by independent fine-mapping and by Flashfm for region 16:589792-618334.

Supplementary Figure 70: Average LD between SNP groups constructed under independent fine-mapping with those from flashfm for region 16:589792-618334.

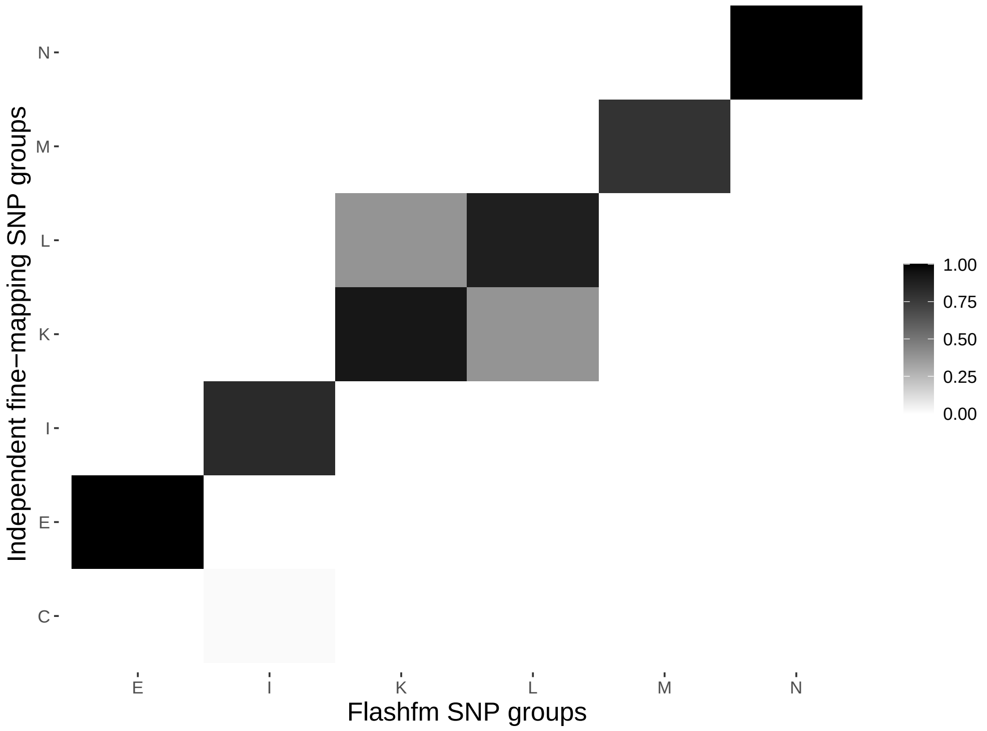

Supplementary Figure 71: Average LD between SNP groups constructed under independent fine-mapping (left) and Flashfm (right) for region 16:589792-618334.

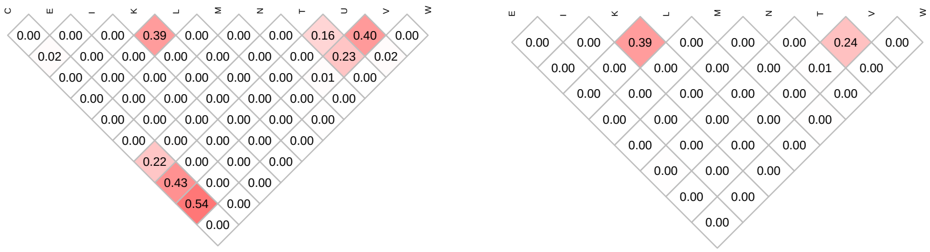

	Stepwise		Independent		Flashfm	
Traits	SNP/Model	P-value	Model	PP	Model	PP
bilirubin	16:603562/C	1.14991e-07	C	0.313	C	0.705
	–	–	A	0.206	–	–
	–	–	B	0.064	–	–
	–	–	Q	0.052	–	–
rbc	16:603562/C	1.54871e-07	C	0.215	C	0.553
	–	–	C+I	0.137	C+I	0.229
	–	–	F	0.072	–	–
	–	–	B	0.07	–	–
	–	–	E	0.059	–	–
mcv	16:603562/C	7.55658e-14	C+H+I	0.619	C+H+I	0.688
	16:600319/I	7.52621e-10	C+H+I+L	0.063	–	–
	16:603871/H	5.51157e-07	–	–	–	–

Supplementary Table 79: Fine-mapping results for 16:594329-743845 by independent fine-mapping and Flashfm. Stepwise results are also given.

	SNP Group						
	A	B	C	E	F	H	I
Independent	1	18	1	1	2	3	20
	16:594329	16:600756	16:603562	16:594790	16:621310	16:605794	16:600319
Flashfm	0	0	2	0	0	3	20
	–	–	16:603562	–	–	16:605794	16:600319
	L	Q					
Independent	3	3					
	16:681587	16:729825					
Flashfm	3	1					
	16:681587	16:730830					

Supplementary Table 80: SNP group sizes and representative variant for groups constructed by independent fine-mapping and by Flashfm for region 16:594329-743845.

Supplementary Figure 72: Average LD between SNP groups constructed under independent fine-mapping with those from flashfm for region 16:594329-743845.

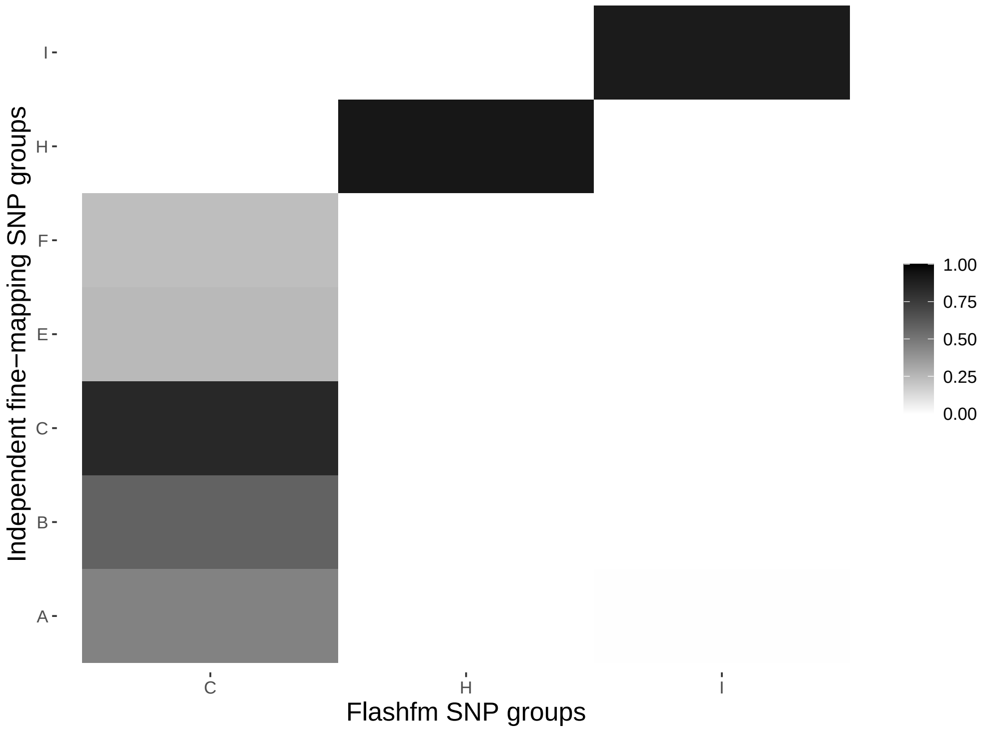

Supplementary Figure 73: Average LD between SNP groups constructed under independent fine-mapping (left) and Flashfm (right) for region 16:594329-743845.

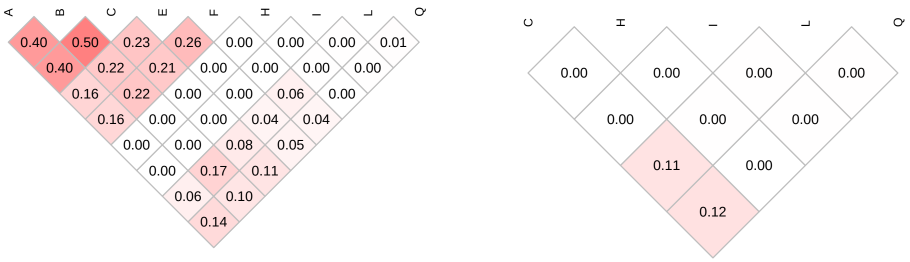

	Stepwise		Independent		Flashfm	
Traits	SNP/Model	P-value	Model	PP	Model	PP
mcv	16:482557/L	3.25875e-11	A+B+C+L	0.423	A+B+C+L	0.457
	16:542642/C	8.51277e-12	A+C+L	0.104	A+C+L	0.187
	16:527280/A	1.63876e-11	A+B+C+G+L	0.095	A+B+C+G+L	0.066
	–	–	A+B+C+F+L	0.064	–	–
mch	16:482557/L	3.63114e-11	A+B+C+L	0.165	A+B+C+L	0.267
	16:527280/A	1.46122e-11	A+B+C+D+L	0.159	A+B+C+D+L	0.164
	16:542642/C	1.06721e-10	A+B+C+L+M	0.140	A+B+C+L+M	0.145
	16:542041/B	4.2608e-08	A+B+C+D+L+M	0.103	A+B+C+D+L+M	0.074
	–	–	A+B+C+D+F+L	0.055	–	–
bilirubin	16:482557/L	1.18192e-07	L	0.450	L	0.632
	–	–	E+L	0.186	E+L	0.131

Supplementary Table 81: Fine-mapping results for 16:441156-557188 by independent fine-mapping and Flashfm. Stepwise results are also given.

	SNP Group						
	A	B	C	D	E	F	G
Independent	14	1	2	2	6	1	2
	16:527280	16:542041	16:542642	16:532430	16:548573	16:542420	16:553919
Flashfm	14	1	2	2	6	1	2
	16:527280	16:542041	16:542642	16:532430	16:548573	16:542420	16:553919
	L	M					
Independent	4	1					
	16:445085	16:530988					
Flashfm	1	1					
	16:482557	16:530988					

Supplementary Table 82: SNP group sizes and representative variant for groups constructed by independent fine-mapping and by Flashfm for region 16:441156-557188.

Supplementary Figure 74: Average LD between SNP groups constructed under independent fine-mapping with those from flashfm for region 16:441156-557188.

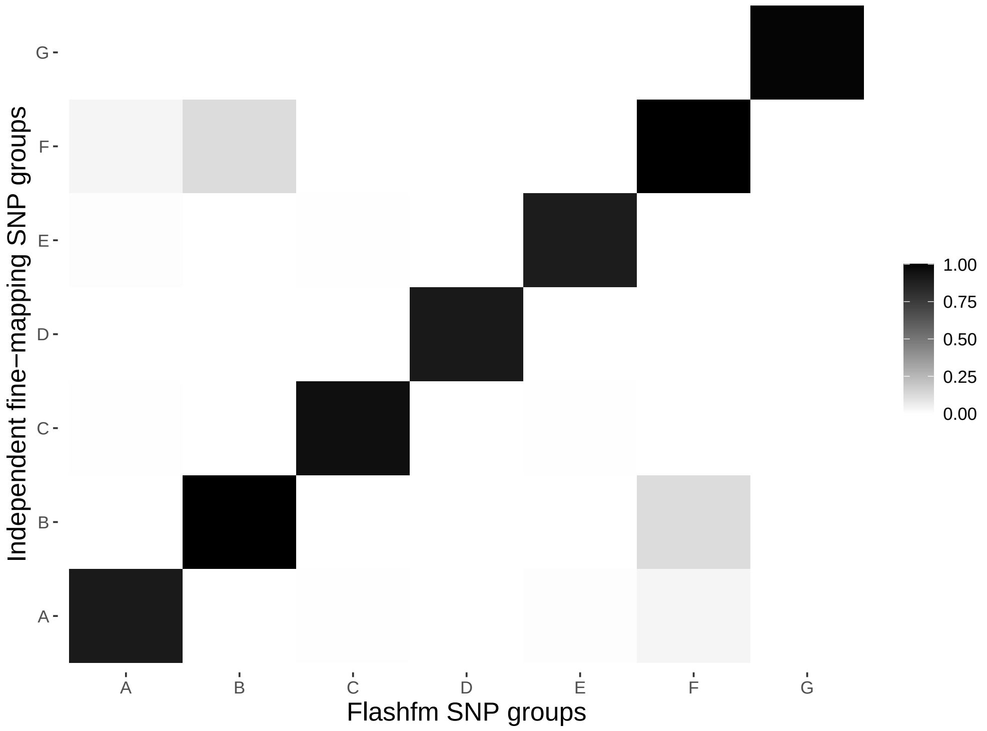

Supplementary Figure 75: Average LD between SNP groups constructed under independent fine-mapping (left) and Flashfm (right) for region 16:441156-557188.

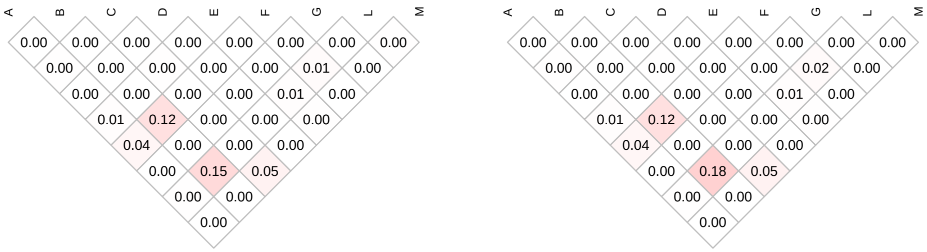

	Stepwise		Independent		Flashfm	
Traits	SNP/Model	P-value	Model	PP	Model	PP
mch	16:911357/D	3.02107e-09	D+F	0.433	D+F	0.373
	–	–	D	0.208	D	0.36
mcv	16:911357/D	8.6022e-08	D	0.323	D	0.491
	–	–	D+F	0.272	D+F	0.195
	–	–	D+E	0.064	–	–

Supplementary Table 83: Fine-mapping results for 16:898109-936815 by independent fine-mapping and Flashfm. Stepwise results are also given.

	SNP Group		
	D	E	F
Independent	2	18	1
	16:911357	16:919395	16:902670
Flashfm	2	11	1
	16:911357	16:904358	16:902670

Supplementary Table 84: SNP group sizes and representative variant for groups constructed by independent fine-mapping and by Flashfm for region 16:898109-936815.

Supplementary Figure 76: Average LD between SNP groups constructed under independent fine-mapping with those from flashfm for region 16:898109-936815.

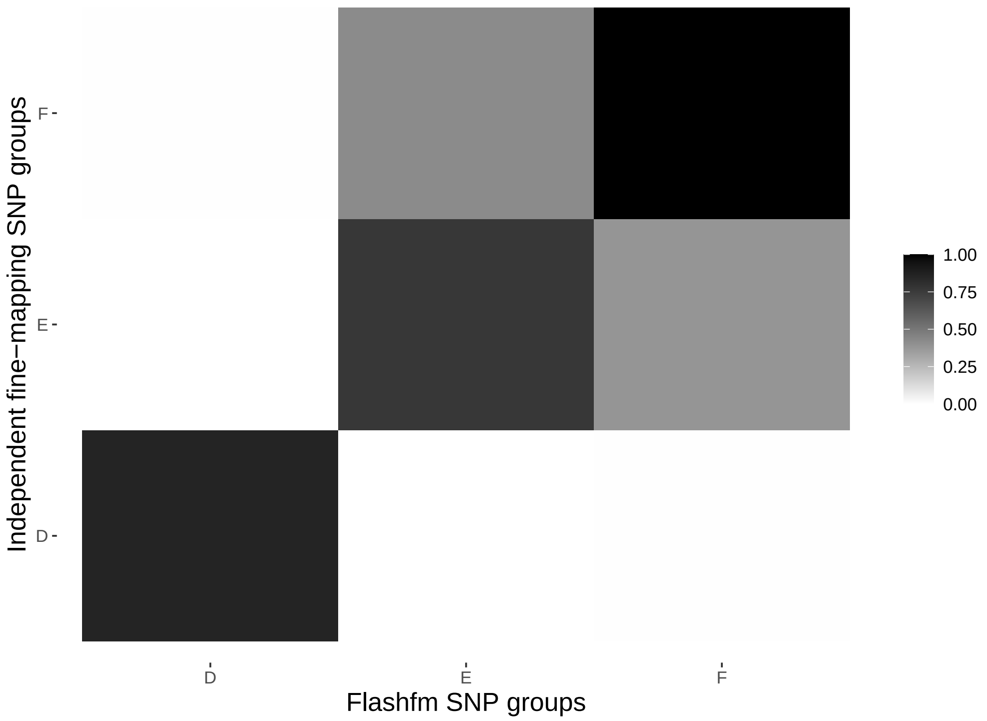

Supplementary Figure 77: Average LD between SNP groups constructed under independent fine-mapping (left) and Flashfm (right) for region 16:898109-936815.

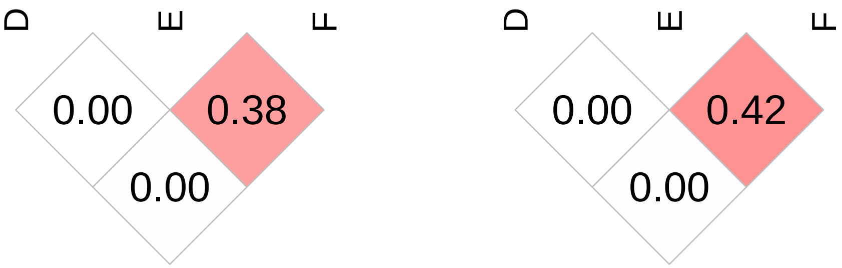

	Stepwise		Independent		Flashfm	
Traits	SNP/Model	P-value	Model	PP	Model	PP
hba1c2	16:771234/J	1.41831e-08	J	0.547	J	0.383
	–	–	B+J	0.057	I+J	0.275
	–	–	–	–	I	0.085
mcv	16:775654/L	1.86525e-07	A+I	0.467	A+I	0.513
	–	–	A+E+I	0.082	A+F+I	0.051
	–	–	A+F+I	0.05	–	–
bilirubin	16:750762/K	3.7458e-07	K	0.779	K	0.638
	–	–	–	–	I	0.092
	–	–	–	–	I+K	0.068

Supplementary Table 85: Fine-mapping results for 16:729957-794205 by independent fine-mapping and Flashfm. Stepwise results are also given.

	SNP Group						
	A	B	E	F	I	J	K
Independent	1	10	14	1	4	3	22
	16:784765	16:767430	16:772848	16:776983	16:783004	16:766690	16:741581
Flashfm	1	13	10	1	4	4	22
	16:784765	16:766001	16:772848	16:776983	16:783004	16:771286	16:741581

Supplementary Table 86: SNP group sizes and representative variant for groups constructed by independent fine-mapping and by Flashfm for region 16:729957-794205.

Supplementary Figure 78: Average LD between SNP groups constructed under independent fine-mapping with those from flashfm for region 16:729957-794205.

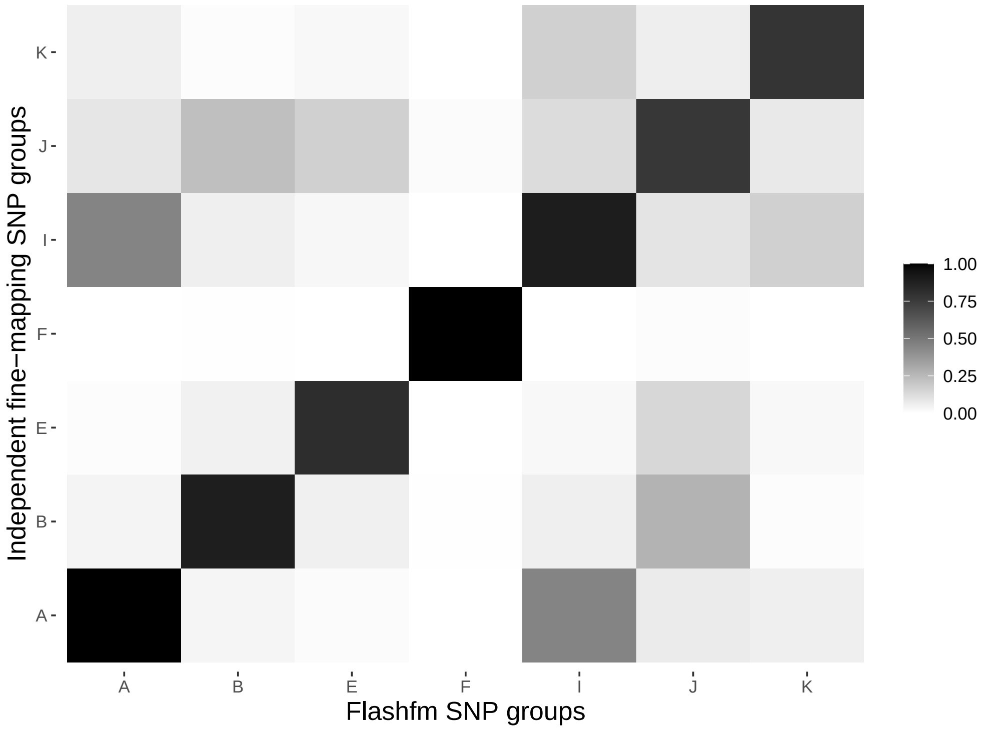

Supplementary Figure 79: Average LD between SNP groups constructed under independent fine-mapping (left) and Flashfm (right) for region 16:729957-794205.

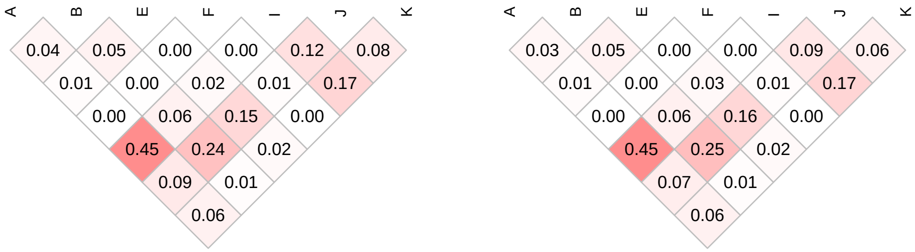

	Stepwise		Independent		Flashfm	
Traits	SNP/Model	P-value	Model	PP	Model	PP
mcv	16:881333/B	1.10539e-07	B+E	0.343	B	0.312
	–	–	B	0.209	B+E	0.305
	–	–	B+K	0.063	B+K	0.056
mch	16:907039/D	2.32685e-08	D+J	0.181	D	0.248
	–	–	D	0.142	D+J	0.153
	–	–	B	0.072	B	0.105
	–	–	B+K	0.068	B+K	0.057
	–	–	B+E	0.053	–	–

Supplementary Table 87: Fine-mapping results for 16:863336-907039 by independent fine-mapping and Flashfm. Stepwise results are also given.

	SNP Group				
	B	D	E	J	K
Independent	4	1	2	1	1
	16:882657	16:907039	16:896321	16:902670	16:867961
Flashfm	4	1	2	1	1
	16:882657	16:907039	16:896321	16:902670	16:867961

Supplementary Table 88: SNP group sizes and representative variant for groups constructed by independent fine-mapping and by Flashfm for region 16:863336-907039.

Supplementary Figure 80: Average LD between SNP groups constructed under independent fine-mapping with those from flashfm for region 16:863336-907039.

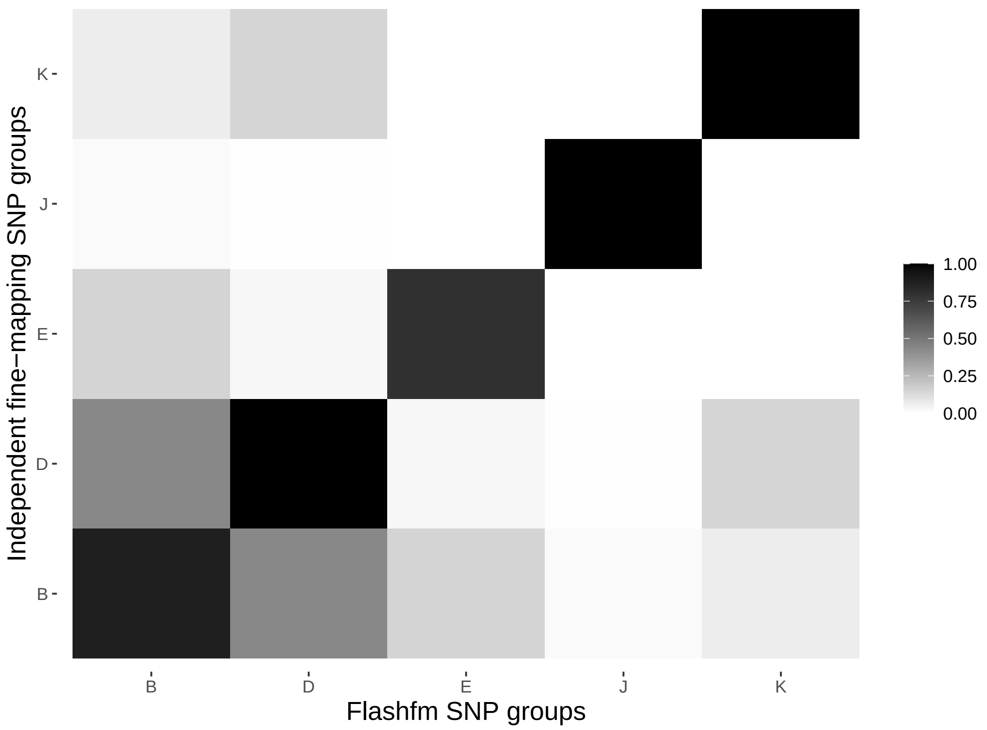

Supplementary Figure 81: Average LD between SNP groups constructed under independent fine-mapping (left) and Flashfm (right) for region 16:863336-907039.

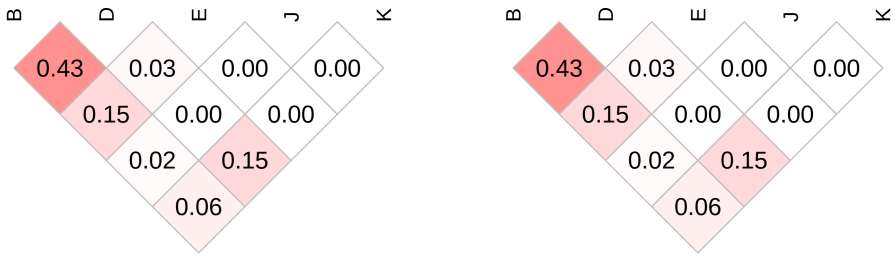

	Stepwise		Independent		Flashfm	
Traits	SNP/Model	P-value	Model	PP	Model	PP
cholesterol	17:75278052/G	1.64919e-08	G	0.575	G	0.693
lowdlipo	17:75278824/K	1.72825e-07	K	0.429	K	0.498
	–	–	H	0.116	H	0.135
	–	–	G	0.068	G	0.074

Supplementary Table 89: Fine-mapping results for 17:75251326-75306310 by independent fine-mapping and Flashfm. Stepwise results are also given.

	SNP Group		
	G	H	K
Independent	3	3	2
	17:75286406	17:75255973	17:75278824
Flashfm	3	3	2
	17:75286406	17:75255973	17:75278824

Supplementary Table 90: SNP group sizes and representative variant for groups constructed by independent fine-mapping and by Flashfm for region 17:75251326-75306310.

Supplementary Figure 82: Average LD between SNP groups constructed under independent fine-mapping with those from flashfm for region 17:75251326-75306310.

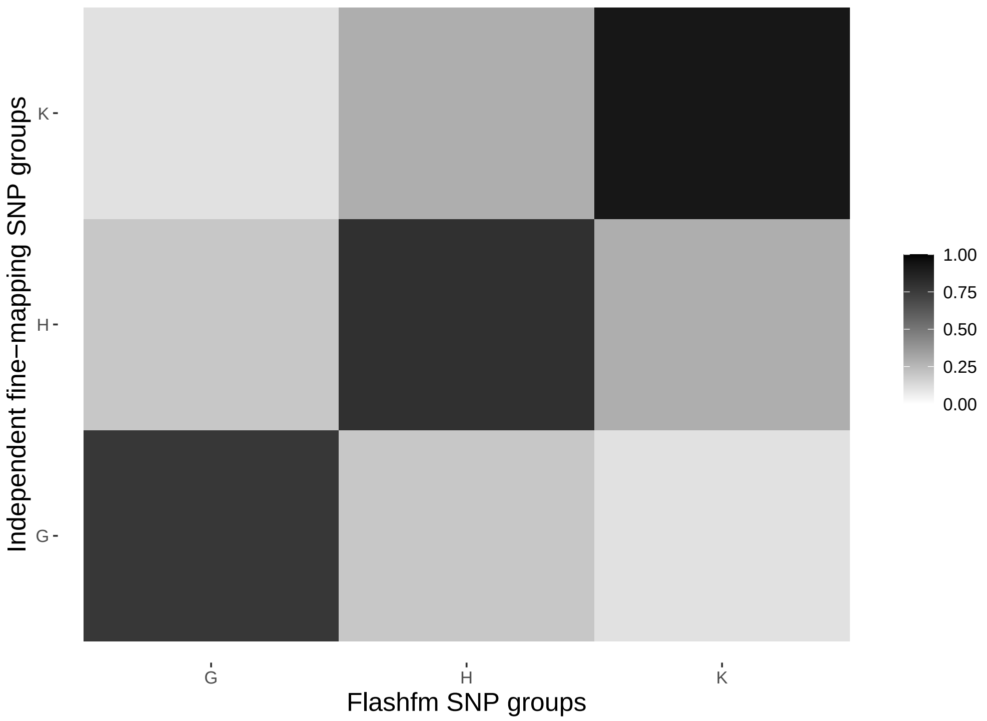

Supplementary Figure 83: Average LD between SNP groups constructed under independent fine-mapping (left) and Flashfm (right) for region 17:75251326-75306310.

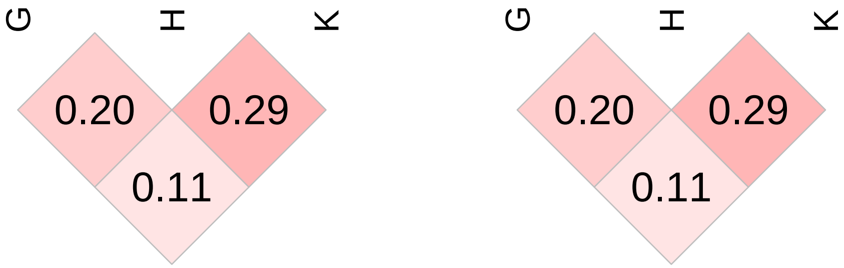

	Stepwise		Independent		Flashfm	
Traits	SNP/Model	P-value	Model	PP	Model	PP
hip_circum	18:8236712/A	4.45867e-07	A	0.546	A	0.650
	–	–	C	0.101	C	0.062
	–	–	A+E	0.059	A+E	0.052
waist_circum	18:8236712/A	4.74792e-07	A	0.690	A	0.773

Supplementary Table 91: Fine-mapping results for 18:8173997-8257404 by independent fine-mapping and Flashfm. Stepwise results are also given.

	SNP Group		
	A	C	E
Independent	5	18	1
	18:8257404	18:8219357	18:8240811
Flashfm	5	11	1
	18:8257404	18:8219357	18:8240811

Supplementary Table 92: SNP group sizes and representative variant for groups constructed by independent fine-mapping and by Flashfm for region 18:8173997-8257404.

Supplementary Figure 84: Average LD between SNP groups constructed under independent fine-mapping with those from flashfm for region 18:8173997-8257404.

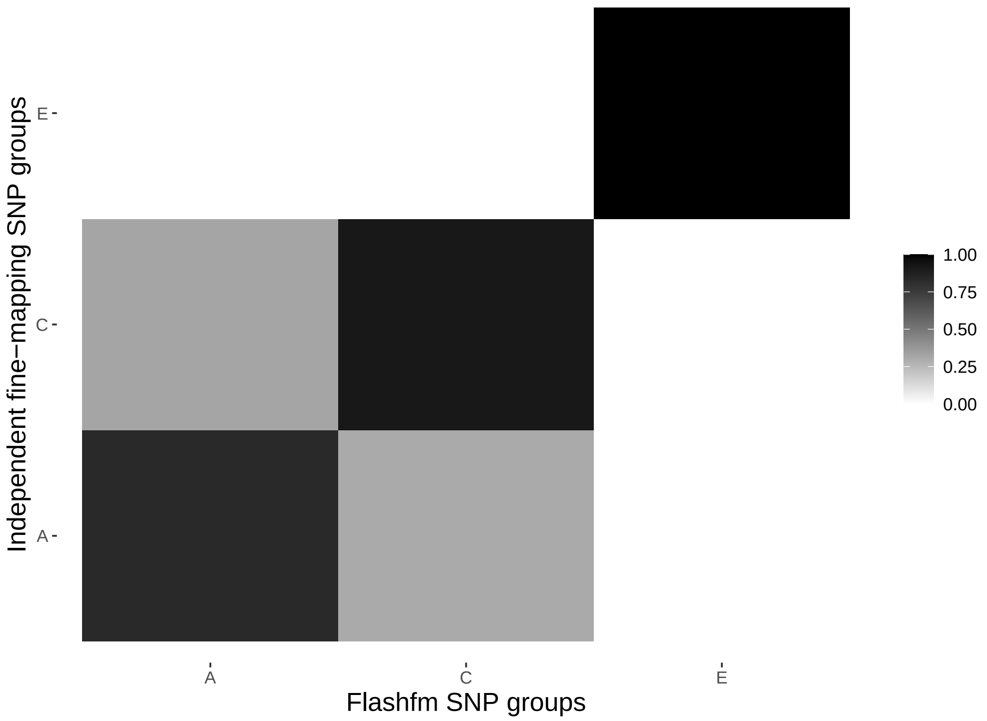

Supplementary Figure 85: Average LD between SNP groups constructed under independent fine-mapping (left) and Flashfm (right) for region 18:8173997-8257404.

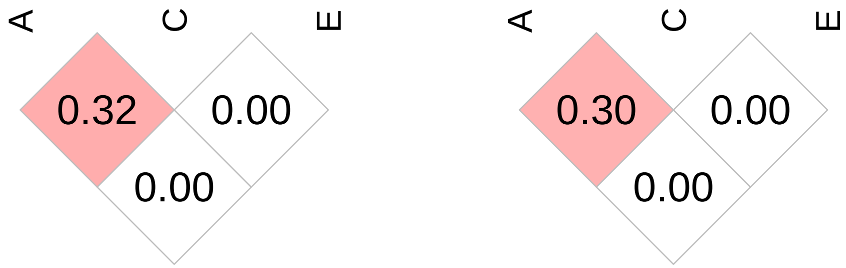

	Stepwise		Independent		Flashfm	
Traits	SNP/Model	P-value	Model	PP	Model	PP
lowdlipo	19:45437250/O	7.08139e-19	D+H+N+O	0.095	D+H+N+O	0.126
	19:45513417/D	1.82342e-12	D+G+H+M+O	0.093	D+G+H+M+O	0.079
	–	–	D+H+M+O	0.072	D+H+M+O	0.075
	–	–	D+F+H+M+O	0.056	D+G+H+N+O	0.052
cholesterol	19:45437250/O	1.03633e-10	D+O	0.393	D+O	0.520
	19:45517497/D	6.02608e-08	C+D+O	0.219	C+D+O	0.171
	–	–	D+H+O	0.153	D+H+O	0.119

Supplementary Table 93: Fine-mapping results for 19:45437250-45521976 by independent fine-mapping and Flashfm. Stepwise results are also given.

	SNP Group					
	C	D	F	G	H	M
Independent	4	3	1	18	17	1
	19:45451647	19:45513417	19:45519263	19:45518110	19:45480987	19:45439163
Flashfm	4	3	1	18	17	1
	19:45451647	19:45513417	19:45519263	19:45518110	19:45480987	19:45439163
	N	O				
Independent	3	3				
	19:45453353	19:45516768				
Flashfm	3	3				
	19:45453353	19:45516768				

Supplementary Table 94: SNP group sizes and representative variant for groups constructed by independent fine-mapping and by Flashfm for region 19:45437250-45521976.

Supplementary Figure 86: Average LD between SNP groups constructed under independent fine-mapping with those from flashfm for region 19:45437250-45521976.

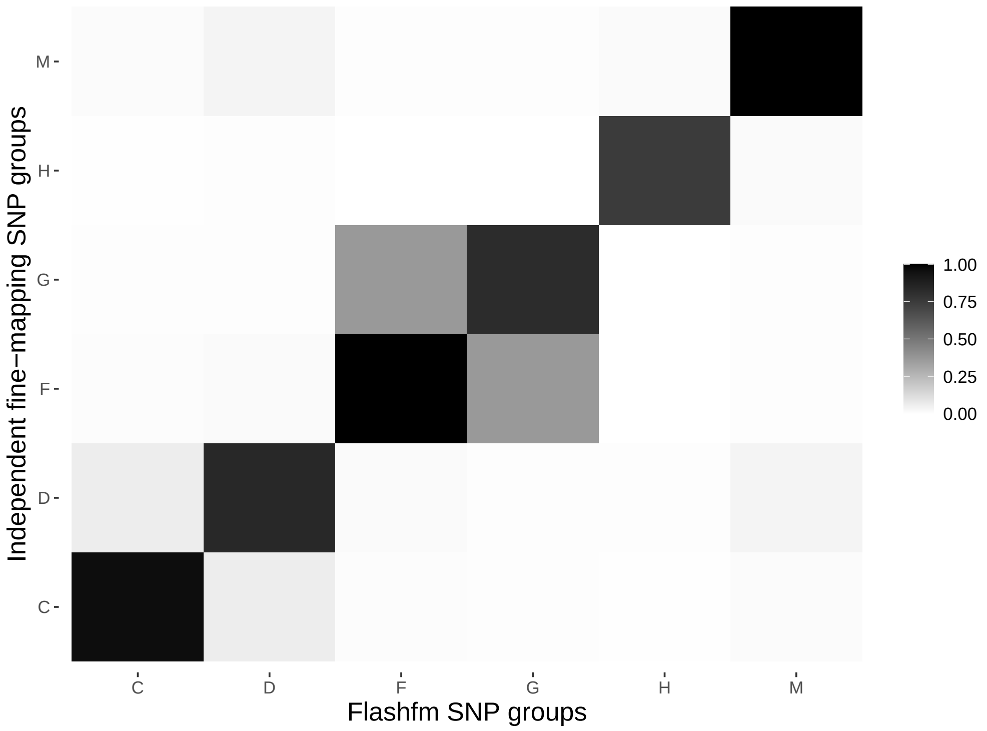

Supplementary Figure 87: Average LD between SNP groups constructed under independent fine-mapping (left) and Flashfm (right) for region 19:45437250-45521976.

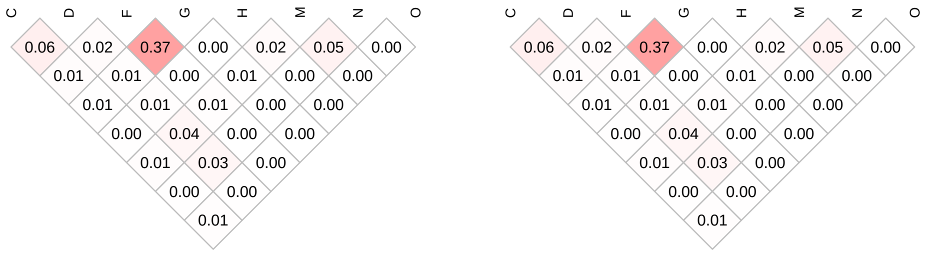

	Stepwise		Independent		Flashfm	
Traits	SNP/Model	P-value	Model	PP	Model	PP
highdlipo	19:21749298/C	2.3559e-09	C	0.695	C	0.748
eos_count	19:21800425/A	2.32322e-08	A	0.880	A	0.902

Supplementary Table 95: Fine-mapping results for 19:21431997-21822742 by independent fine-mapping and Flashfm. Stepwise results are also given.

	SNP Group	
	A	C
Independent	11	1
	19:21800425	19:21749298
Flashfm	11	1
	19:21800425	19:21749298

Supplementary Table 96: SNP group sizes and representative variant for groups constructed by independent fine-mapping and by Flashfm for region 19:21431997-21822742.

Supplementary Figure 88: Average LD between SNP groups constructed under independent fine-mapping with those from flashfm for region 19:21431997-21822742.

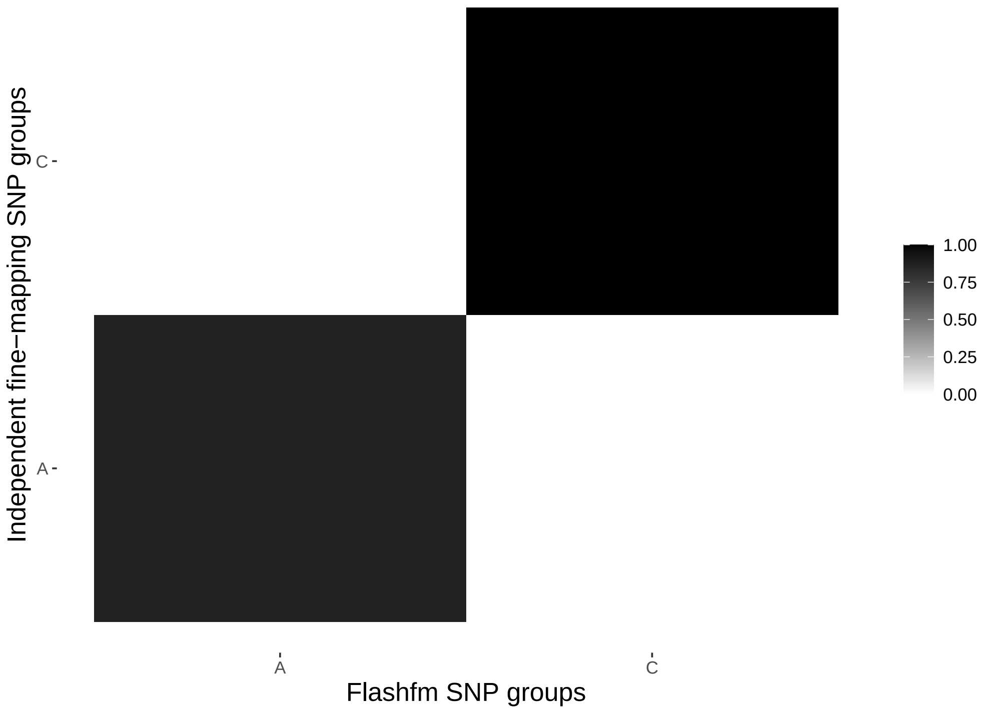

Supplementary Figure 89: Average LD between SNP groups constructed under independent fine-mapping (left) and Flashfm (right) for region 19:21431997-21822742.

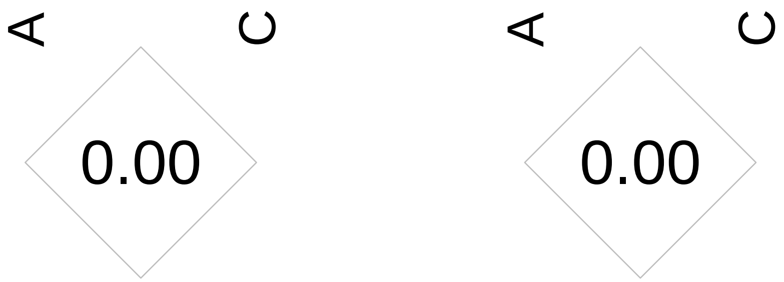

	Stepwise		Independent		Flashfm	
Traits	SNP/Model	P-value	Model	PP	Model	PP
lowdlipo	19:45412079/B	3.42883e-81	B+D2+E+L+V	0.406	B+D2+E+L+V	0.391
	19:45406538/V	3.04338e-23	B+C2+D2+E+L+V	0.279	B+C2+D2+E+L+V	0.338
	19:45410273/E	7.36491e-16	–	–	–	–
	19:45411941/L	6.51514e-13	–	–	–	–
	19:45427780/D2	3.62525e-07	–	–	–	–
cholesterol	19:45412079/B	2.3615e-38	B+E+L+V	0.265	B+E+L+V	0.418
	19:45406538/V	3.82465e-11	B+D2+E+L+V	0.256	B+D2+E+L+V	0.322
	19:45411941/L	1.02278e-09	B+E+L+N+V	0.068	B+E+L+N+V	0.06
	–	–	B+D2+E+L+N+V	0.06	–	–
triglycerides	19:45422587/I	8.78181e-15	E+E2+I+Y	0.424	E+E2+I+X	0.59
	19:45430280/X	2.88626e-07	E+E2+I+X	0.214	E+E2+I+Y	0.174
	–	–	E2+I+X	0.067	E+E2+I+W	0.056
highdlipo	19:45413576/A	1.73275e-09	A	0.382	B	0.414
	–	–	B	0.108	B+I	0.249
	–	–	A+H	0.052	B+K	0.097

Supplementary Table 97: Fine-mapping results for 19:45380937-45441453 by independent fine-mapping and Flashfm. Stepwise results are also given.

	SNP Group						
	A	B	C	D	E	H	I
Independent	1	2	1	1	5	7	1
	19:45413576	19:45400747	19:45435661	19:45392254	19:45401587	19:45418961	19:45422587
Flashfm	1	1	0	0	5	0	1
	19:45413576	19:45412079	–	–	19:45401587	–	19:45422587
	K	L	N	V	W	X	Y
Independent	3	1	2	1	1	1	3
	19:45425171	19:45411941	19:45418086	19:45406538	19:45431658	19:45430280	19:45436216
Flashfm	4	1	2	1	1	1	3
	19:45429177	19:45411941	19:45418086	19:45406538	19:45431658	19:45430280	19:45436216
	C2	D2	E2				
Independent	1	2	4				
	19:45439163	19:45427780	19:45422160				
Flashfm	1	2	3				
	19:45439163	19:45427780	19:45422160				

Supplementary Table 98: SNP group sizes and representative variant for groups constructed by independent fine-mapping and by Flashfm for region 19:45380937-45441453.

Supplementary Figure 90: Average LD between SNP groups constructed under independent fine-mapping with those from flashfm for region 19:45380937-45441453.

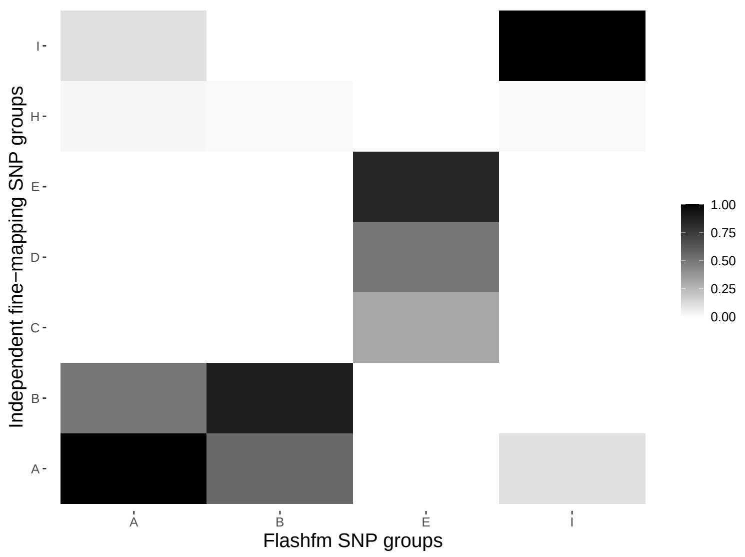

Supplementary Figure 91: Average LD between SNP groups constructed under independent fine-mapping (left) and Flashfm (right) for region 19:45380937-45441453.

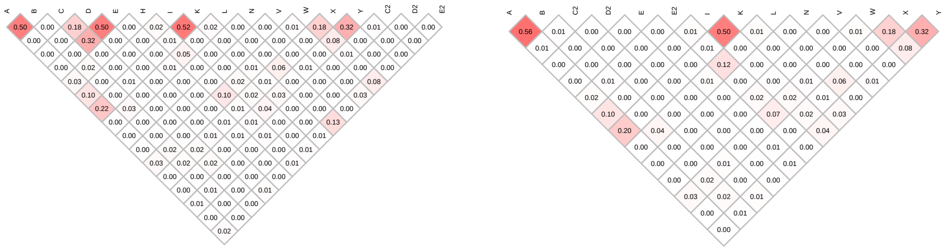

	Stepwise		Independent		Flashfm	
Traits	SNP/Model	P-value	Model	PP	Model	PP
lowdlipo	19:11197261/I	5.54756e-17	I+J	0.136	I+J	0.175
	–	–	F+I+J+M	0.126	F+I+J	0.091
	–	–	F+I+J	0.093	A.1+B+J	0.08
	–	–	A+B+J	0.079	F+I+J+M	0.076
	–	–	I+J+M	0.068	I+J+M	0.064
	–	–	G+I+J+M	0.066	A.1+J	0.061
	–	–	A+J+K	0.058	A.1+J+K	0.058
	–	–	–	–	G+I+J+M	0.05
cholesterol	19:11197261/I	2.16355e-11	I+J	0.481	I+J	0.518
	–	–	A+J	0.166	A.1+J	0.182
	–	–	F+I+J	0.061	–	–

Supplementary Table 99: Fine-mapping results for 19:11163968-11215226 by independent fine-mapping and Flashfm. Stepwise results are also given.

	SNP Group						
	A	B	F	G	I	J	K
Independent	11	2	3	1	10	5	2
	19:11189764	19:11194245	19:11171793	19:11168261	19:11197750	19:11173045	19:11207457
Flashfm	0	2	3	1	17	5	2
	–	19:11194245	19:11171793	19:11168261	19:11189764	19:11173045	19:11207457
	M	A.1					
Independent	6	0					
	19:11189047	–					
Flashfm	4	3					
	19:11189047	19:11196886					

Supplementary Table 100: SNP group sizes and representative variant for groups constructed by independent fine-mapping and by Flashfm for region 19:11163968-11215226.

Supplementary Figure 92: Average LD between SNP groups constructed under independent fine-mapping with those from flashfm for region 19:11163968-11215226.

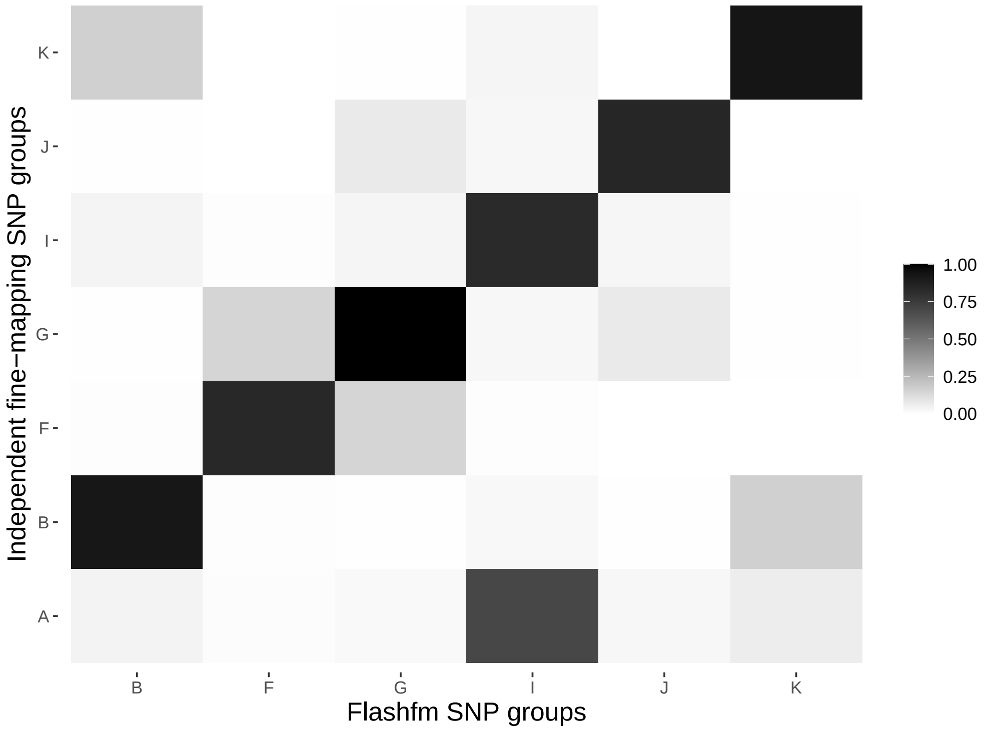

Supplementary Figure 93: Average LD between SNP groups constructed under independent fine-mapping (left) and Flashfm (right) for region 19:11163968-11215226.

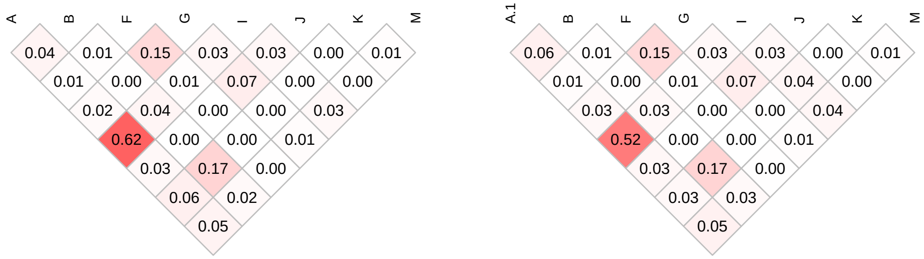

	Stepwise		Independent		Flashfm	
Traits	SNP/Model	P-value	Model	PP	Model	PP
lowdlipo	19:45397229/R	4.41906e-42	C+D+E+N+O+R	0.183	D+E+N+O+R	0.195
	19:45396665/H	4.78125e-10	D+E+N+O+R	0.146	C+D+E+N+O+R	0.160
	19:45390911/O	7.74729e-07	D+E+J+N+O+R	0.110	D+E+J+N+O+R	0.113
	–	–	C+D+E+I+N+O+R	0.076	C+D+E+I+N+O+R	0.058
cholesterol	19:45397229/R	1.17885e-18	C+O+R	0.210	C+R	0.235
	–	–	C+R	0.194	C+O+R	0.196
	–	–	C+F+O+R	0.079	C+F+R	0.074
	–	–	C+F+R	0.077	C+F+O+R	0.060

Supplementary Table 101: Fine-mapping results for 19:45359667-45397471 by independent fine-mapping and Flashfm. Stepwise results are also given.

	SNP Group						
	C	D	E	F	I	J	N
Independent	7	2	1	2	1	6	1
	19:45374350	19:45388581	19:45396240	19:45375764	19:45381420	19:45377334	19:45392254
Flashfm	7	2	1	2	1	6	1
	19:45374350	19:45388581	19:45396240	19:45375764	19:45381420	19:45377334	19:45392254
	O	R					
Independent	3	1					
	19:45390911	19:45397229					
Flashfm	3	1					
	19:45390911	19:45397229					

Supplementary Table 102: SNP group sizes and representative variant for groups constructed by independent fine-mapping and by Flashfm for region 19:45359667-45397471.

Supplementary Figure 94: Average LD between SNP groups constructed under independent fine-mapping with those from flashfm for region 19:45359667-45397471.

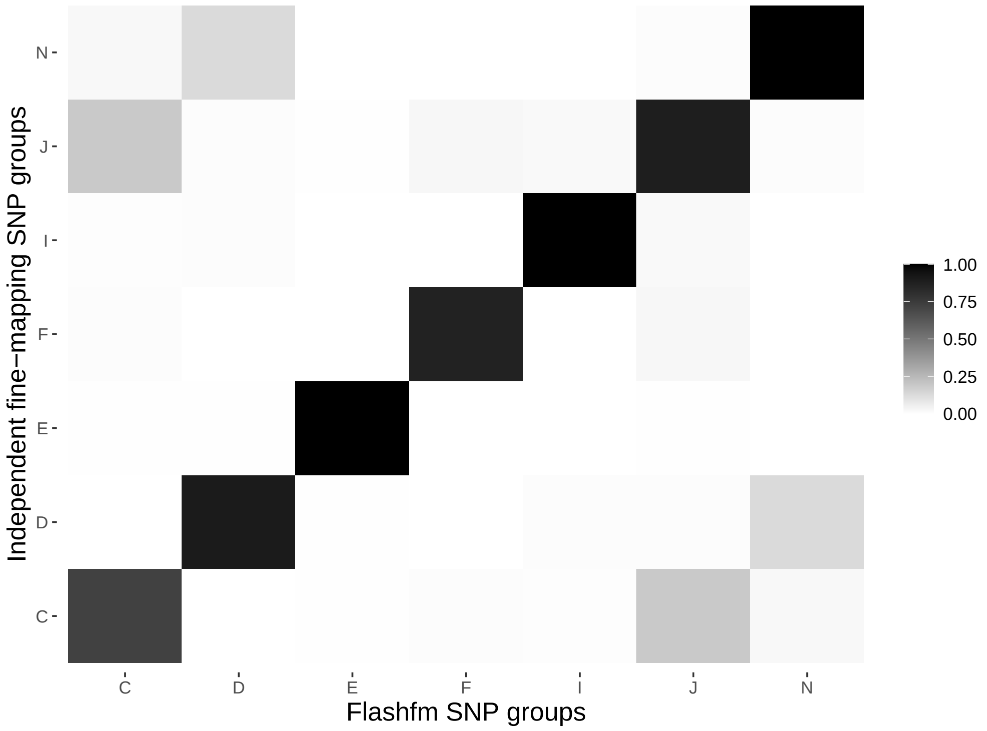

Supplementary Figure 95: Average LD between SNP groups constructed under independent fine-mapping (left) and Flashfm (right) for region 19:45359667-45397471.

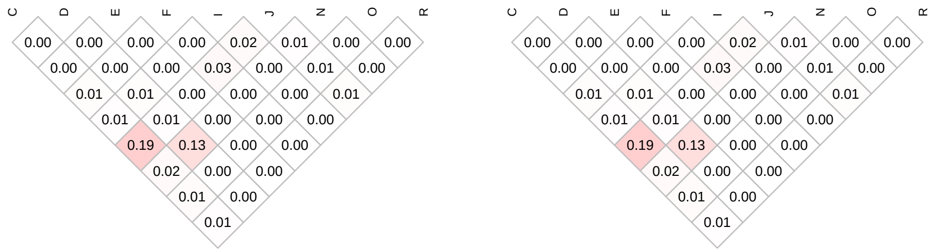

	Stepwise		Independent		Flashfm	
Traits	SNP/Model	P-value	Model	PP	Model	PP
lowdlipo	19:11197261/F	5.54756e-17	A+E+F	0.347	A+E+F	0.338
	–	–	A+F	0.194	E+F	0.177
	–	–	B+E+F	0.133	A+F	0.17
	–	–	E+F	0.121	B+E+F	0.115
	–	–	B+F	0.053	–	–
cholesterol	19:11197261/F	2.16355e-11	E+F	0.437	E+F	0.503
	–	–	A+F	0.150	F	0.16
	–	–	A+E+F	0.145	A+F	0.125
	–	–	F	0.132	A+E+F	0.1

Supplementary Table 103: Fine-mapping results for 19:10801367-11215226 by independent fine-mapping and Flashfm. Stepwise results are also given.

	SNP Group			
	A	B	E	F
Independent	24	10	13	13
	19:10837030	19:10833169	19:10801367	19:11196886
Flashfm	24	10	13	18
	19:10837030	19:10833169	19:10801367	19:11196886

Supplementary Table 104: SNP group sizes and representative variant for groups constructed by independent fine-mapping and by Flashfm for region 19:10801367-11215226.

Supplementary Figure 96: Average LD between SNP groups constructed under independent fine-mapping with those from flashfm for region 19:10801367-11215226.

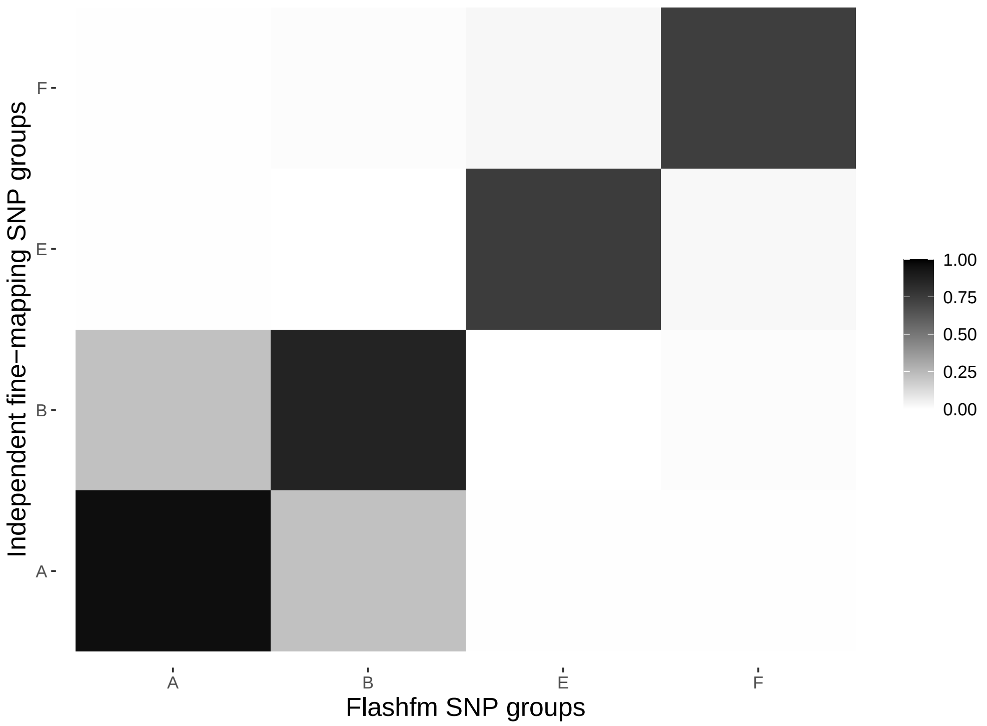

Supplementary Figure 97: Average LD between SNP groups constructed under independent fine-mapping (left) and Flashfm (right) for region 19:10801367-11215226.

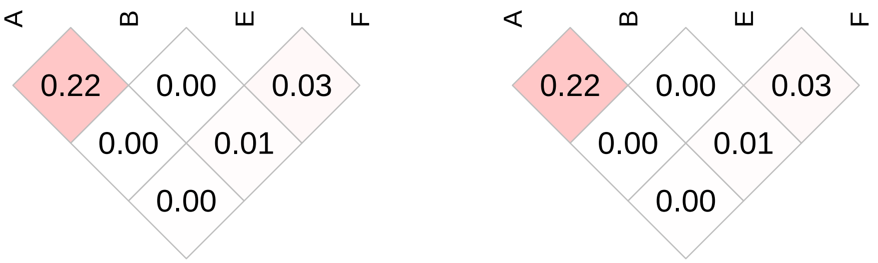

	Stepwise		Independent		Flashfm	
Traits	SNP/Model	P-value	Model	PP	Model	PP
triglycerides	19:11350488/T	3.84117e-08	E+G+H+I+O+P+V	0.136	E+G+H+I+O+P+V	0.146
	–	–	E+G+H+I+N+O+P+Y	0.107	E+G+H+I+O+P+Y	0.109
	–	–	E+G+H+I+O+P+Y	0.102	E+G+H+I+O+P	0.096
	–	–	E+G+H+I+O+P	0.073	E+G+H+I+N+O+P+Y	0.091
	–	–	E+G+H+I+N+O+P	0.073	E+G+H+I+N+O+P	0.075
	–	–	E+G+H+I+O+P+V+Y	0.059	A+E+G+H+I+O+P	0.059
	–	–	A+E+G+H+I+O+P	0.056	E+G+H+I+O+P+V+Y	0.052
	–	–	E+G+H+I+N+O+P+V	0.051	–	–
lowdlipo	19:11368341/B	1.31929e-07	B	0.538	B+H	0.69
	–	–	–	–	B	0.196
cholesterol	19:11370607/B	3.14433e-07	B	0.53	B+H	0.699
	–	–	B+M	0.08	H	0.096
	–	–	K	0.07	B	0.051

Supplementary Table 105: Fine-mapping results for 19:11299189-11405641 by independent fine-mapping and Flashfm. Stepwise results are also given.

	SNP Group						
	A	B	E	G	H	I	K
Independent	11<br>19:11393565	4<br>19:11370607	2<br>19:11309871	1<br>19:11309642	1<br>19:11313801	1<br>19:11319978	3<br>19:11368451
Flashfm	11<br>19:11393565	4<br>19:11370607	2<br>19:11309871	1<br>19:11309642	1<br>19:11313801	1<br>19:11319978	2<br>19:11366754
	M	N	O	P	V	Y	
Independent	11<br>19:11338881	14<br>19:11300862	1<br>19:11316315	1<br>19:11316547	4<br>19:11310834	3<br>19:11379717	
Flashfm	0<br>–	13<br>19:11300862	1<br>19:11316315	1<br>19:11316547	4<br>19:11310834	3<br>19:11379717	

Supplementary Table 106: SNP group sizes and representative variant for groups constructed by independent fine-mapping and by Flashfm for region 19:11299189-11405641.

Supplementary Figure 98: Average LD between SNP groups constructed under independent fine-mapping with those from flashfm for region 19:11299189-11405641.

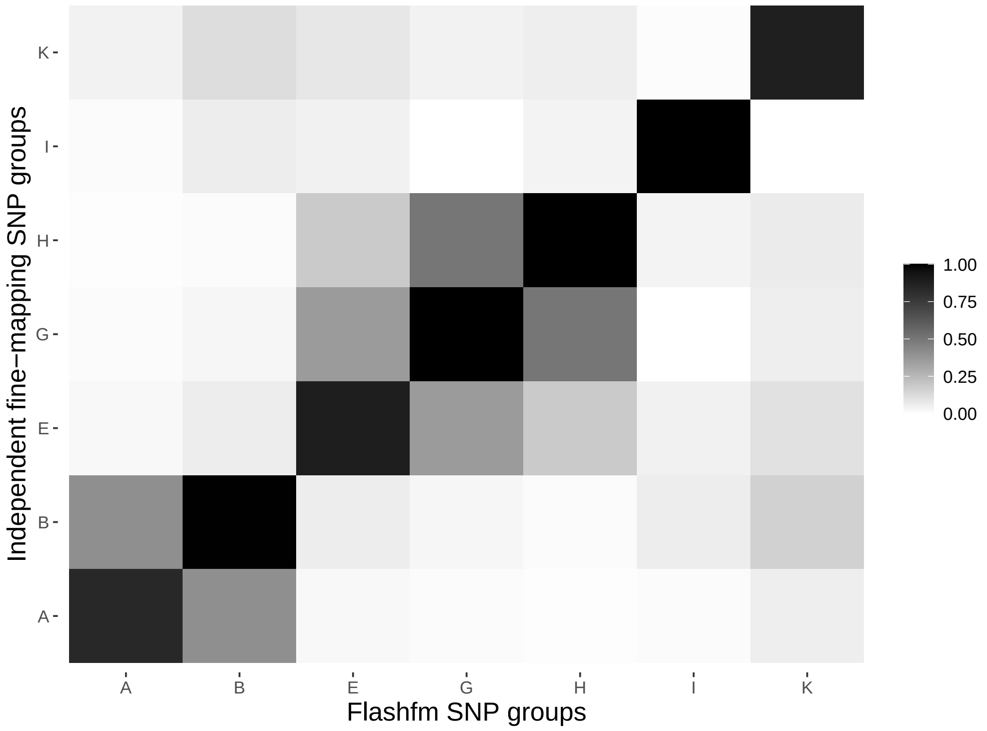

Supplementary Figure 99: Average LD between SNP groups constructed under independent fine-mapping (left) and Flashfm (right) for region 19:11299189-11405641.

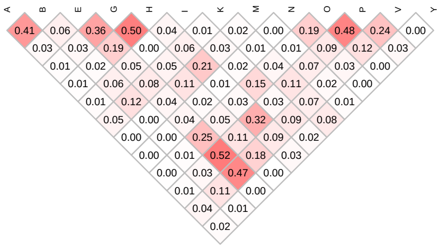

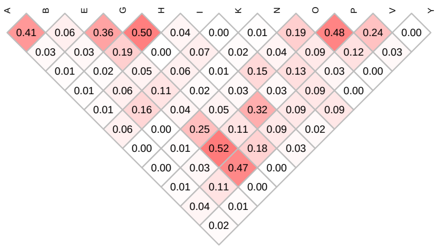

	Stepwise		Independent		Flashfm	
Traits	SNP/Model	P-value	Model	PP	Model	PP
lowdlipo	19:11281332/G	5.65495e-08	G	0.570	G	0.679
	–	–	C+G	0.127	C+G	0.086
cholesterol	19:11281332/G	2.11929e-07	G	0.735	G	0.811

Supplementary Table 107: Fine-mapping results for 19:11277607-11283222 by independent fine-mapping and Flashfm. Stepwise results are also given.

	SNP Group	
	C	G
Independent	2	1
	19:11279456	19:11281332
Flashfm	2	1
	19:11279456	19:11281332

Supplementary Table 108: SNP group sizes and representative variant for groups constructed by independent fine-mapping and by Flashfm for region 19:11277607-11283222.

Supplementary Figure 100: Average LD between SNP groups constructed under independent fine-mapping with those from flashfm for region 19:11277607-11283222.

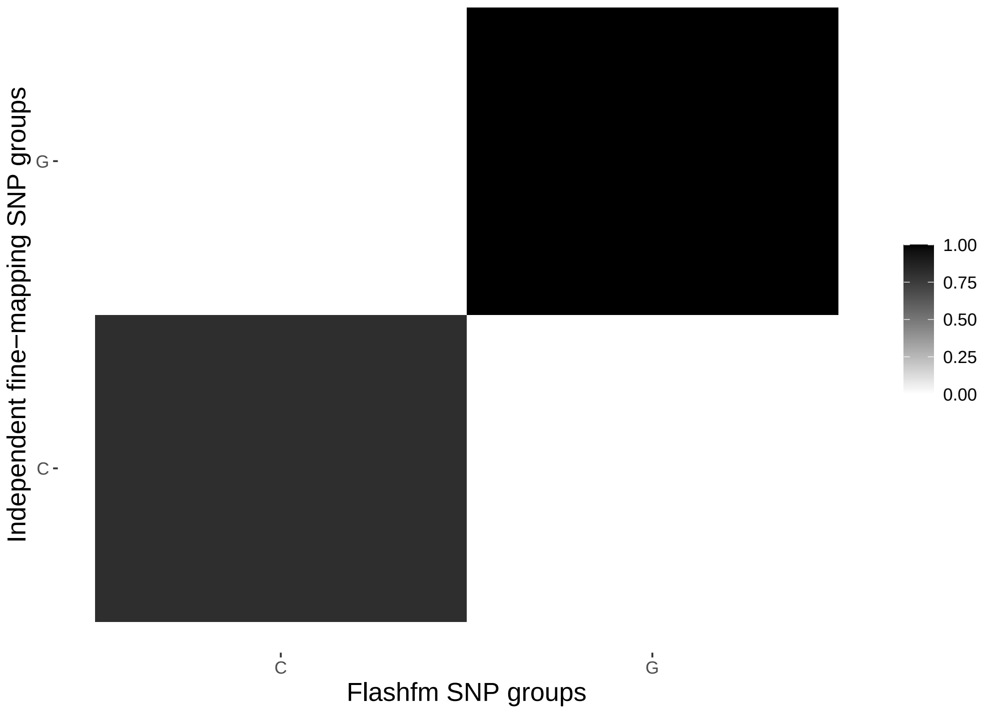

Supplementary Figure 101: Average LD between SNP groups constructed under independent fine-mapping (left) and Flashfm (right) for region 19:11277607-11283222.

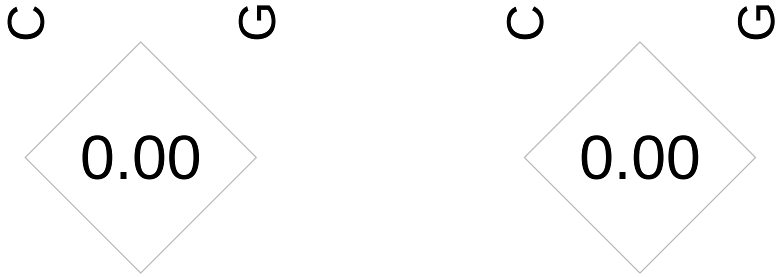

	Stepwise		Independent		Flashfm	
Traits	SNP/Model	P-value	Model	PP	Model	PP
highdlipo	20:22468476/H	2.67091e-08	H	0.224	F.1+H	0.321
	–	–	C+H	0.21	H	0.204
	–	–	F+H	0.197	C+H	0.134
	–	–	G	0.057	C+F.1+H	0.062
cholesterol	20:22450576/F	3.05885e-07	F	0.743	F.1	0.782
	–	–	–	–	F.1+H	0.057

Supplementary Table 109: Fine-mapping results for 20:22424162-22486585 by independent fine-mapping and Flashfm. Stepwise results are also given.

	SNP Group				
	C	F	G	H	F.1
Independent	21	48	7	1	0
	20:22452490	20:22476028	20:22482193	20:22468476	–
Flashfm	21	0	7	1	18
	20:22452490	–	20:22482193	20:22468476	20:22450576

Supplementary Table 110: SNP group sizes and representative variant for groups constructed by independent fine-mapping and by Flashfm for region 20:22424162-22486585.

Supplementary Figure 102: Average LD between SNP groups constructed under independent fine-mapping with those from flashfm for region 20:22424162-22486585.

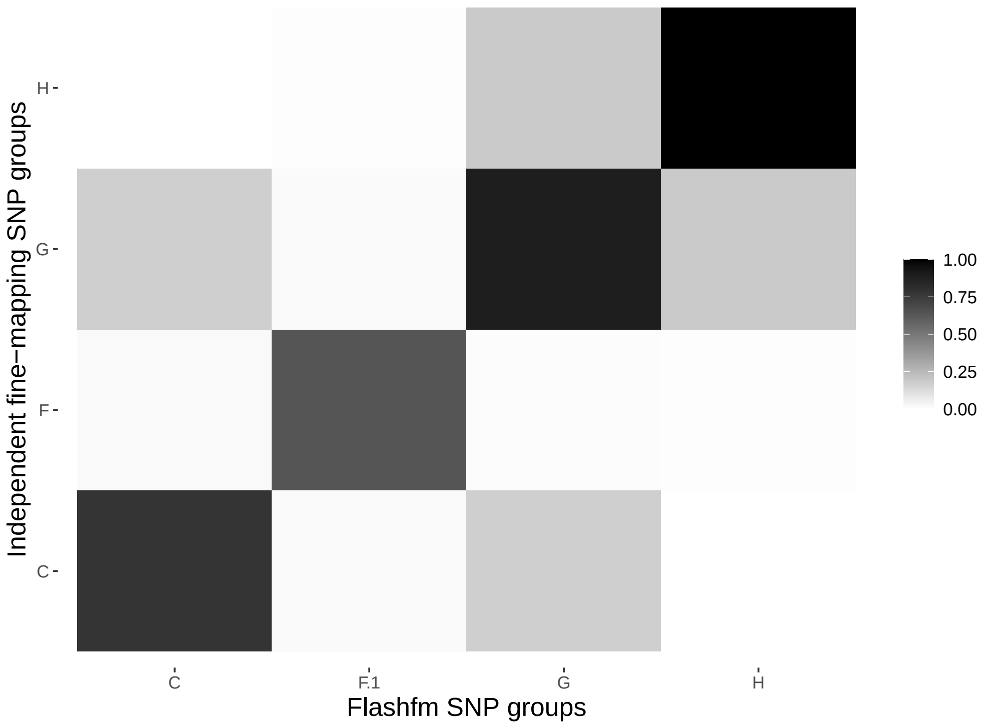

Supplementary Figure 103: Average LD between SNP groups constructed under independent fine-mapping (left) and Flashfm (right) for region 20:22424162-22486585.

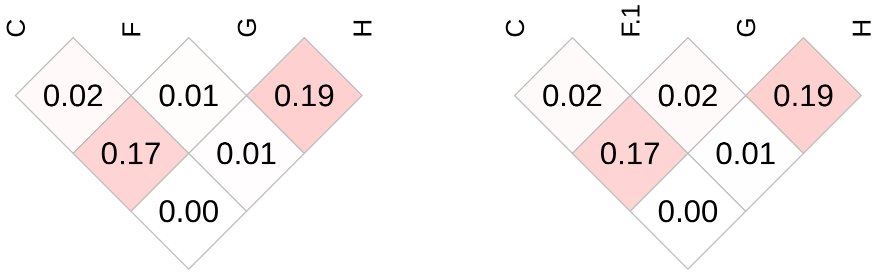

	Stepwise		Independent		Flashfm	
Traits	SNP/Model	P-value	Model	PP	Model	PP
hgb	22:34741507/B	5.78131e-08	B	0.894	B	0.921
rbc	22:34724587/A	7.44263e-08	A	0.336	A	0.747
	–	–	B	0.335	–	–
	–	–	B+E	0.064	–	–
hct	22:34741507/B	1.1751e-07	B	0.883	B	0.816
	–	–	–	–	A	0.098

Supplementary Table 111: Fine-mapping results for 22:34644471-34814543 by independent fine-mapping and Flashfm. Stepwise results are also given.

	SNP Group		
	A	B	E
Independent	3	47	4
	22:34706503	22:34792119	22:34672865
Flashfm	2	27	0
	22:34724587	22:34746385	–

Supplementary Table 112: SNP group sizes and representative variant for groups constructed by independent fine-mapping and by Flashfm for region 22:34644471-34814543.

Supplementary Figure 104: Average LD between SNP groups constructed under independent fine-mapping with those from flashfm for region 22:34644471-34814543.

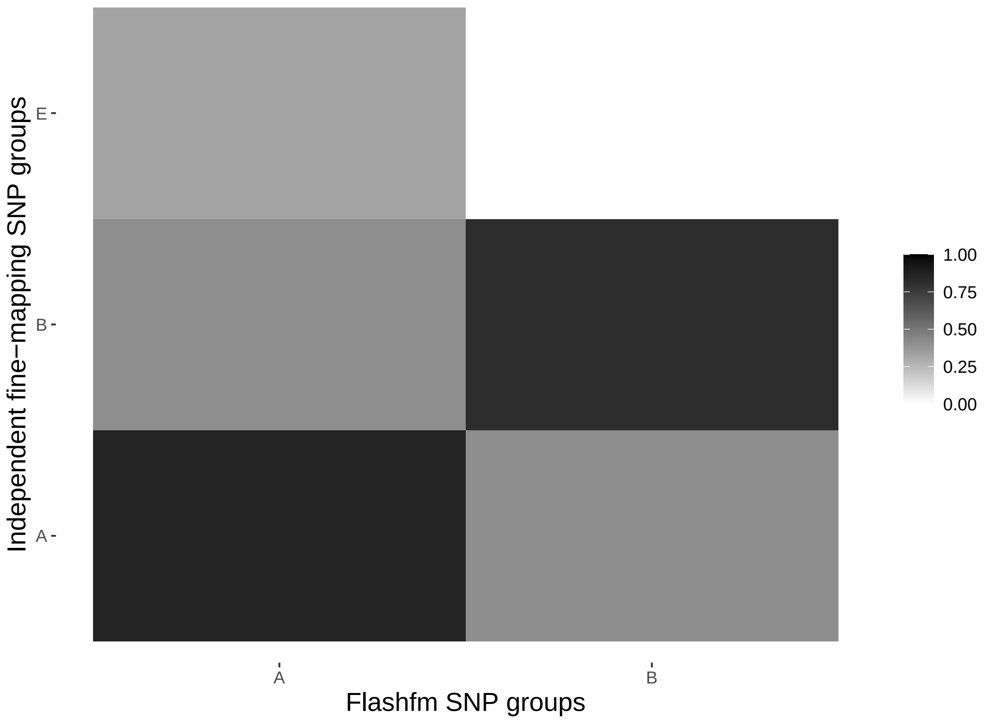

Supplementary Figure 105: Average LD between SNP groups constructed under independent fine-mapping (left) and Flashfm (right) for region 22:34644471-34814543.

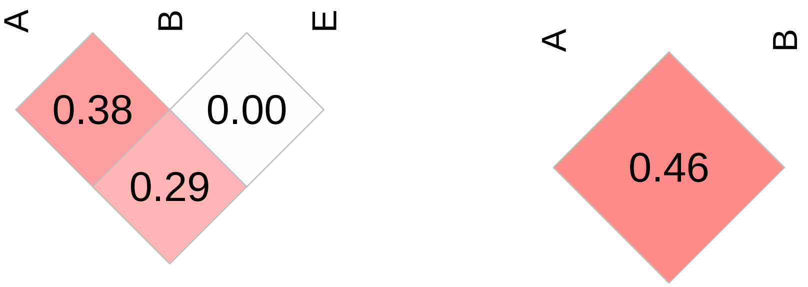

Supplement: Supplementary file 5 — Supplementary Data 2 [file 41467_2021_26364_MOESM5_ESM.pdf]
