# Supplementary material for: The flashfm approach for fine-mapping multiple quantitative traits
Source: Nat Commun. 2021 Oct 22;12:6147. doi: 10.1038/s41467-021-26364-y (PMC8536717; doi:10.1038/s41467-021-26364-y)
Supplement: Supplementary file 8 — Reporting Summary [file 41467_2021_26364_MOESM8_ESM.pdf]

nature research

Corresponding author(s): Jennifer Asimit

Last updated by author(s): Sep 18, 2021

Reporting Summary

Nature Research wishes to improve the reproducibility of the work that we publish. This form provides structure for consistency and transparency in reporting. For further information on Nature Research policies, see our Editorial Policies and the Editorial Policy Checklist.

Statistics

For all statistical analyses, confirm that the following items are present in the figure legend, table legend, main text, or Methods section.

n/a	Confirmed	
☐	☒	The exact sample size (n) for each experimental group/condition, given as a discrete number and unit of measurement
☒	☐	A statement on whether measurements were taken from distinct samples or whether the same sample was measured repeatedly
☐	☒	The statistical test(s) used AND whether they are one- or two-sided<br>*Only common tests should be described solely by name; describe more complex techniques in the Methods section.*
☐	☒	A description of all covariates tested
☐	☒	A description of any assumptions or corrections, such as tests of normality and adjustment for multiple comparisons
☐	☒	A full description of the statistical parameters including central tendency (e.g. means) or other basic estimates (e.g. regression coefficient) AND variation (e.g. standard deviation) or associated estimates of uncertainty (e.g. confidence intervals)
☐	☒	For null hypothesis testing, the test statistic (e.g. F, t, r) with confidence intervals, effect sizes, degrees of freedom and P value noted<br>*Give P values as exact values whenever suitable.*
☐	☒	For Bayesian analysis, information on the choice of priors and Markov chain Monte Carlo settings
☒	☐	For hierarchical and complex designs, identification of the appropriate level for tests and full reporting of outcomes
☒	☐	Estimates of effect sizes (e.g. Cohen's d, Pearson's r), indicating how they were calculated

Our web collection on statistics for biologists contains articles on many of the points above.

Software and code

Policy information about availability of computer code

Data collection

no software was used for data collection.

Data analysis

Our proposed multi-trait fine-mapping method, Flexible and shared information fine-mapping (flashfm), is freely available as an R library at https://jennasimit.github.io/flashfm/ (DOI: 10.5281/zenodo.552291545). Single-trait fine-mapping was performed with FINEMAP 1.4 (http://www.christianbenner.com/), as well as our extended version of JAM (based on JAM from R2BGLiMS; https://github.com/pjnewcombe/R2BGLiMS) that is included in the flashfm package. Custom code for the analysis of the Ugandan data is available at https://github.com/nicolashernandezb/flashfm-analysis. The annotation tools we used are HaploReg v4.1 (https://pubs.broadinstitute.org/mammals/haploreg/haploreg.php) and Ensembl Variant Effect Predictor (VEP) GRCh37 (https://grch37.ensembl.org/info/docs/tools/vep/index.html). We simulated genotype data with hapgen2 (http://mathgen.stats.ox.ac.uk/genetics_software/hapgen/hapgen2.html).

For manuscripts utilizing custom algorithms or software that are central to the research but not yet described in published literature, software must be made available to editors and reviewers. We strongly encourage code deposition in a community repository (e.g. GitHub). See the Nature Research guidelines for submitting code & software for further information.

Data

Policy information about availability of data

All manuscripts must include a data availability statement. This statement should provide the following information, where applicable:

- Accession codes, unique identifiers, or web links for publicly available datasets
- A list of figures that have associated raw data
- A description of any restrictions on data availability

Detailed flashfm multi-trait fine-mapping results and FINEMAP single-trait fine-mapping results for the Ugandan cardiometabolic traits are provided in Supplementary Data 3 and 4, respectively; summary fine-mapping results are provided in Supplementary Data2.pdf. The Uganda GWAS data used in this study are

available in the GWAS Catalog under PubMed ID 31675503 (https://www.ebi.ac.uk/gwas/publications/31675503#study_panel). The Ugandan genotype data are from the European Genome-phenome Archive (EGA) under accession numbers EGAS00001001558 (https://ega-archive.org/studies/EGAS00001001558)/ EGAD00010000965 (https://ega-archive.org/datasets/EGAD00010000965), EGAS00001000545 (https://ega-archive.org/studies/EGAS00001000545)/ EGAD00001001639 (https://ega-archive.org/datasets/EGAD00001001639). The phenotype data used in this study are not under restricted access and requests for access to data may be directed to segun.fatumo@mrcuganda.org. The CEU population 1000 Geomes phase 3 haplotype data that were used in our simulations are available from http://grch37.ensembl.org/Homo_sapiens/Tools/DataSlicer.

Field-specific reporting

Please select the one below that is the best fit for your research. If you are not sure, read the appropriate sections before making your selection.

☒ Life sciences ☐ Behavioural & social sciences ☐ Ecological, evolutionary & environmental sciences

For a reference copy of the document with all sections, see nature.com/documents/nr-reporting-summary-flat.pdf

Life sciences study design

All studies must disclose on these points even when the disclosure is negative.

Sample size	We used all the samples available with genotype data from the largest African study done to date.
Data exclusions	No data were excluded.
Replication	There was no replication, as there is no gold standard for replication of fine-mapping results. We identify likely causal variants and provide posterior probabilities of support for groups of genetic variants. For LDL and APOE, we replicate the result of two causal variants that have been previously established.
Randomization	This was not relevant to our study as individuals were not assigned to groups.
Blinding	Blinding was not relevant for the same reasons as above.

Reporting for specific materials, systems and methods

We require information from authors about some types of materials, experimental systems and methods used in many studies. Here, indicate whether each material, system or method listed is relevant to your study. If you are not sure if a list item applies to your research, read the appropriate section before selecting a response.

Materials & experimental systems

n/a	Involved in the study	
☒	☐	Antibodies
☒	☐	Eukaryotic cell lines
☒	☐	Palaeontology and archaeology
☒	☐	Animals and other organisms
☒	☐	Human research participants
☒	☐	Clinical data
☒	☐	Dual use research of concern

Methods

n/a	Involved in the study	
☒	☐	ChIP-seq
☒	☐	Flow cytometry
☒	☐	MRI-based neuroimaging
